# New Mid-Cretaceous (Latest Albian) Dinosaurs from Winton, Queensland, Australia

**DOI:** 10.1371/journal.pone.0006190

**Published:** 2009-07-03

**Authors:** Scott A. Hocknull, Matt A. White, Travis R. Tischler, Alex G. Cook, Naomi D. Calleja, Trish Sloan, David A. Elliott

**Affiliations:** 1 Geosciences, Queensland Museum, Hendra, Queensland, Australia; 2 Australian Age of Dinosaurs Museum of Natural History, The Jump-up, Winton, Queensland, Australia; University of Chicago, United States of America

## Abstract

**Background:**

Australia's dinosaurian fossil record is exceptionally poor compared to that of other similar-sized continents. Most taxa are known from fragmentary isolated remains with uncertain taxonomic and phylogenetic placement. A better understanding of the Australian dinosaurian record is crucial to understanding the global palaeobiogeography of dinosaurian groups, including groups previously considered to have had Gondwanan origins, such as the titanosaurs and carcharodontosaurids.

**Methodology/Principal Findings:**

We describe three new dinosaurs from the late Early Cretaceous (latest Albian) Winton Formation of eastern Australia, including; *Wintonotitan wattsi* gen. et sp. nov., a basal titanosauriform; *Diamantinasaurus matildae* gen. et sp. nov., a derived lithostrotian titanosaur; and *Australovenator wintonensis* gen. et sp. nov., an allosauroid. We compare an isolated astragalus from the Early Cretaceous of southern Australia; formerly identified as *Allosaurus* sp., and conclude that it most-likely represents *Australovenator* sp.

**Conclusion/Significance:**

The occurrence of *Australovenator* from the Aptian to latest Albian confirms the presence in Australia of allosauroids basal to the Carcharodontosauridae. These new taxa, along with the fragmentary remains of other taxa, indicate a diverse Early Cretaceous sauropod and theropod fauna in Australia, including plesiomorphic forms (e.g. *Wintonotitan* and *Australovenator*) and more derived forms (e.g. *Diamantinasaurus*).

## Introduction

**Figure 1:**
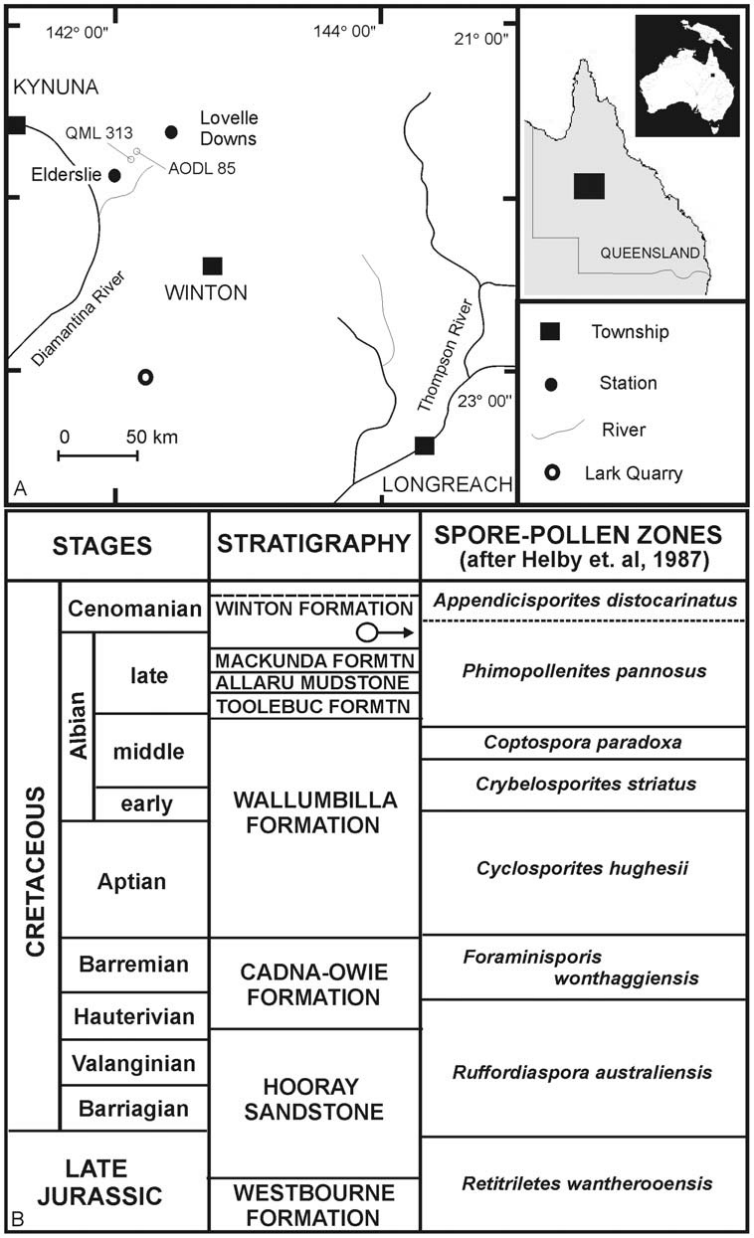
Locality Map and Stratigraphy. Showing the locations of QML 313 and AODL 85, northwest of Winton, Queensland, Australia (A). Cretaceous Stratigraphy of the Eromanga Basin, Queensland. Litho- and palynostratigraphy of the Cretaceous portion of the Eromanga Basin, Queensland. Arrow indicates the position of the type localities QML 313 and AODL 85 within the sequence (B). Adapted from [8].

Australia's dinosaur fossil record is extremely poor relative to faunas recovered from similar-sized land-masses (e.g. North America, South America and Africa). This poor record is in spite of extensive Mesozoic-aged sedimentary basins being available across the continent and dinosaur remains having been found in most of these; in particular those of Early Cretaceous age [1]–[3]. Recent exploration and discoveries in central Queensland's mid-Cretaceous Winton Formation have yielded numerous new fossil sites with enormous potential for the discovery of new dinosaurian taxa [4], [5] (Figure 1). Previous excavations have yielded many specimens, however, very few elements are considered to be associated to a single individual or taxon, thus making identification and description of taxa difficult. Intensive excavations between 2006 and 2009 in central Queensland by the Australian Age of Dinosaurs Museum of Natural History and the Queensland Museum has yielded large quantities of well-preserved dinosaur fossils along with the remains of other contemporaneous fauna (Table 1) and flora [6]–[8].

**Table 1:** Latest Albian (mid-Cretaceous) fauna currently known from the Winton Formation.

Type	Taxon	Interpretation	Reference
Fauna			
Ichnofossil	*Tyrannosauropus* sp.	Theropod	[41]
Ichnofossil	*Skartopus australis*	Theropod	[41]
Ichnofossil	*Wintonopus latomorum*	ornithopod	[41]
Ichnofossil	Large ornithopod	Large ornithopod	[41]
Hyriidae	*Megalovirgus wintonensis*	Freshwater mollusc	[42], [43]
Hyriidae	*Hyridella goodiwindiensis*	Freshwater mollusc	[42], [43]
Hyriidae	*Prohyria macmichaeli*	Freshwater mollusc	[42], [43]
Gastropod		Freshwater snail	[44]
Insect		Cicadid	[45]
Lungfish	*Metaceratodus ellioti*	Lungfish	[46]
Squamate	cf. *Coniasaurus*	Dolichosaur lizard	[47]
Eusuchian	*Isisfordia duncani*	crocodilian	[48]
Chelonian	Indet. ?chelid	Turtle	Hocknull pers. obs.
Pterodactyloid	Indet. pterosaur	Pterosaur	Hocknull pers. obs.
Sauropoda	“*Austrosaurus* sp.”	Titanosauriform	[49]
Ornithopoda	hypsilophodontid	Small ornithopod	[50]
Ankylosauria		Small ankylosaur	Hocknull pers. obs.

We report on two sites in particular that have yielded the remains of three individual dinosaur skeletons representing three distinct taxa; two new sauropods and a new theropod – the most complete theropod skeleton so far found in Australia.

### Geological Settings

The fossil remains described here are derived from the Winton Formation, which is the upper-most formation of the Mesozoic-aged Eromanga Basin [9] (Figure 1). The Winton Formation is approximately 1100 m thick and is ascribed a late Albian-Cenomanian age by Burger [10]. This was based on the extensive formation spanning two palynomorph zones; the lower section occurring in the upper *Phimopollenites pannosus* palynomorph Zone (Albian) and the upper section occurring in the lower *Appendicisporites distocarinatus* Zone (Cenomanian).

The dinosaur remains described here were excavated from the basal portion of the Winton Formation and are close by (∼3 km) the type locality for *Lovellea wintonensis* [8], a silicified angiosperm flora associated with pollens. These pollens indicate a *Phimopollenites pannosus* palynomorph Zone sequence and have been suggested as latest Albian in age [8]. Therefore, we consider the age of the dinosaur remains to be latest Albian in age. A recent appraisal of the age and interpreted geological settings of the Winton Formation is available [7], [8], [11].

### Winton Formation

Terrestrial and aquatic vertebrate fossil remains were recovered from fine-grained siltstones, labile sandstones and claystones derived from within the Winton Formation and interpreted here to have been formed in a distal fluvial depositional environment. Two localities were initially discovered by the presence of bone fragments on the surface of the soil, and with excavation, revealed the presence of Winton Formation at a depth of less than 1 m below the ground surface. Within this Winton Formation the preserved remains of three associated dinosaur skeletons were recovered. The two sites are approximately 3 kms from one another and are only two of at least five sites so far known to reveal dinosaur remains from this one location. Other localities in the district have also yielded faunal remains typical of the Winton Formation (Table 1).

### Depositional Environment

Detailed sedimentologic and taphonomic information collected from both sites will form part of a more extensive study and will be published elsewhere. Preliminary investigations indicate that the AODL 85 ‘Matilda Site’ deposit accumulated as part of a low energy, silt-rich, abandoned channel fill deposit, most likely as part of an ox-bow lake, with distinctive ‘billabong’ morphology. The depositional environment at QML 313 “Triangle Paddock Site” was a higher energy deposit and is considered to be a point-bar sequence. Excavation at both type localities revealed the presence of disarticulated, semi-articulated and associated skeletal elements of the three dinosaurs. Faunal remains recovered during excavations at AODL 85 include two partial dinosaur skeletons, a sauropod and theropod, along with the fossil remains of fish, crocodiliforms, turtles and hyriid bivalves. Fossil remains from AODL 85 are well preserved within a fine-grained clay sediment bounded by upper and lower labile sandstone horizons. Macrofloral remains have been recovered from within the lower clay sequence and within the sequence preserving the faunal remains. The macroflora assemblage includes angiosperms, auracarian gymnosperms, ginkgoes and ferns.

Faunal remains recovered from QML 313 include the semi-articulated and associated skeleton of a sauropod, along with fragmentary remains of fish and an isolated theropod tooth. Most of the sauropod bone elements have been abraded prior to deposition; however, all are clearly associated with a single individual. Fossil remains from QML 313 are preserved in coarsely bedded sandstone overlying fine-grained clay. Macrofloral remains include mostly woody stems and branch impressions; however, auracarian cones, cone scales and pinnae are recognisable.

## Methods

### Fossil Preparation

The holotype specimens were prepared using pneumatic air scribes, pneumatic chisels, high speed diamond tipped rotary tools and high speed diamond wheel cutters. The majority of the sauropod elements were encased in an iron-oxide crust which preserve evidence of pyritic pseudomorphs; some iron-oxide crusts were up to 10 cm thick. Several elements, including many of the theropod bones were encased in a concretionary phosphatic crust.

### Terminology

The terms ‘anterior’ and ‘posterior’ are used with respect to the placement of postcranial elements (e.g. anterior caudal vertebra; posterior caudal vertebra; anterior dorsal rib; posterior dorsal rib) and when describing the aspect of the view (e.g. “In anterior view”).

For individual postcranial elements, the terms ‘cranial, caudal, lateral, medial, proximal and distal’ are used to identify the region of the element being described. For sauropod metacarpals the terms ‘external’ and ‘internal’ are used to differentiate the surface of the metacarpal exposed externally of the manus and those surfaces which are enclosed (internal) within the manus. We use previously developed terminology for teeth [12] and vertebral laminae [13].

### Phylogenetic Analysis

All phylogenetic analyses were conducted using parsimony analysis software, PAUP 4.0 [14]. Each analysis was conducted with the following settings: 1. All characters unordered; 2. Outgroup taxa monophyletic with respect to ingroup taxa; 3. Heuristic Search using the tree bisection and reconnection algorithm (TBR), random seed generator; 4. 1000 TBR replicates to find the most- parsimonious tree; 5. Most parsimonious-trees (MPT) saved and a strict consensus tree generated of these MPTs; 6. 1000 bootstrap replicates of this analysis with nodes returning values >50% recorded.

The electronic version of this document does not represent a published work according to the International Code of Zoological Nomenclature (ICZN), and hence the nomenclatural acts contained herein are not available under that Code from the electronic edition. A separate edition of this document was produced by a method that assures numerous identical and durable copies, and those copies were simultaneously obtainable (from the publication date listed on page 1 of this paper) for the purpose of providing a public and permanent scientific record, in accordance with Article 8.1 of the Code. The separate print-only edition is available on request from PLoS by sending a request to PLoS ONE, 185 Berry Street, Suite 3100, San Francisco, CA 94107, USA along with a check for $10 (to cover printing and postage) payable to “Public Library of Science.

The online version of the article is archived and available from the following digital repositories: PubMedCentral (www.pubmedcentral.nih.gov/), LOCKSS (http://www.lockss.org/lockss/), Queensland Museum Library (www.qm.qld.gov.au) and Australian Age of Dinosaurs Library (www.australianageofdinosaurs.com.au). In addition, this published work and the nomenclatural acts it contains have been registered in ZooBank (http://www.zoobank.org/), the proposed online registration system for the ICZN. The ZooBank LSIDs (Life Science Identifiers) can be resolved and the associated information viewed through any standard web browser by appending the LSID to the prefix http://zoobank.org/. The LSID Number at Zoobank, for this publication is: urn:lsid:zoobank.org:pub:E02E6156-CB22-4952-9E9A-8C6ED7377B40

### Institutional Abbreviations

**QMF** Queensland Museum Fossil
**QML** Queensland Museum Locality
**AODF** Australian Age of Dinosaurs Fossil
**AODL** Australian Age of Dinosaurs Locality
**NMVP** Museum of Victoria Palaeontological Collection
**FPMN** Fukui Prefectural Museum

## Results

### Systematic Palaeontology

Systematic Hierarchy:

Dinosauria Owen, 1842
Saurischia Seeley, 1887
Sauropodomorpha, Huene, 1932
Titanosauriformes, Salgado et al., 1997
Titanosauria, Bonaparte & Coria, 1993
Lithostrotia, Upchurch et al., 2004
*Incertae sedis*

*Diamantinasaurus* gen. nov.

urn:lsid:zoobank.org:act:7A72845C-7E28-4C1E-B536-F37D8F270F5D

#### Etymology

*Diamantina*, in reference to the Diamantina River which runs near the type locality. *sauros*, Greek for lizard.

Type species. *Diamantinasaurus matildae*

*Diamantinasaurus matildae* sp. nov.

urn:lsid:zoobank.org:act:6BE2173E-5438-4F9A-8978-745C3379F90D

#### Etymology

For *Matilda*, in reference to “Waltzing Matilda”, one of Australia's National songs, written by Banjo Patterson in Winton (“Matilda Country”) in 1895.

#### Holotype

**Figure 2:**
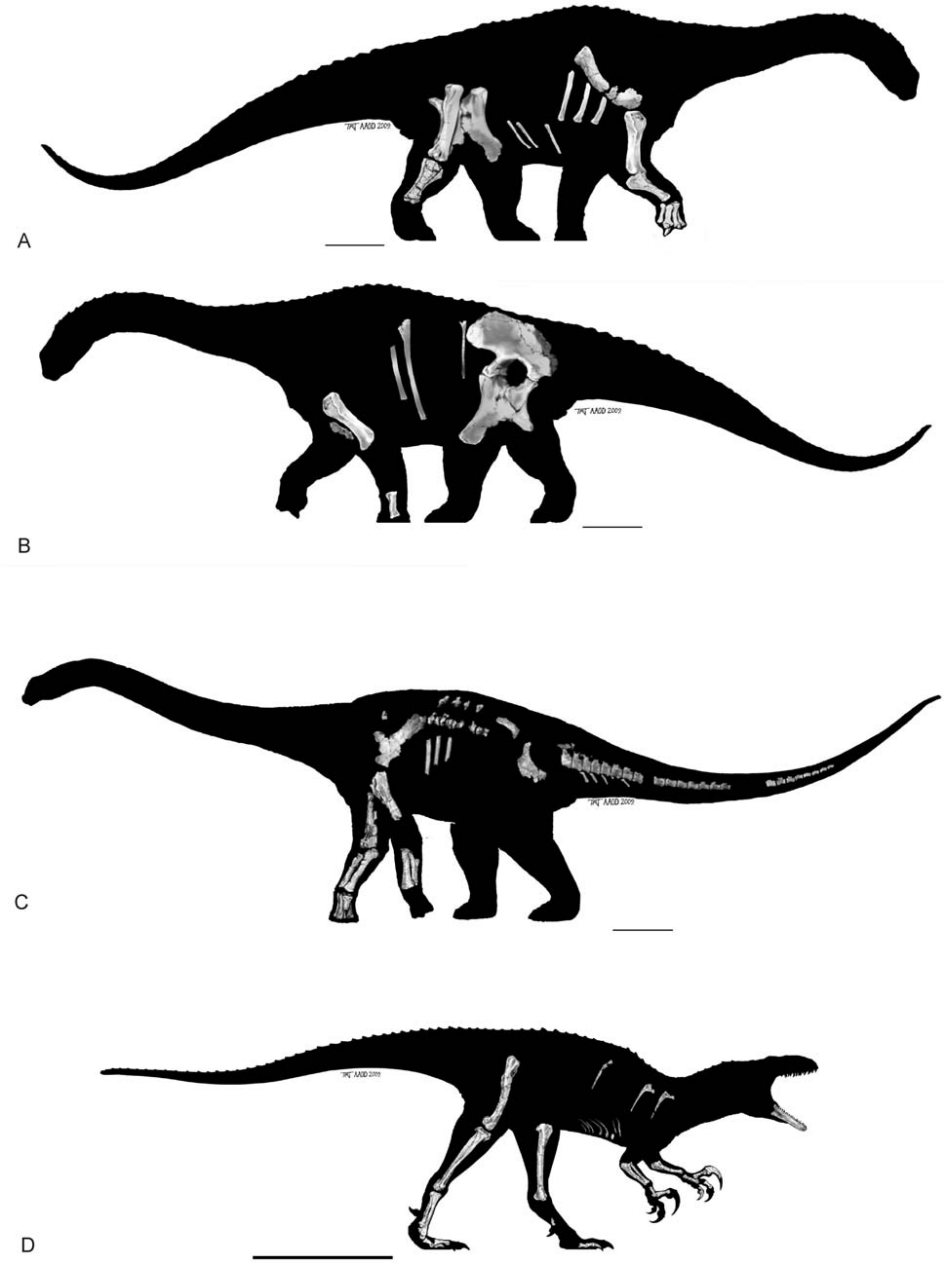
Silhouettes of the three new dinosaurs showing the material currently known from their respective holotypes. A–B. *Diamantinasaurus matildae* gen. et sp. nov. (AODF 603); A. Right side, B. Left side. C. *Wintonotitan wattsi* gen. et sp. nov. (QMF 7292); Left view. D. *Australovenator wintonensis* gen. et sp. nov. (QMF 7292); Right view. Artwork: T. Tischler, Australian Age of Dinosaurs Museum of Natural History.

AODF 603: Right scapula, right and left humeri, right ulna, near complete right metacarpus including metacarpals II–V, phalanges and a manus ungual. Left metacarpal I. Dorsal ribs and fragmentary gastralia. Left sternal plate. Left ilium and isolated sacral processes. Right and left pubes and ischia. Right femur, tibia, fibula and astragalus (Figure 2A–B).

#### Type Locality

AODL 85, “Matilda Site”, Elderslie Station, approximately 60 km north-west of Winton, western central Queensland, Australia.

#### Horizon & Age

Winton Formation, latest Albian (Cretaceous).

#### Diagnosis

*Diamantinasaurus matildae* gen. et sp. nov. is characterised by the following unique association of features. Dorsal ribs plank-like with camellate pneumatic cavities in the proximal-most expansion. Transverse processes of the sacral vertebrae with camellate pneumatic cavities. Scapular glenoid bevelled medially and expanded medio-laterally; scapular blade flat and rectangular in cross-section; acromial blade broad and dish-shaped with poorly developed acromial ridge. Crescentic-shaped sternal plate. Humerus stout; intermediate robusticity (autapomorphic); deltopectoral crest prominent, extending to mid-shaft; proximal border shallowly sigmoidal; proximo-lateral corner square; distal condyles merged and flat extending only just onto anterior face of shaft; proximal breadth 50% of total length. Ulna stout; cranio-medial process massive and concave; distal condyle ovo-triangular. Metacarpals massive with undivided condyles and marked distal rugosities; Mc III longest followed by Mc II, Mc I, Mc IV and Mc V; phalangeal articular facets on external face of shaft on Mc I–IV. Phalanges present on digits I–IV (phalangeal formula 2-1-1-1-0); spike-like ungual present on digit I. Phalanges on digits II–IV rounded and broadest transversely; Mc III phalange heavily reduced (autapomorphic). Ilium massive with broad rounded preacetabular lobe directed perpendicular to sacral axis; pneumatic cavities present within iliac blade. Pubes massive and long, fused to ischia via a shallowly sigmoidal pubic apron. Ischia with distinct iliac peduncle on a narrow shaft leading to a broad ischial blade, which is fused to the opposite ischium along the mid-line of the pelvic girdle. Femur robust with lateral bulge; intermediate robusticity (autapomorphic); distal condyles bevelled dorso-medially; shaft elliptical in cross-section and deflected medially. Tibia robust; proximal articular condyle sub-equally expanded; cnemial crest projects cranially then laterally (autapomorphic); ovoid distal articular surface; distal breadth more than twice that of mid-shaft. Fibula expanded proximally; intermediate robusticity (autapomorphic); distal articular surface bevelled cranio-medially. Astragalus quadrangular; posterior fossa undivided.

### Description

#### Axial Skeleton

The axial skeleton of the holotype (AODF 603) is known from isolated dorsal ribs, isolated gastralia and two isolated sacral processes.

#### Dorsal Ribs (Figure 3)

Ten dorsal ribs have been recovered, all with damaged or broken proximal and distal ends. A mid-dorsal rib and posterior dorsal rib preserve the expanded region of the proximal end and well preserved distal ends. The proximal expansion possesses an excavated region and pneumatic cavities. Both ribs possess camellate internal bone structure in the proximal expansion only. The cross section of the mid-dorsal rib is rectangular, plank-like and slightly bowed caudally. It is broad along its length with an expanded distal end, which is rounded and rugose in distal aspect. The posterior dorsal rib is sub-triangular in cross-section, bowed along its length, and narrow. Measurements in Table S1.

**Figure 3:**
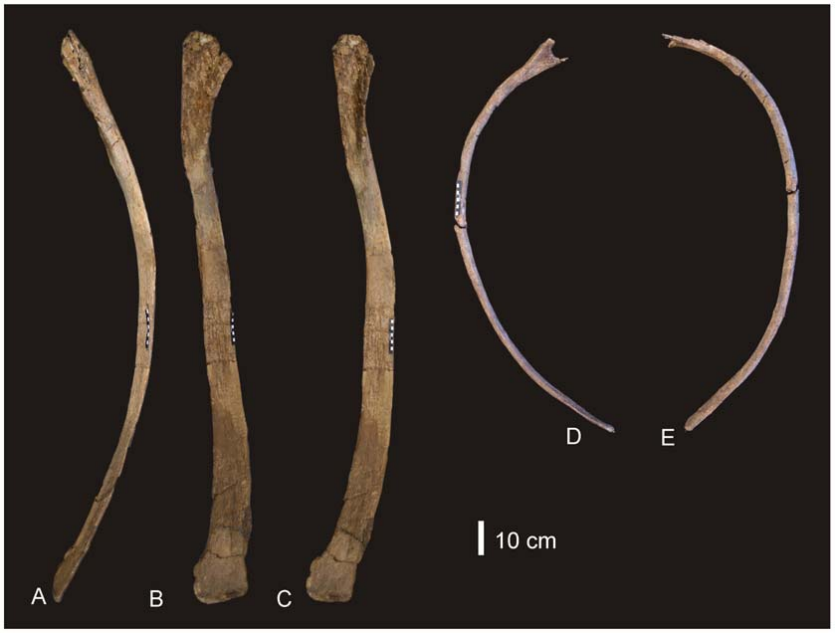
Dorsal ribs of *Diamantinasaurus matildae*. Mid-dorsal rib in lateral (A), anterior (B) and posterior (C) views. Posterior dorsal rib in posterior (A) and anterior (B) views.

### Appendicular Skeleton

#### Pectoral Girdle

The pectoral girdle of *Diamantinasaurus matildae* is represented in the Holotype AODF 603 by a right scapula and left sternal plate.

#### Right Scapula (Figure 4A)

The scapular blade has a flat lateral surface and a shallowly concave medial surface. In cross section the scapular blade is thin medio-laterally and rectangular in shape, with tapered cranial and caudal margins. The proximal end of the scapular blade is expanded into a broad cranio-dorsal blade (acromion blade) and a thick and triangular glenoid fossa, bevelled medially. Midline scapular blade ridge absent. The ventral edge of the scapular blade is straight, possessing a proximo-ventral process which projects medio-ventrally and is distal to an expanded scapular glenoid. The acromial process is a broad basin with an inconspicuous acromial ridge. Between the scapular blade and the acromial process a section of bone was damaged post-mortem and is made up of several small randomly oriented bone fragments, displaced from their original position. It is likely that this damage was caused by displacement of surface bone via bioturbation. There is no indication of pathologies, green fractures or crushing of the specimen. The acromion process intersects at approximately 90° to the scapular blade. The coracoid articulation is thick and sub-triangular in cross-section. Scapular blade forming an approximate 45° angle with the coracoid articulation. Both the scapular glenoid and coracoid articulation are medio-laterally expanded to form a thick fossa and thick attachment for the coracoid. Measurements in Table S2.

**Figure 4:**
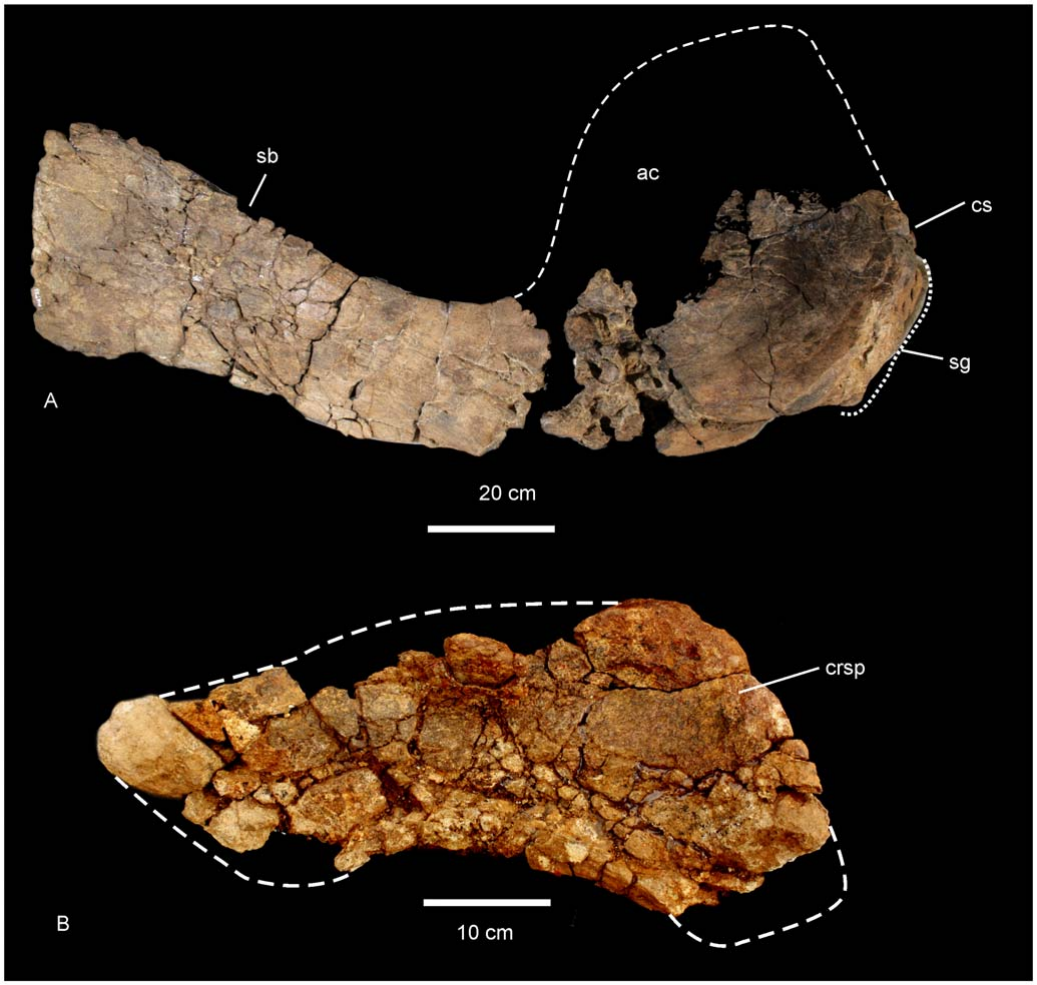
Scapula and sternal plate of *Diamantinasaurus matildae*. Right scapula in lateral view (A). Left sternal plate in ventral view (B). *Abbreviations*: *ac*, acromial blade; *crsp*, cranial ridge of sternal plate; *cs*, coracoid suture; *sb*, scapular blade; *sg*, scapula glenoid. Dashed line indicates suggested area missing from specimen.

#### Left Sternal Plate (Figure 4B)

The bone is badly preserved, having been cracked up and fragmented post deposition. However, there is enough preserved of the element to determine the margins of the bone. In ventral view, the sternal plate is a crescentic flat bone with a cranio-ventral ridge. The cranial margin of the plate is thick and rounded, being broader than the caudal margin which is distinctly rounded at a medio-caudal apex. The outline of the medial edge is convex, whilst the lateral edge is concave. Measurements in Table S3.

#### Forelimb

All of the forelimb elements of *Diamantinasaurus matildae* are represented in the Holotype AODF 603, except for the radius.

#### Right and Left Humerus (Figure 5)

Both right and left humeri are present in the holotype specimen (AODF 603). Both elements are well preserved with only the proximo-lateral margin of the right humerus missing. The following description is based on the best preserved regions of both humeri. Humerus stout. Based on the ratio of mid-shaft width to humeral length, the humerus is intermediate in robusticity with a ratio of 0.205, falling between the ratio considered to be gracile (<0.15) or robust (>0.25) [15]. Lateral margin of the diaphysis concave. Delto-pectoral crest (dpc) prominent, projecting cranio-laterally and extending distally to the mid-shaft. The distal end of the dpc is markedly expanded medio-laterally. Proximal humeral head projects above level of proximal margin of the dpc, forming a shallowly sigmoidal proximal border. Proximal end broad with proximo-lateral corner square. Proximal head round and broader than distal epiphysis. Distal end flat with merged condyles. The radial condyle is more transversely expanded than the ulnar condyle. The ulnar condyle is offset distally to the distal margin of the radial condyle. Distal end broadly rectangular with distinct rugosities. Distal condyles only slightly developed onto the anterior face of the shaft. Mid-shaft cross-section sub-triangular. Humerus proximal breadth approximates 50% of its length. Humerus/Femur Length ratio is 0.81. Measurements in Table S4.

**Figure 5:**
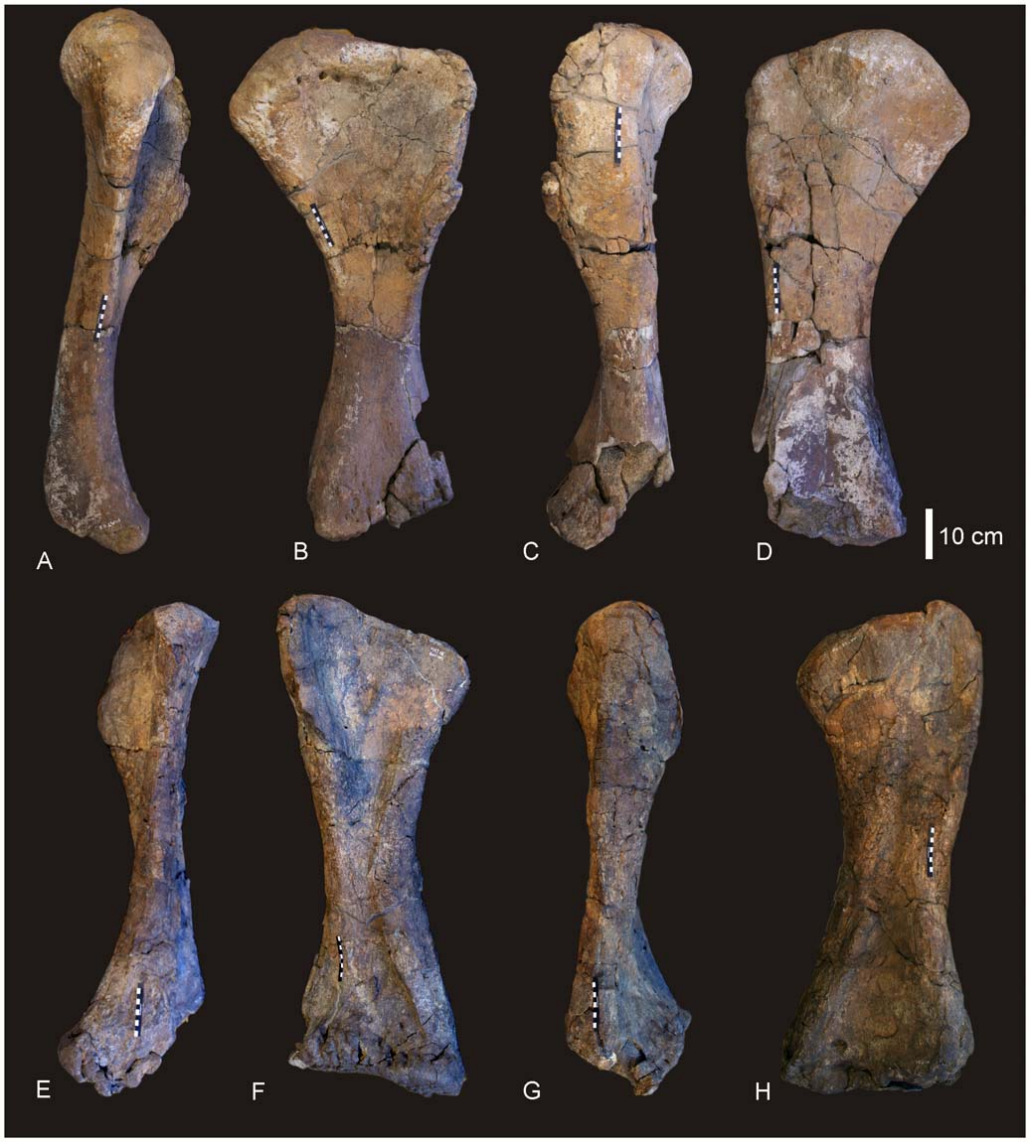
Humeri of *Diamantinasaurus matildae.* Left humerus in medial (A), anterior (B), lateral (C) and posterior (D). Right humerus in lateral (E), anterior (F), medial (G) and posterior (H).

#### Ulna (Figure 6)

A right ulna is exceptionally well preserved and complete. The ulna is stout with a massively broad proximal end. The ratio of maximum proximal width to length is 0.549, making the ulna extremely stout [15]. However, the ratio of mid-shaft width to length is 0.18, making the ulna gracile [15]. The proximal articulation is tri-radiate. The medial and posterior processes dominate the tri-radiate processes, with the cranio-medial process being longest. The cranio-medial process possesses a broad articulation, strongly concave in lateral view. Olecranon distinct and rounded, projecting above proximal articulation. The distal articular end is sub-triangular with each apex rounded. Cranio-medial process forms a crest along the diaphysis, which runs the entire length of the ulna, terminating at the distal margin. Measurements in Table S5.

**Figure 6:**
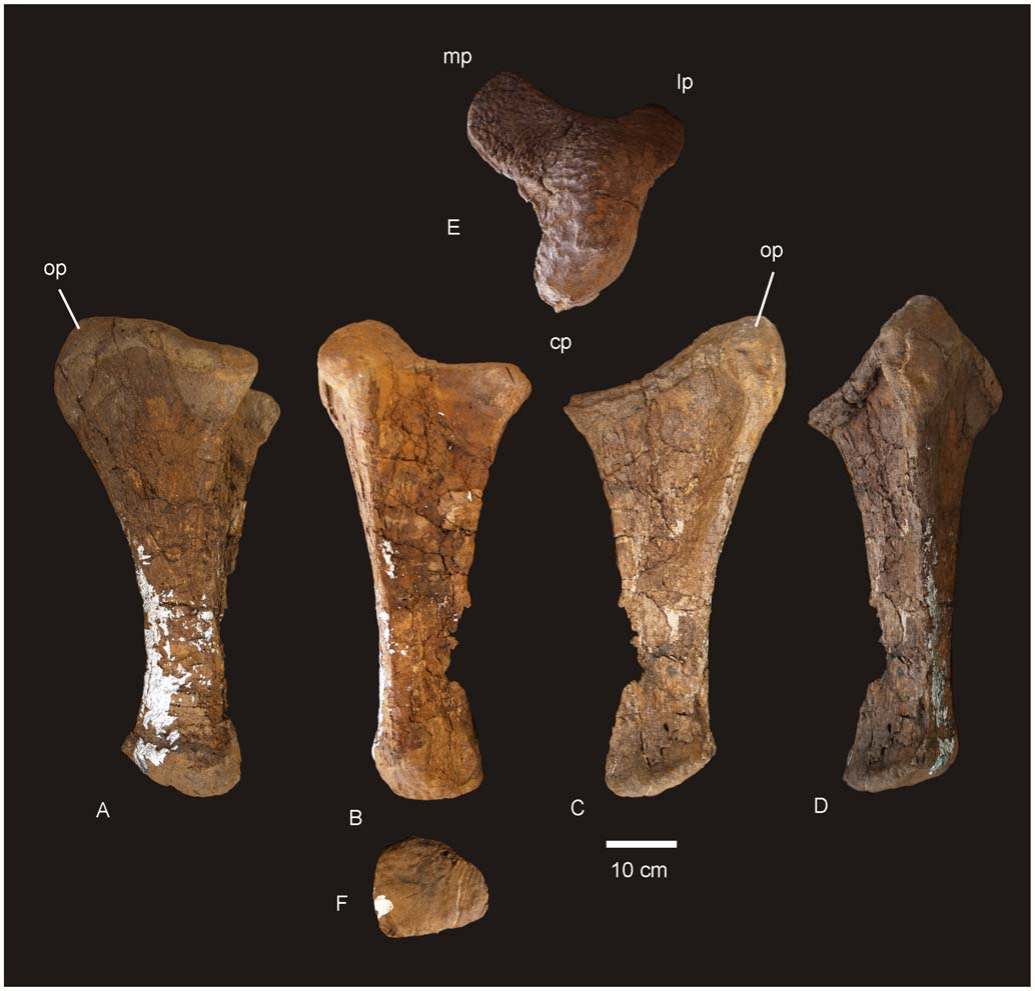
Ulna of *Diamantinasaurus matildae.* Right ulna in lateral (A), anterior (B), medial (C), posterior (D), proximal (E) and distal (F) views. *Abbreviations: cp*, caudal process; *lp*, lateral process; *mp*, medial process; *op*, olecranon process.

#### Manus (Figure 7)

The manus of AODF 603 is robust and proportionately long relative to the length of the ulna (Mc III 65% of the length of the ulna). The phalangeal count for the manus is 2-1-1-1-0, where Mc I possesses a phalange and ungual, whilst Mc II–IV possess a single terminal phalange each. Mc V is lacking any associated phalange and does not posses a phalangeal articular facet. The metacarpals articulate closely to one another in a tight proximal semi-circle, leaving only a small proximal internal gap. In distal view the metacarpals form a more open semi-circle with a slightly splayed external margin, which then connect to the small rounded button-like phalanges. Measurements in Table S6.

**Figure 7:**
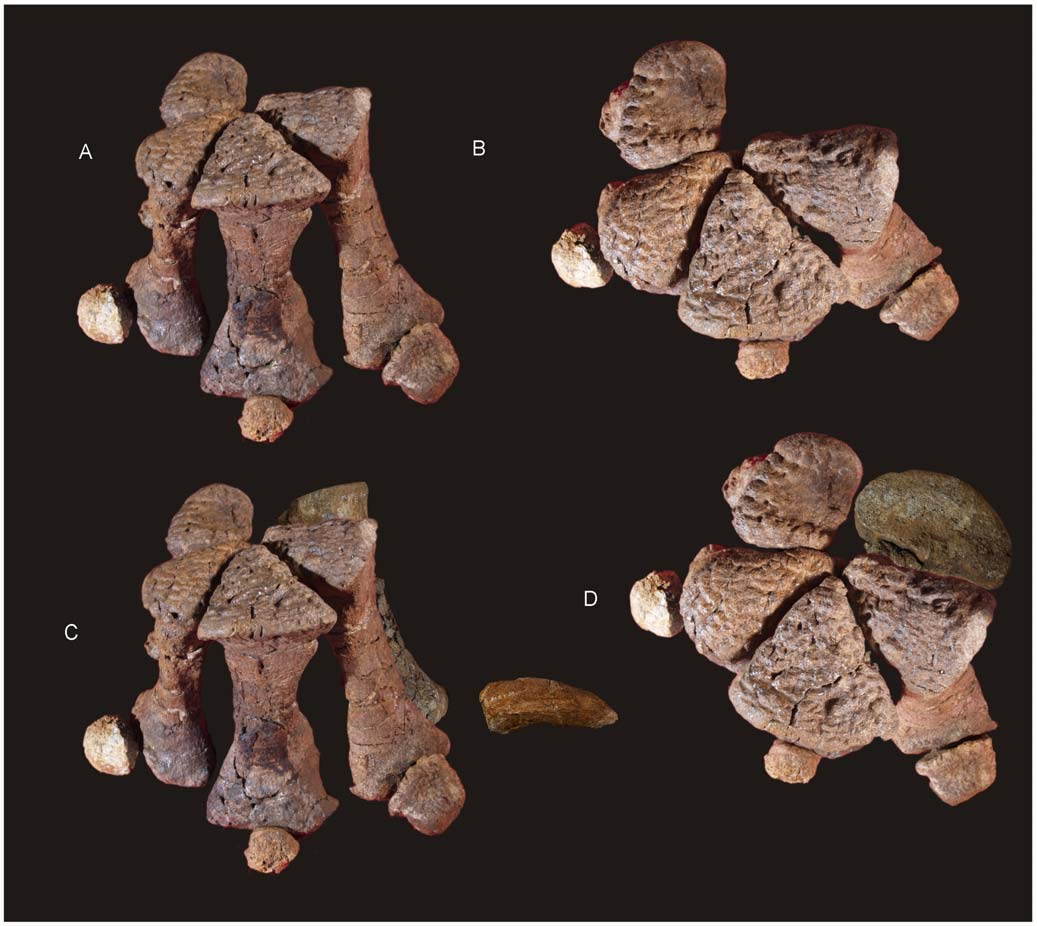
Manus reconstruction of *Diamantinasaurus matildae.* Preserved right manus in antero-external view (A) and proximal (B) views. Right manus reconstruction using left Mc I (reversed) and ungual in antero-external (C) and proximal (D) views.

### Metacarpus (Figures 7 & 8)

#### Metacarpals

Metacarpal (Mc) III is the longest metacarpal in the manus, followed by Mc II, Mc I, Mc IV and Mc V. All are robust metacarpals, expanded at both the proximal and distal ends, with Mc I–IV possessing phalangeal articular surfaces that extend onto the external surface of the shaft. All metacarpals bear distinct condylar rugosities on both the proximal and distal ends.

#### Left Metacarpal I

Well-preserved proximal and distal ends; mid-shaft surface bone badly preserved, broken into small pieces and slightly crushed post-mortem. The proximal articular surface is colonnade, with a convex medial and concave lateral margin. Oval in proximal view with an internal process. Broadest proximal end of all of the metacarpals. Proximal and distal ends expanded relative to shaft width. Undivided distal articular surface. A rounded tuberosity extends distally from the medial corner of the distal end. The transverse axis of the distal end is flat in side view. The external face is convex along its entire length, whilst the proximal-most third of the internal face of the shaft is concave. The mid-shaft cross-section is oval in shape. The distal articular end is subrectangular in distal view with a broader medial face.

#### Right Metacarpal II

Mc II well preserved along its entire length. Proximal end triangular with internal apex and third largest of metacarpals. Articular surface rounded and slightly raised internally. Shaft subtriangular in cross-section and slightly bowed. Distal end subrectangular in distal view with articular surface extending onto external face of shaft. Internal surface of shaft developed into a crest running from the proximal apex to the middle of the shaft. Distal articular surface broad, flat and undivided.

#### Right Metacarpal III

Mc III is the largest metacarpal in the manus and is well preserved. The proximal and distal ends are expanded relative to the mid-shaft. The proximal end is robust and triangular in proximal view with an internal apex. Articular surface flat and second largest of all metacarpals. Mid-shaft triangular in cross-section. Shaft slightly bowed (more than Mc II) with three distinct ridges originating from the apices of the proximal end, running distally to the base of the distal expansion. Distal end both broad and sub-rectangular in shape with a small extension of an articular face onto the anterior face of the shaft.

#### Right Metacarpal IV

Mc IV is well preserved, robust metacarpal with expanded proximal and distal ends. The proximal end is sub-triangular with a rounded external and constricted internal apex. The articular surface is convex. Two crests run distally from the proximal end, one originating from the internal apex and merging with the shaft just distal of the mid-shaft. The second originates just distally of the proximal margin on the lateral face of the shaft, running distally along the lateral shaft, and then curving internally to meet with the first crest just distal of the mid-shaft. The distal end is sub-hexagonal in shape, being transversely elongate and rounded externally. An articular surface extends onto the external surface of the shaft.

#### Right Metacarpal V

Mc V is the smallest metacarpal and is robust in nature, with the mid-shaft width to metacarpal length ratio greater than 0.10 (AODF 603 Mc V = 0.25) [15]. The proximal and distal ends are similarly expanded with the proximal end being square in shape and possessing a distinct external tuberosity that extends proximally from the otherwise flat articular surface. The distal end is semi-circular in distal view with a short lateral process which juts out from the articular surface. The distal end is transversely expanded and externo-internally compressed, with a concave internal face. The external margin is concave and does not possess the distinct articular surface present in Mc I–IV, either on the distal face or extending onto the external surface of the shaft.

#### Phalanges

Three phalanges and one ungual have been recovered. The three phalanges were found in association with the right Mc II–V manus, whilst the ungual was found separate to these. Based on the close association of the phalanges to the metacarpals it was possible to match each phalange to their respective metacarpal.

Mc II preserves the largest phalangeal articular face and connects well with the largest phalange. Mc IV preserves the second-largest phalangeal articular surface and articulates well with the second-largest phalange. The third largest phalangeal articular surface was present on the left Mc I; however, no corresponding phalange was recovered for this position. The smallest articular surface was present on Mc III and articulated well with the heavily reduced and smallest phalange. Each of the phalanges is rounded cranially and possesses distinctly concave articular facets on their caudal margins.

#### Ungual (Figure 8)

A single manus ungual has been recovered. It is considered here to represent a manus ungual because of its hypertrophic nature, lack of curvature and lack of distinctly weight-bearing features. The ungual is a relatively straight spike possessing a slight curvature along its long axis and a very prominent distal point. The ungual is relatively symmetrical along the long axis. In cross-section the ungual is tapered both dorsally and ventrally, whilst also being medio-laterally compressed. A narrow proximal articular surface suggests its connection to a relatively small and basic phalange. The lack of a defined proximal facet indicates that this ungual was not tightly articulated to the preceding phalange.

**Figure 8:**
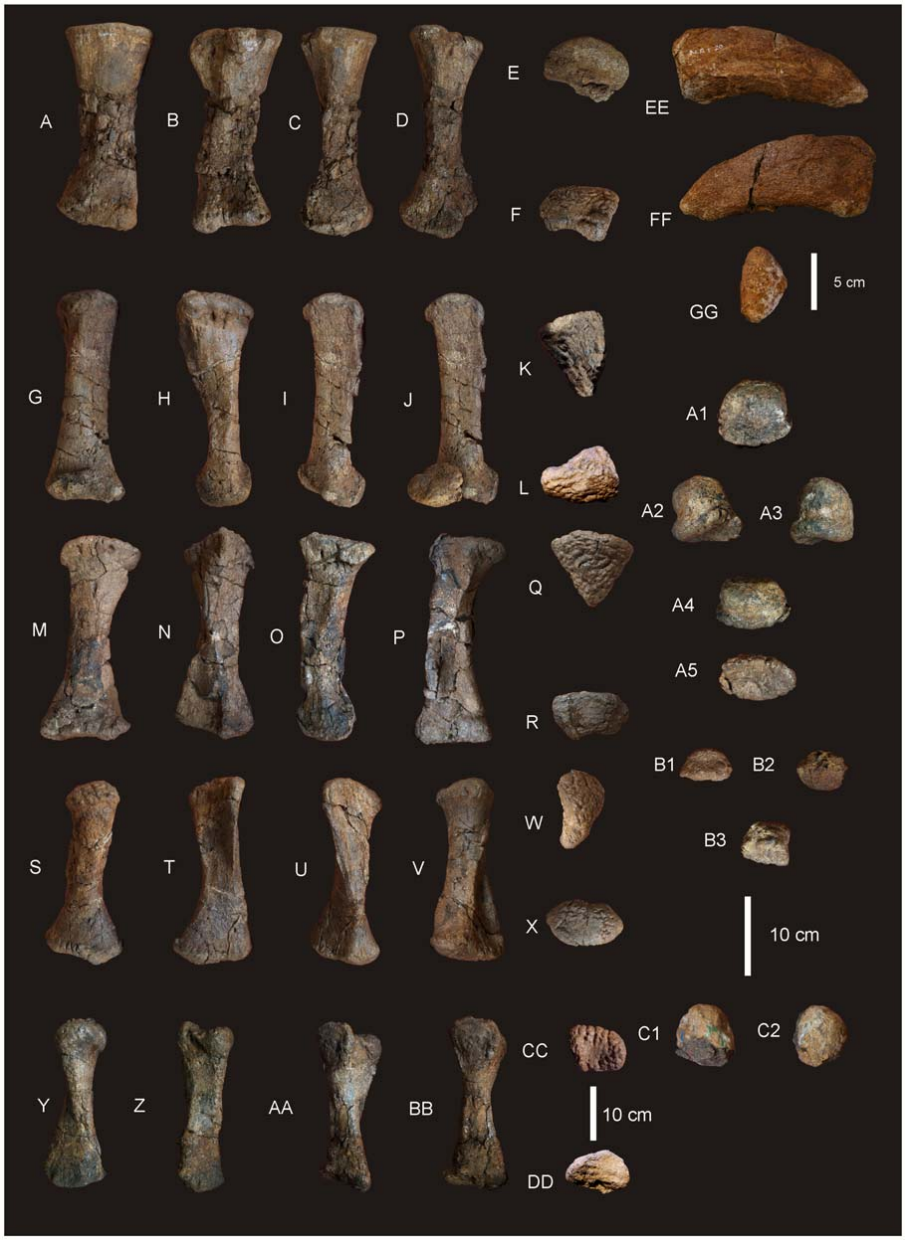
Metacarpals and phalanges of *Diamantinasaurus matildae.* Left Metacarpal (Mc) I in external (A), internal (B), medial (C), lateral (D), proximal (E) and distal (F) views. Right Mc II in external (G), internal (H), medial (I), lateral with phalange (J), proximal (K) and distal (L) views. Right Mc III in external (M), internal (N), medial (O), lateral (P), proximal (Q) and distal (R) views. Right Mc IV in external (S), internal (T), medial (U), lateral (V), proximal (W) and distal (X) views. Right Mc V in external (Y), internal (Z), medial (AA), lateral (BB), proximal (CC) and distal (DD) views. Manus ungual in anterior (EE), posterior (FF) and proximal (GG) views. Mc II.1 phalange in dorsal (A1), medial (A2), lateral (A3), distal (A4) and proximal (A5) views. Mc III.1 phalange in ventral (B1), dorsal (B2), proximal (B3) views. Mc IV.1 phalange in dorsal (C1) and distal (C2) views.

These features are not usually seen in sauropod pedal unguals, instead they are generally broader and more robust in all dimensions; much more recurved and asymmetrical; possess broad and distinct proximal articular facets; and do not taper at both the dorsal and ventral margins. In addition, the presence of an articular surface on the left Mc I suggests that the first digit had at least one phalange, which may have connected to a manus ungual. As yet, no metatarsals, pedal phalanges or pedal unguals have been recovered from the type specimen locality.

#### Pelvic Girdle (Figure 9)

The pelvis of AODF 603 preserves the left ilium, right and left pubes, right and left ischia and two lateral processes from the sacrum. The left ilium is an isolated element and has broken away from the sacrum and right ilium post-mortem. The articular section of the pubic peduncle broke away from the main body of the ilium post-mortem and was found close by in the deposit. The dorsal rim, internal connection to the sacrum and the caudal margin of the ilium have lost surface bone, however, the main body of the ilium is well preserved, including the articular tuberosities present on the peduncles and the preacetabular lobe. The internal structure of the iliac blade can be seen from the medial and caudal faces, illustrating that the internal bone structure was made up of large camerate pneumatic vacuities. This internal structure is also found in the isolated transverse processes of the sacrum.

**Figure 9:**
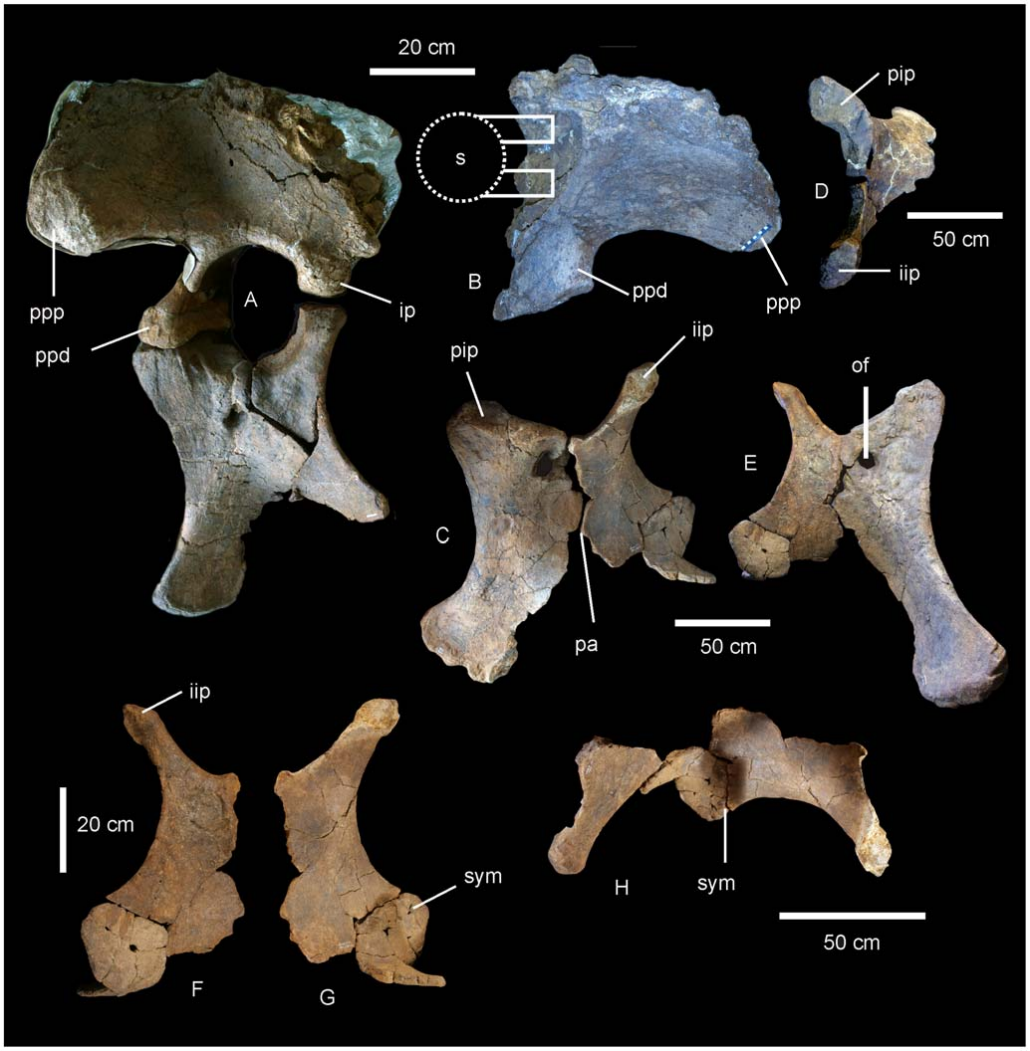
Pelvis of *Diamantinasaurus matildae.* Left reconstructed pelvis in lateral (A) view. Left ilium in anterior view (B) showing the position of the sacral vertebrae. Right pubis and ischium in medial (C), proximal (D) and lateral (E) views. Right ischium in lateral (F) and medial (G) views. Reconstructed right and left ischia in dorsal view. *Abbreviations: ip*, ischial peduncle; *iip*, iliac peduncle of ischium; *of*, obturator foramen; *pa*, pubio-ischial contact; *pip*, iliac peduncle of pubis; *ppd*, pubic peduncle; *ppp*, preacetabular process of ilium; *s*, sacrum; *sym*, fused ischial symphysis.

The ilium is very broad, with the width between the preacetabular lobes being much broader than the maximum length of the ilium. In lateral view the preacetabular process is board and rounded, somewhat truncated as it swings toward a 90° angle from the main axis of the ilium and sacrum. The highest point of the iliac blade is centred dorsal to the pubic peduncle. The pubic peduncle is massive and contributes over half of the acetabular rim. The articular surface of the pubic peduncle projects perpendicular from the main axis of the ilium and sacrum. The ischial peduncle is low and rounded, broadening medio-laterally to accommodate the iliac peduncle of the ischium.

The pubes are long and robust with a thick dorsally oriented iliac peduncle. Pubic acetabular rim and ischial articular edge meet at an obtuse angle, where these two elements begin to fuse together. The obturator foramen is positioned distal to this. The ischial articular surface of the pubis is much longer than the contribution of the pubis to the acetabular rim. The pubis is expanded both proximally and distally relative to the mid-shaft width. The pubio-ischial contact represents approximately half of the total pubic length, forming a large apron, which is shallowly S-shaped. The ratio of ischium to pubis length is 0.62.

The ischia are robust elements; however, during post-mortem both elements suffered breakages. The two elements were fused together during life and this complex was fused to the pubic apron of their respective pubes. The ischial complex broke into four sections; the two iliac peduncles preserved sections of the ischial blade, the right preserving the most intact. A third section represents the remaining piece of the left ischium, which connects to the pubic apron, whilst the fourth piece connects the right ischial blade to the left ischial blade and preserves the fused midline symphysis.

The ischial blade is much shorter than the pubic blade, possessing a similar medial and lateral depth along its length. The blade contacts the other ischium via a central fused region, which forms a shallow basin between the two ischia. The ratio of the width across the ischial blade at its mid-length to the total length of the ischium is greater than 0.2. The pubic peduncle of the ischium is dorso-ventrally extended to encompass the entire length of the ischial blade. Iliac peduncle of ischium distinctive and well separated from the body of the ischium. Measurements in Table S7.

### Hind limb

#### Femur (Figure 10A–C)

A complete right femur is preserved in the holotype (AODF 603). The only post-depositional deformation to occur to this heavy element was a crack that has formed from the proximo-lateral corner across the caudal face of the shaft toward the distal condyles. Associated with this crack, the lateral margin has rotated caudally so that the lateral bulge faces caudally rather than laterally. The femur is intermediate in robustness ratio ( = 0.196) being close to the minimum ratio for robust (>0.20) and above the ratio considered gracile (≤0.10) [15]. In caudal view the femoral head is massive and rounded, located dorsal to the greater trochanter, which is also massive and rounded, however, constricted cranio-caudally. The fourth trochanter is reduced to a low ridge, approximately 150 mm long. Distal to the greater trochanter, a lateral bulge is well developed. In original position, this bulge would have projected laterally, forming a greater medio-lateral width across the femur at this point than what is preserved in the specimen. The transverse (medio-lateral) length of the femur mid-shaft is approximately twice that of the cranio-caudal length of the mid-shaft (262 mm∶120 mm), forming an elliptical cross-section. When rested on the distal condyles, the shaft is deflected medially. The distal condyles are robust and cranio-caudally extended being sub-equal in transverse width and bevelled dorso-medially and extend just on to the disto-cranial margin of the shaft. The epicondyle is well developed laterally and oriented caudally. The medial condyle is cranio-caudally longer than the lateral condyle. Measurements in Table S8.

**Figure 10:**
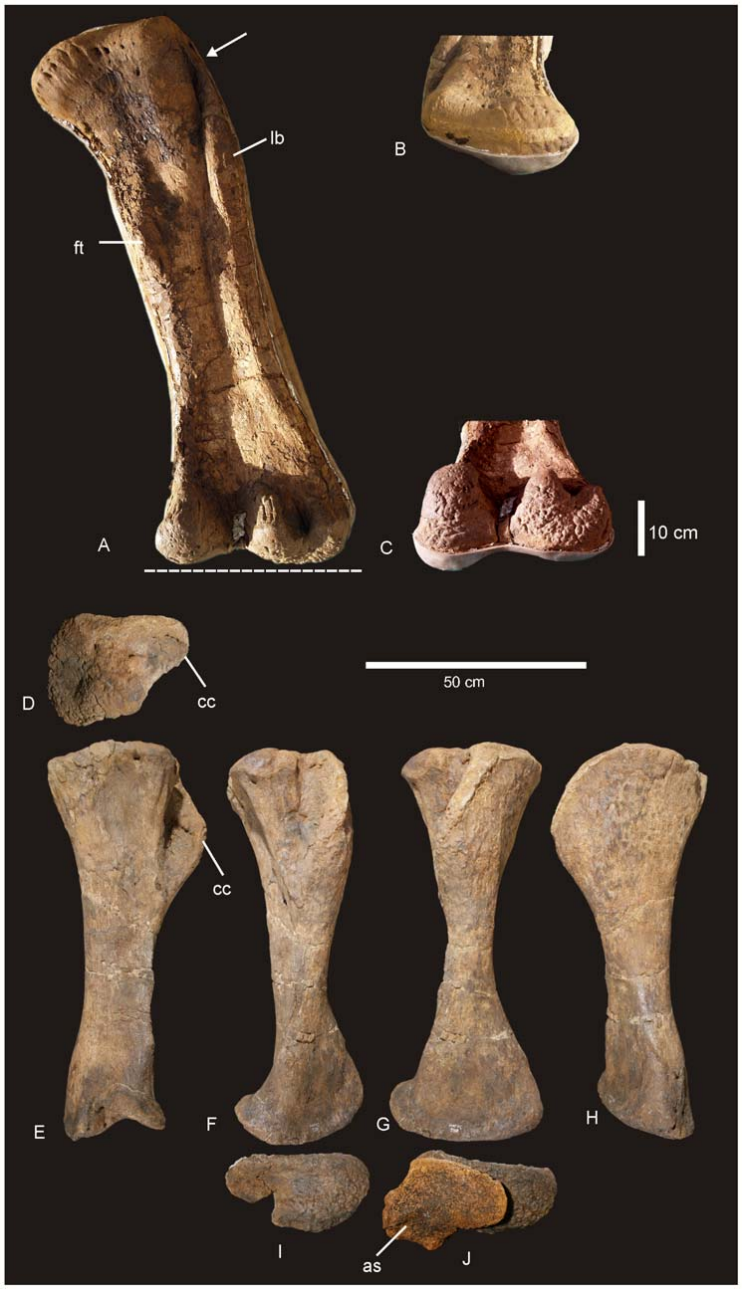
Femur of *Diamantinasaurus matildae.* Right femur in posterior (A), proximal (B) and distal (C) views. Arrow indicates proximal post-mortem crack running distally. Dashed line indicates horizontal plane on which the distal condyles are oriented. Tibia of *Diamantinasaurus matildae.* Right tibia in proximal (D), lateral (E), anterior (F), medial (G), posterior (H), distal (I) views, and articulated with astragalus in distal view (J). *Abbreviations: as*, astragalus; *cc*, cnemial crest; *ft* fourth trochanter; *lb*, lateral bulge.

#### Tibia (Figure 10D–J)

The right tibia is known from the holotype (AODF 603) and is the best preserved element of the skeleton. The element is robust with the proximal end expanded equally cranio-caudally and medio-laterally; the distal end is expanded medio-laterally and compressed cranio-caudally. The proximal condyle is sub-circular in shape with a flat articular surface. The cnemial crest is thick and robust, projecting cranially at its proximal margin, then scooping distally so that the distal margin of the crest projects laterally and encloses a deep fossa. The shaft of the tibia is relatively straight and possesses a twist in the distal margin. The distal end is broader medio-laterally than cranio-caudally creating an ovoid distal profile with a distinct lateral notch. The breadth of the distal end is more than 200% of the mid-shaft breadth. Measurements in Table S9.

#### Fibula (Figure 11)

The right fibula is known from the holotype (AODF 603) and is broken in three places. The robustness of the fibula, as determined by the mid-shaft width: total length of the fibula, is intermediate ( = 0.187) between that considered to be robust (>0.25) and gracile (≤0.15) [15]. The proximal and distal ends are expanded relative to the mid-shaft, with the proximal end expanded into an ovoid articular end which is rounded (convex) proximally and narrowed cranially. The lateral trochanter is present and elongate along the caudo-lateral margin of the shaft. The distal end is rounded and sub-triangular in distal profile. The distal articular surface is bevelled cranio-medially so that the cranial margin of the articular surface is dorsal to the caudal surface.

**Figure 11:**
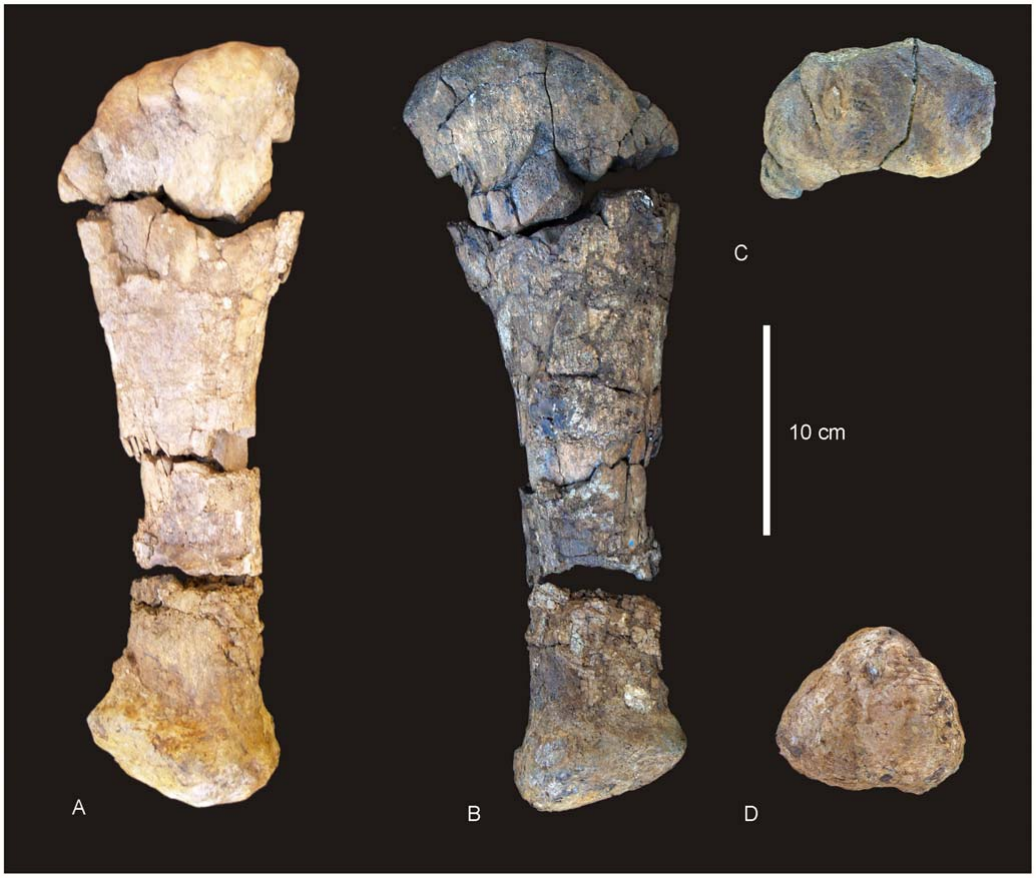
Fibula of *Diamantinasaurus matildae.* Right fibula in lateral (A), medial (B), proximal (C) and distal (D) views.

#### Astragalus (Figure 10J & 12)

The right astragalus is preserved in the holotype (AODF 603) and is complete; wedge-shaped with a reduced cranio-medial corner. In proximal view the astragalus is sub-equal in cranio-caudal length to medio-lateral width, forming a rounded quadrangular shape. The proximal articular surface divided two regions; a lateral ascending process, which is developed into a broadly rectangular block shape with a flat dorsal surface; and a medial process, which is produced into a shallow fossa for the tibial articular surface. In medial view, the tibial articular surface is concave leading laterally to a deep and undivided posterior articular fossa. The medial margin of the astragalus is thick and heavily rugose. In lateral view, the articular surface for the fibula is preserved along with a distinct ridge which runs from the cranial margin to the caudal margin where it connects to a caudally developed facet.

**Figure 12:**
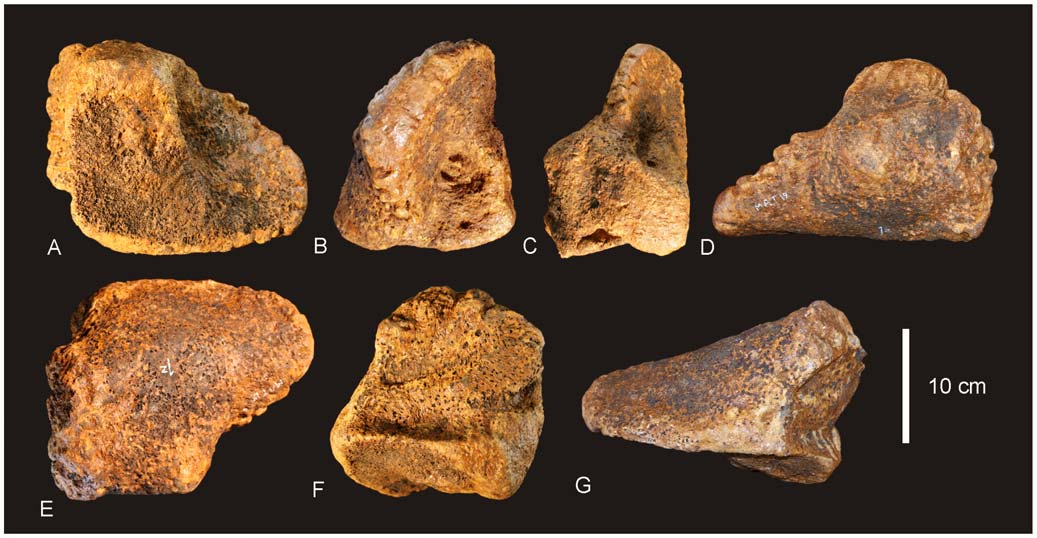
Astragalus of *Diamantinasaurus matildae.* Right astragalus in proximal (A), medial (B), postero-medial (C), posterior (D), distal (E), lateral (F) and antero-lateral (G).

Sauropodomorpha, Huene, 1932
Titanosauriformes, Salgado et al., 1997
*Incertae sedis*

*Wintonotitan* gen. nov.

urn:lsid:zoobank.org:act:40D8C5E4-BC06-4AD7-A740-3755892A2E57

#### Etymology

*Winton*, for the town of Winton. *Titan* –Giant in Greek Mythology.

#### Type species

*Wintonotitan wattsi*

*Wintonotitan wattsi* sp. nov.

urn:lsid:zoobank.org:act:8C6C2C13-CA79-470E-AC81-54BA5BF0B4A2

#### Synonymy

*Austrosaurus* sp. [20]

#### Etymology

For Keith Watts, who discovered the type specimen and donated it to the Queensland Museum in 1974.

#### Holotype

QMF 7292; left scapula, partial left and right humeri, partial left and right ulnae, partial right and near complete left radii, near complete right metacarpus preserving complete metacarpals II–V with proximal half of metacarpal I, fragmentary dorsal and sacral vertebrae and ribs, partial right ilium, right ischium, caudal vertebral series including anterior caudals, middle caudals, posterior caudals and proximal chevrons. Numerous additional unidentified or unrecognised bone fragments (Figure 2C).

#### Referred specimen

QMF 10916; Chorregan, Winton; isolated middle and posterior caudals.

#### Type Locality

QML 313 “Triangle Paddock”, Elderslie Station, approximately 60 km north-west of Winton, central Queensland, Australia.

#### Horizon & Age

Winton Formation, latest Albian (Cretaceous).

#### Diagnosis

*Wintonotitan wattsi* gen. et sp. nov. is characterised by the unique association of the following features. Dorsal vertebrae possessing camellate pneumatic cavities; deep eye-shaped pleurocoels; prespinal and postspinal laminae extending along entire length of neural spine; incipient spinoprezygopophyseal lamina (autapomorphic). Dorsal ribs with pneumatic cavities along 1/3 of proximal end; large and plank-like. Caudal vertebrae number approximately 35; all with solid internal bone structure; anterior and middle caudal vertebrae amphiplatyan; anterior caudals with straight neural spines above cranial half of vertebra; posterior caudals incipiently biconvex and cylindrical (autapomorphic). Scapula long with broad acromial blade; ventromedial process present; mid-scapular blade ridge present; scapular-coracoid articular connection thin medio-laterally; distinct acromial ridge. Humerus gracile with divided distal condyles; deltopectoral crest low and narrow, terminating proximal of mid-shaft. Ulna long and gracile with broad proximal end and rounded distal articular end. Radii long and gracile; expanded sub-equally at proximal and distal ends. Metacarpals long and robust, distal condyles undivided and flat, missing phalangeal articular facets. Mc I longest, then Mc II, Mc III, Mc IV and Mc V. Preacetabular blade of ilium projecting antero-laterally from sacral axis; iliac blade with pneumatic cavities. Ischium broad and flat with robust iliac peduncle and thin ischial blade with rounded distal margin.

### Description

### Axial Skeleton

#### Dorsal Vertebrae (Figure 13A–C)

Fragmentary isolated remains of dorsal vertebral centra, neural spines and transverse processes are preserved in the holotype (QMF 7292). All of these fragments preserve distinctively complex camellate internal pneumatic structures. A single large dorsal centrum is the best preserved portion of a dorsal vertebral body. It is missing the rounded cranial articular surface and neural arch and spine. It possesses somphospondylus internal bone texture; the posterior concave articular surface; and the posterior margin of the left caudally acuminate, pleurocoel. The only lamina preserved in this dorsal is the origin for the posterior centrodiaphophyseal lamina, which is angled cranially. The lateral and ventral margins of the centrum are rounded following the elliptical shape of the articular face.

**Figure 13:**
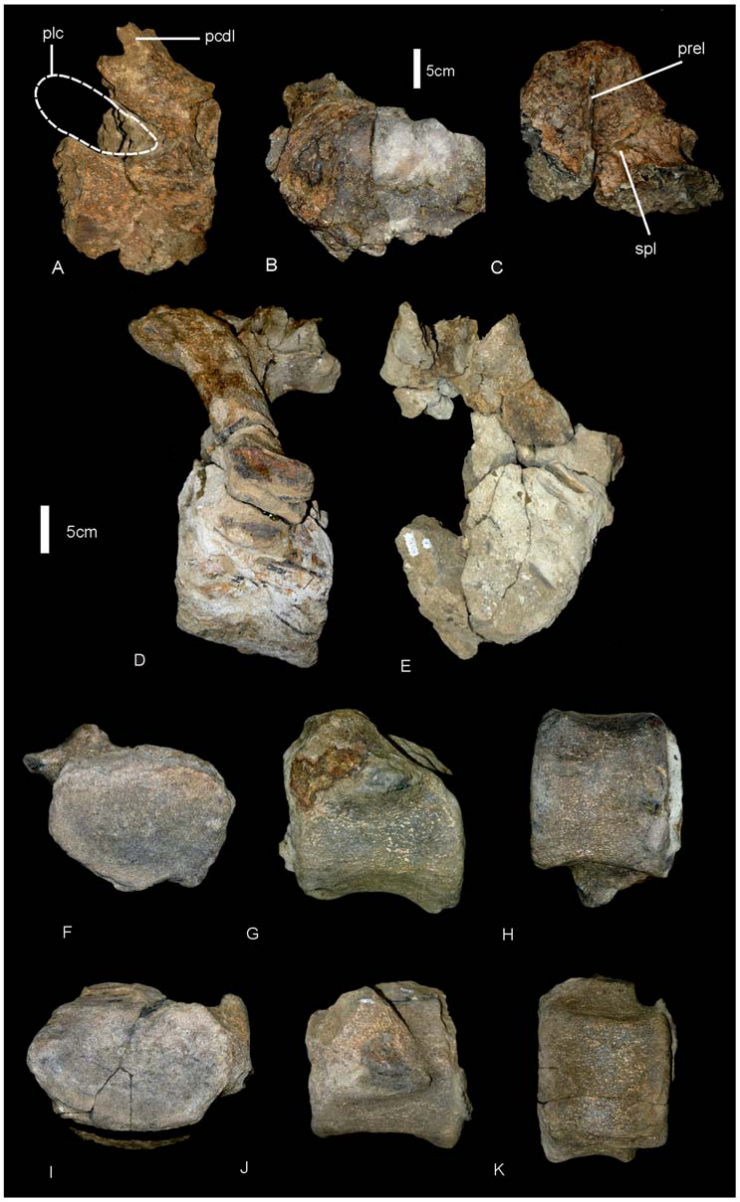
Dorsal vertebrae of *Wintonotitan wattsi.* Partial dorsal centrum in lateral (A) and posterior (B) views. Isolated neural spine in anterior view (C). Anterior caudal vertebrae of *Wintonotitan wattsi.* Anterior caudal vertebra in lateral (D) and anterior (E) views. Anterior caudal vertebra in posterior (F), lateral (G) and ventral (H) views. Anterior caudal vertebra in anterior (I), lateral (J) and ventral (K) views. *Abbreviations: plc*, pleurocoel; *pcdl*, posterior centrodiapophyseal lamina; *prel*, prespinal lamina; *spl*, spino-prezygopophyseal lamina.

The dorsal margins of four isolated neural spines are preserved with rounded quadrangular dorsal surfaces. The neural spines are relatively stout with broad triangular bases, probably less than 20% of the total vertebral height. Each possesses complex camellate internal pneumatic structure and simplified prespinal and postspinal laminae. A ridge extends from the base of the prespinal lamina across the lateral face of the neural spine toward the expansion for the prezygopophysis. This is interpreted as an incipient spinoprezygopophyseal lamina. Measurements in Table S11.

#### Dorsal Ribs

Several large sections of dorsal ribs are preserved, all being long, broad and plank-like. Anteriorly the ribs are expanded and in cross-section possess large pneumatic vacuities internal structures. Based on the overall size of the rib fragments, the pneumatic internal structures extend as much as a third of the length of the proximal length of the rib.

### Sacral Vertebra (Figure 19D–E)

A single portion of the sacrum is preserved, including the co-ossification of two sacral vertebrae. The dorsal portion of the centra, neural arch and neural spines are missing. Only one of the vertebrae has a near complete centrum, with a posterior surface that is shallowly concave. The internal pneumatic structures of the sacral vertebrae are not as complex as those of the dorsal vertebrae, being more camerate than camellate [16∶346]. Total number of sacral vertebrae unknown. The ventral margin of the sacral vertebra possesses a rounded ventral margin with shallow lateral constrictions along the centrum body. Articular surfaces of sacral vertebrae are shallowly concave (platycoelous). Deep lateral pneumatic fossae are absent from the sides of the centra. The location of pneumatic pore that leads into central pneumatic cavities is unknown. Measurements in Table S11.

#### Caudal Vertebrae (Figure 13D–K, 14)

Twenty-nine caudal vertebrae are recognised, including nine anterior, seven middle and thirteen posterior caudals. Several posterior caudals are missing and we estimate the total number of caudals to be approximately thirty-five. All caudals possess solid internal structure, with no pneumaticity. The caudal series maintains its relative centrum length over the first twenty or so caudals, not doubling in length. The anterior-most caudal centra are as tall as long, or slightly taller than long. Middle caudals are slightly longer than tall, whilst posterior caudals are longer than tall, as much as twice as long as tall. Anterior caudal centra are approximately as tall as wide, middle caudals slightly wider than tall and posterior caudals are taller than wide. Anterior caudal cranial articular faces are shallowly concave and circular to ‘heart’ shaped in anterior view. The caudal articular face is flatter than the cranial face and circular in posterior view. Middle caudals are shallowly concave on both the cranial and caudal articular faces with circular to quadrangular articular faces in anterior and posterior views. Posterior caudals possess unique cranial and caudal articular surfaces, being convex on the outer margin, then concave within the inner margin of the central articular surface (Table 2). Although in lateral view the caudal vertebrae possess distinctly biconvex articular ends, the presence of an inner concavity suggests that *Wintonotitan wattsi* possesses the unique characteristic of incipient biconvexity, an intermediate condition between platyan centra to completely biconvex centra. Posterior caudal articular faces are circular to subtriangular in anterior and posterior views. The presence of this trait in isolated posterior caudals from other localities, such as QMF10916, excludes the holotype (QMF 7292) from representing an abnormal individual.

**Figure 14:**
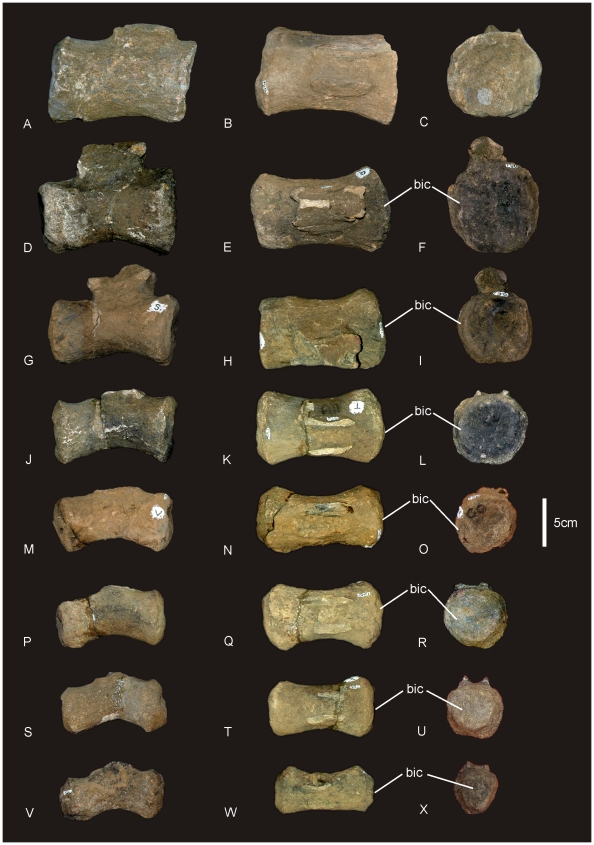
Middle and posterior caudal vertebrae of *Wintonotitan wattsi.* Middle caudal vertebra in lateral (A), dorsal (B) and anterior (C). Posterior caudal vertebrae in lateral (D, G, H, J, M, P, S, V), dorsal (E, H, K, N, Q, T, W) and anterior (F, I, L, O, R, U, X) views. *Abbreviations: bic*, incipient biconvexity.

**Table 2:** Cranial and caudal face shape of anterior, middle and posterior caudal vertebrae of QMF 7292.

Caudal Vertebra	Anterior-most	Anterior	Middle	Posterior
Cranial Face	Flat	Moderately concave	Moderately concave	Incipient convexity
Caudal Face	?	Moderately Flat	Moderately concave	Incipient convexity

Neural arches of all caudals are placed cranially over the cranial half of the caudal centrum. One anterior caudal preserves the neural arch, pre- and postzygopophysis, transverse processes and the base of the neural spine. The neural arch slopes cranially of the cranial face of the centrum, with prezygopophyses placed cranially of the centrum. The neural spine is straight. The ventral margin of the anterior caudals is shallowly concave as a groove bordered by two lateral ridges. The lateral and ventral margins combined form a quadrangular profile.

Nine anterior caudals preserve transverse processes, which are all triangular in lateral view, robust and short, projecting laterally. The transverse processes do not project caudally of the centrum. We estimate that there are 2–4 anterior caudals that are missing, therefore, the transverse processes on anterior caudals most likely disappear by caudal fifteen. The first caudal has not been recognised however, based on the caudal portion of the sacral vertebra; the anterior-most caudal vertebra would have had a flat caudal articular face.

The neural arch and spine preserves a spinoprezygopophyseal lamina which connects to the lateral surface of the neural spine. There is no contact between the spinoprezygopophyseal and spinopostzygopophyseal laminae. Prespinal and postspinal laminae are present. The prezygopophyses face dorsally.

Middle and distal caudals are cylindrical with a longitudinal shallow groove along the ventral face. Measurements in Table S11.

#### Chevrons (Figure 15)

Five chevrons are preserved; four near complete with one missing the majority of the distal shaft. All possess singular distal spines which are angled caudally. Proximally the chevrons are divided and V-shaped, each process with a rounded proximal articular surface. The depth of the haemal canal is less than 20% the total length of the chevron. Measurements in Table S12.

**Figure 15:**
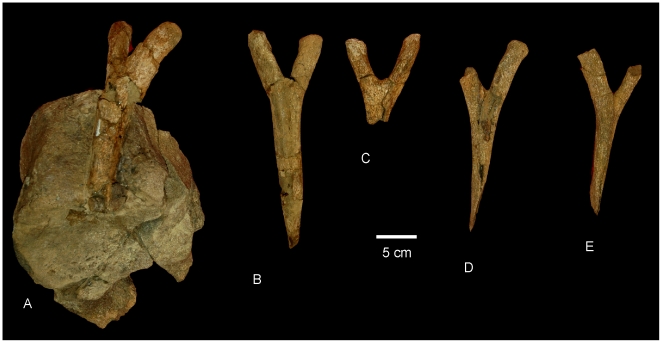
Chevrons of *Wintonotitan wattsi.* Anterior chevrons in anterior view (A–E). Chevron in (A) is attached to an anterior caudal centrum.

### Appendicular Skeleton

#### Scapula (Figure 16G,H)

A near complete scapula preserves most of the central portion of the scapular blade, acromial ridge, caudal fossa and the cranio-ventral expansion toward the glenoid fossa. The scapula does not preserve the distal margin of the scapular blade; however, this portion is preserved from the only known section of the right scapula. The scapular blade is slightly bowed laterally. The cranial fossa, coracoid suture and scapular portion of the glenoid fossa are also missing. A distinctive mid-line ridge (scapular ridge) runs the entire length of the preserved scapula, its origin at the glenoid expansion and its termination close to the distal margin of the scapular blade. This ridge is expressed in cross-section along its length as a low ‘D’ shape with shallowly concave medial face. The acromion ridge originates dorsally of the scapular ridge, intersecting it at about a 50–60°, and ventral of the cranial fossa. It forms a semi-circular ridge projecting dorsally and ending in a small dorsal expansion. A fossa is caudal of the acromion ridge and is only slightly developed, being shallow and connecting at a near perpendicular angle to the scapular blade. The glenoid fossa is not preserved; however, the cranial expansion of the scapula toward the glenoid region indicates that the glenoid was a broad semi-circular fossa which was bevelled medially. The coracoid suture is not preserved, however, the overall shape of the preserved cranial fossa indicates that the coracoid suture was most likely straight or slightly concave in shape and compressed medio-laterally.

**Figure 16:**
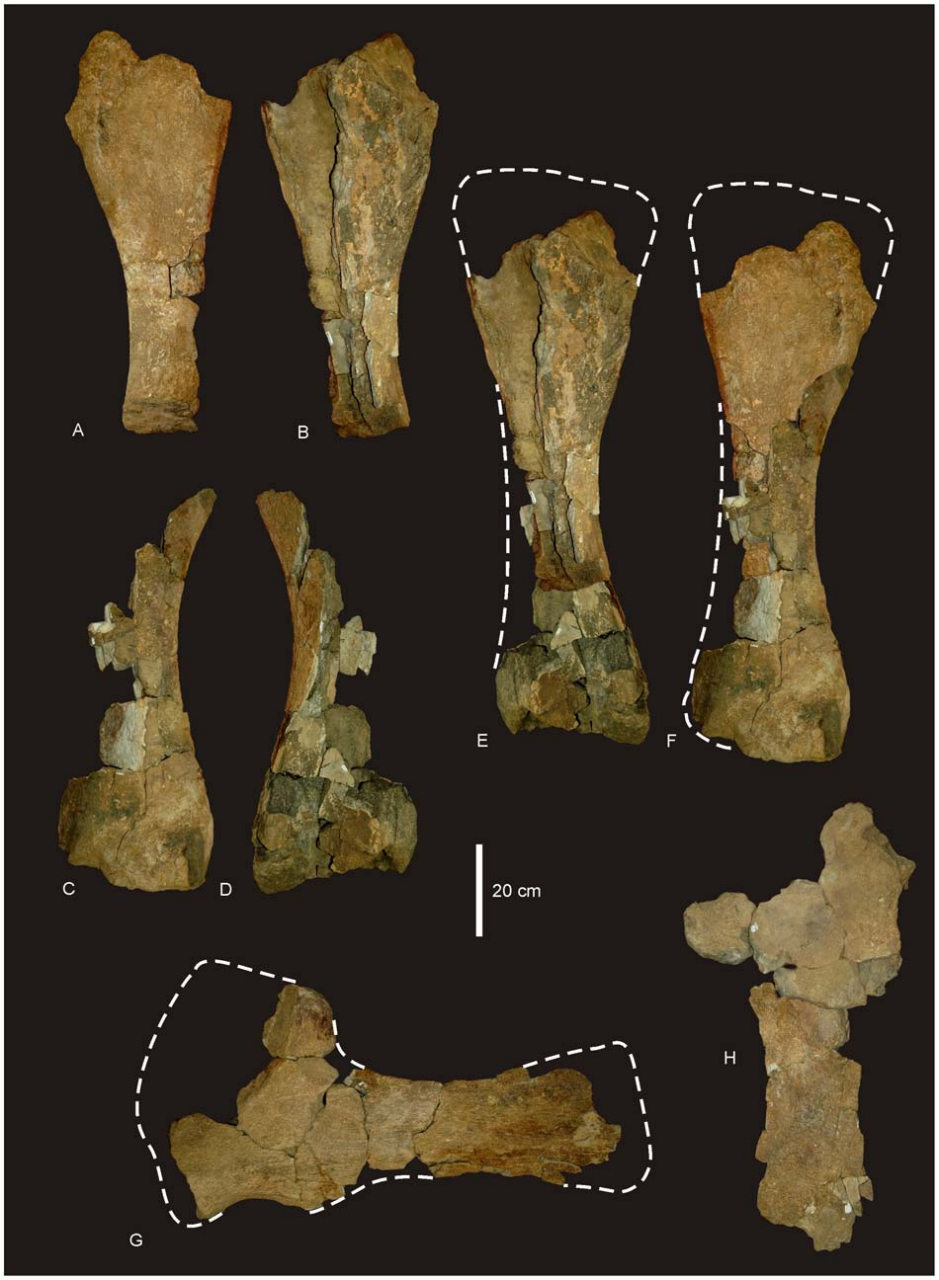
Scapula and Humeri of *Wintonotitan wattsi.* Partial left humerus in posterior (A) and anterior (B) views. Partial right humerus in posterior (C) and anterior (D) views. E. Reconstructed left humerus in anterior view by reversing (D) and aligning shaft curvature with (B). F. Reconstructed right humerus in posterior view by reversing (A) and aligning shaft curvature with (C). Left scapula in lateral (G) and medial (H) views. Dashed lines represent suggested missing areas.

The scapular blade would have been oriented at approximately 45° to the coracoid. The cranio-dorsal margin of the coracoid was most-likely very thin as the corresponding region of the cranial fossa is only 1–2 cm thick. The distal end of the scapular blade is expanded to a similar degree to that of the proximal end. Caudal of the scapular glenoid expansion, the scapular blade expands into a small tuberosity, a ventro-medial process below the scapular ridge. Measurements in Table S13.

#### Humeri (Figure 16A–F)

Two partial humeri are preserved. The left humerus preserves the proximal portion, whilst the right humerus preserves the distal portion. Both humeri are missing significant portions, including the proximal and distal articular faces. The left humerus preserves the distal origin of the deltopectoral crest, which is narrow and low. The crest is broken approximately 2/3 of its length. The right humerus preserves the origin of the distal epiphyses. In posterior aspect, the intercondylar fossa is preserved, dividing the condyles into two distinct regions, with the lateral condyle being larger than the medial condyle.

The combined proportions of both humeri indicate a long gracile element. The preserved mid-shaft width can be seen on the right humerus, which measures 195 mm wide. This measurement is taken from preserved surfaces on the medial and lateral sides of the mid-shaft. By combining the two elements together the total humerus length is estimated at 1300 mm long and within 1250–1450 mm. Using these dimensions, the humerus mid-shaft width/length ratio falls between 0.15 and 0.12, indicating that the humeral proportions are gracile compared to other sauropods.

Proximal cross-section (based on the right humerus) is semi-circular with a slightly raised lateral margin which extends into the deltopectoral crest. Cranially, the proximal end is shallowly concave. The humeral diaphysis is long and ovoid in cross-section, concave in lateral profile. The deltopectoral crest extends less than half the length of the element, with the distal origin located proximal to the middle of the shaft. Although much of the deltopectoral crest is not preserved the base of it indicates that the crest was low and narrow and not prominently projecting from the main surface of shaft. The crest itself is narrow along its length and not markedly expanded proximally or distally.

The preserved distal end of the right humerus is massive and possesses the origin of both the medial and lateral condyles, divided by the intercondylar fossa. In cross-section the lateral (ulnar) condyle is larger then the medial (radial) condyle. The distal end of the left humerus bears a post-mortem break which is covered by sediment and plant material. Measurements in Table S13.

#### Ulnae (Figure 17G–I)

The right and left ulnae are preserved in the holotype (QMF 7292), with the right ulna being the most complete element. The right ulna preserves the proximal and distal epiphyses with the proximal end preserving the proximal-most articular surface, which comprises the lateral and posterior processes. The articular surface of the medial process is missing. The left ulna is missing both the proximal and distal epiphyses, preserving the mid-shaft section and the proximal flange of the medial process.

**Figure 17:**
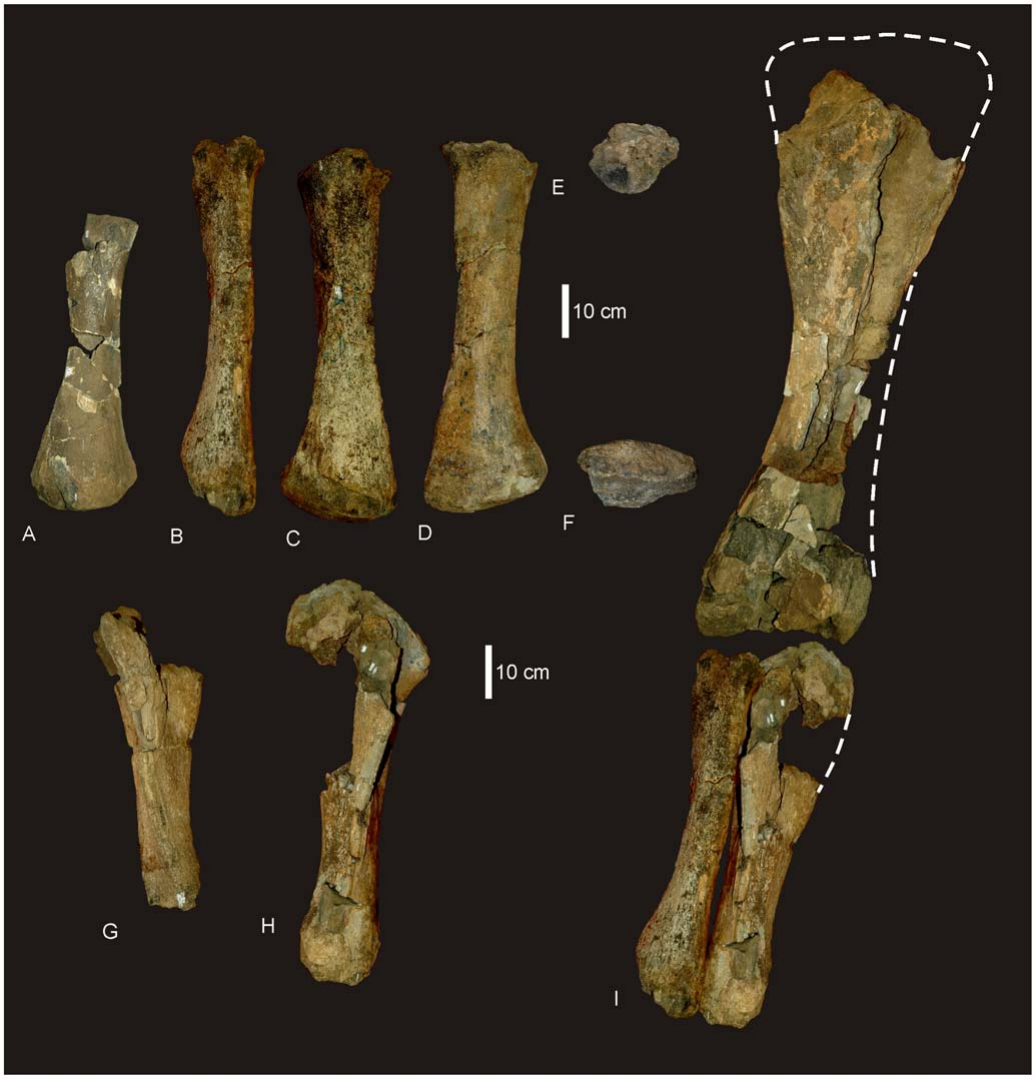
Radii and ulnae of *Wintonotitan wattsi.* Right radius in anterior (A) view. Left radius in medial (B), posterior (C), anterior (D), proximal (E) and distal (F) views. Left ulna in antero-lateral view (G). Right ulna in antero-lateral view (H). Reconstructed left humerus, ulna (reversed H and combined with G) and radius (B) in antero-lateral view. Dashed lines represent suggested missing areas.

The ratio of mid-shaft width to total humerus length is 0.16, which is considered to be a gracile element (<0.20 [15]), however, the proximal breadth to total length ratio equals 0.37, which is considered to represent a stout ulna (>33% [15]). These ratios reflect the narrow and gracile diaphysis of the ulnae, compared to the massively expanded proximal epiphyses, where the lateral and medial processes are extended into narrow crests from the main shaft. The medial process is longer than the other two processes, forming a distinctly tri-radiate cross-sectional shape. Each face is markedly concave with the surface between the medial and lateral processes being deeply excavated, to form the cranial fossa. The surface between medial and caudal processes is similarly excavated, but not to the same degree, whilst the remaining surface between the caudal and lateral process is even shallower. The olecranon process is prominent and extends proximally above the articular surfaces of both the medial and caudal processes. The cranial margin of the medial process is directed medially from the main axis of the shaft. The medial crest is constricted along its length and runs the entire length of the shaft terminating proximal of the distal epiphysis. The caudal process is thick and rounded. Measurements in Table S13.

#### Radii (Figure 17A–F)

A right and left radius are preserved; both are missing the proximal articular ends. The left radius is better preserved than the right, including the distal epiphysis, main shaft and proximal epiphysis. The radius is markedly expanded proximally and distally to a similar degree, with the distal width approximately twice that of the mid-shaft width. The diaphysis is relatively straight only slightly bowed forming a concave posterior face.

The ratio of mid-shaft width to total radius length equals 0.16, which falls only just outside the ratio considered to be gracile (0.15) and below the ratio considered for robust radii (>0.25) [15], therefore the radii are considered here to be gracile, which is reflected in their long and relatively narrow diaphysis. This is in contrast to the markedly expanded epiphyses, similar to the condition in the ulnae.

The distal end is rounded in cranial profile and sub-rectangular in distal cross-section; compressed cranio-caudally, which reflects a more expanded transverse margin; transversely expanded more than the proximal end, which is only expanded medially. The ulnar ligament scar is well developed and extends proximally almost a third of the shaft. A prominent oblique ridge runs from the proximo-caudal face to the medio-distal end. The articular surface of the distal end extends across the entire distal end and up onto the lateral face. A well defined interosseous ridge runs from the medio-caudal margin of the proximal epiphysis to the medial margin of the distal condyle. The proximal end of the radius is slender, possessing a proximal width between 25–26% of the to radius length [15]. Measurements in Table S13.

#### Metacarpus (Figure 18)

A near complete right metacarpus is preserved including metacarpal I–V. Mc I is missing the distal half of the bone, but includes the proximal articular surface and half of the diaphysis. Mc II and IV are missing their proximal articular ends, whilst Mc V is missing the internal portion of the distal articular face. All metacarpals are conspicuously expanded at the proximal and distal ends. Phalangeal facets are absent on all preserved distal ends. Measurements in Table S14.

**Figure 18:**
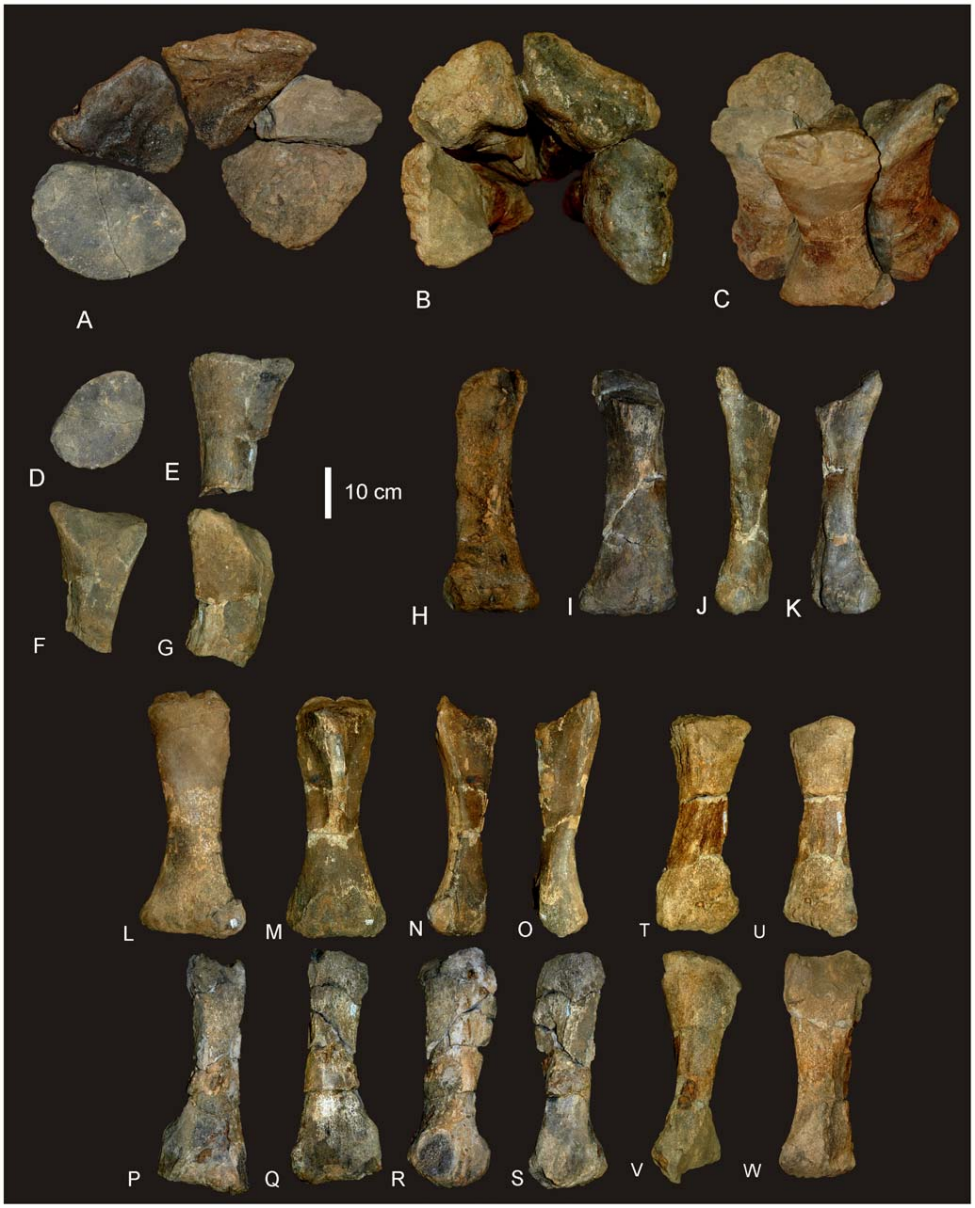
Metacarpals of *Wintonotitan wattsi.* Articulated right metacarpals in proximal (A), distal (B) and antero-external (C) views. Mc I in proximal (D), external (E), internal (F), lateral (G) views. Mc II in external (H), internal (I), lateral (J), medial (K) views. Mc III in external (L), internal (M), lateral (N) and medial (O) views. Mc IV in external (P), internal (Q), lateral (R), medial (S) views. Mc V in external (T), internal (U), lateral (V), medial (W) views.

#### Mc I

Even though Metacarpal I is missing the distal half, it is considered here to be the largest metacarpal in the series, in both length and overall robusticity. The proximal articular surface is massive with rounded, convex, external and posterior faces; concave internal face and a reduced anterior. This characteristic is termed ‘colonnade’ [17] and describes a feature intermediate between ‘D-shaped’ or subrectangular proximal articular faces and reduced semi-circular proximal articular faces. The diaphysis is constricted cranio-caudally and possesses a flat lateral face which articulates with the medial face of Mc II. The lateral margin of the proximal face is concave, and is excavated distally below the cranial and medial articular surfaces.

#### Mc II

Metacarpal 2 is elongate, expanded proximal and distally. The proximal end is quadrangular with the longest side cranio-medially directed. The cranial and caudal surfaces of the shaft are straight and taper to the lateral face, which is rounded. The proximal articular face is not preserved.

The distal end is trapezoidal in shape with rounded margins. The internal face is shorter than the external face, which is missing the cranio-lateral corner. The face is divided into two poorly developed condyles, with a slight middle constriction. The surfaces of the condyles are confluent with each other indicating the absence of any distally articulated phalanges. The external margin of the shaft is shallowly concave and smooth. The internal margin of the shaft possesses a well-developed crest which is broad proximally and extends to the middle of the shaft. On the disto-lateral side of the shaft preserves a distinctive crest which forms what has been interpreted as a flange [17].

#### Mc III

Metacarpal 3 is similar to Mc II, however, differs in the following ways; the proximal cross-section is triangular, not trapezoidal, with the apex of the triangle facing internally, which develops as a posterior crest which extends ventrally past the middle of the shaft. The distal articular surface is similar to Mc II.

#### Mc IV

Metacarpal 4 is missing a significant portion of the proximal articular end, however, the cross-sectional shape points to a very distinctive triangular end which is constricted cranio-caudally so that it fits like a small wedge between Mc III and Mc V. As with Mc III the apex of the triangle faces toward the centre of the metacarpal arcade and produces a distinctive posterior crest which extends ventrally past the middle of the shaft. Anterior to this crest, a low ridge extends ventrally, forming a flat articular surface which contacts the proximal surface of Mc III. Distally, the metacarpal is very robust and cuboid in ventral profile with a distinctive medial condyle which projects into the centre of the metacarpal arcade at the same angle as the posterior crest. The distal articular surface is flat with no indication of a phalangeal articular surface.

#### Mc V

Metacarpal 5 is the shortest metacarpal in the arcade. Proximally, it is the broadest metacarpal except for Mc I, sub-triangular and inflated in proximal profile. The articular surface is preserved and is bulbous and slightly convex in lateral profile. The cranio-medial face is flat to articulate with Mc IV. Distal to this face, a shallow groove locks onto the lateral side of the Mc IV. A cranial crest originates proximal to the middle of the shaft and extends distally to the distal articular surface. A broad crest originates on the internal face of the metacarpal from the proximal end and extends to the middle of the shaft. A third crest originates from the middle of the shaft as a rounded tuberosity and extends distally to the distal articular surface. This third crest is interpreted as the ‘anterior flange’ described in titanosaur metacarpals [17]. The distal articular surface is missing the caudal half; however, the cross-sectional shape is subrectangular with a distinctly flat articular face.

### Pelvic Girdle

#### Ilium (Figure 19A–C)

Two fragments of the left ilium are preserved. These two pieces preserve the preacetabular process, pubic and ischial peduncle, contact zone with the cranial sacral vertebrae and a portion of the acetabulum.

**Figure 19:**
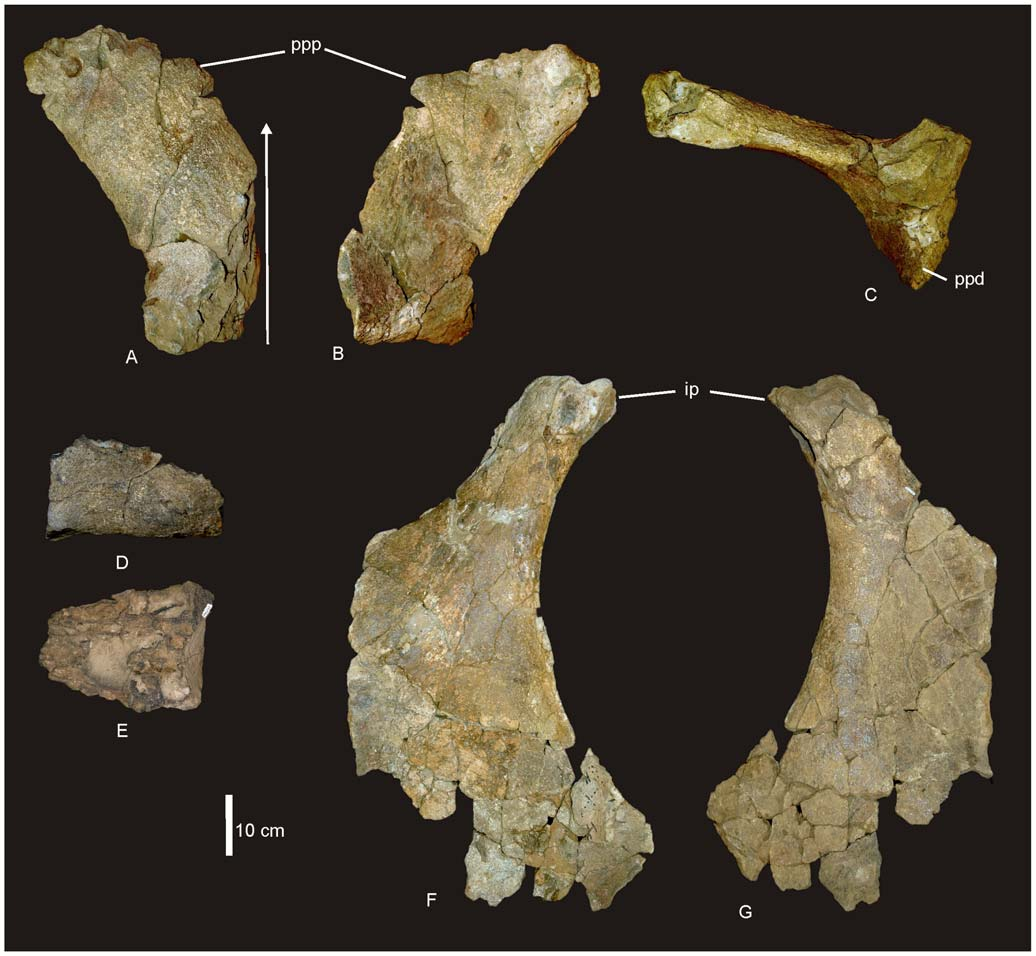
Pelvis of *Wintonotitan wattsi.* Preacetabular lobe of ilium in dorsal (A), ventral (B) and lateral (C) views. Arrow indicates the axis of the sacrum. Partial posterior sacral vertebra in lateral (D) and dorsal (E) views. Left ischium in lateral (F) and medial (G) views. *Abbreviations: ip*, iliac peduncle; *ppd*, pubic peduncle; *ppp*, preacetabular process of ilium.

The preacetabular process is expanded dorso-laterally and is narrow along its length with a rounded tuberosity on the cranial-lateral margin. The highest point on the iliac blade is cranial of the pubic peduncle. The ischial peduncle contributes less to the acetabulum than does the pubic peduncle, which comprises over half of the acetabulum. The pubic peduncle projects perpendicular to the angle of the sacrum. Ischial peduncle low and rounded, medio-laterally elongated. The preacetabular process when resting on the pubic peduncle projects cranio-laterally and flares dorsally. On the medial side of the preserved ilium preserves the contact between the ilium and the sacrum. This contact preserves the distinctive somphospondylus internal bone texture found in the sacral vertebrae, dominated by large camerate vacuities. The preacetabular process also preserves pneumatic chambers within the iliac blade. Measurements in Table S15.

#### Ischium (Figure 19, F & G)

A left ischium is preserved only missing the articular surface of the iliac peduncle and pieces from the distal margin. The ischium is large and expanded ventrally into a broad relatively thin blade. The iliac peduncle is distinct from the neck leading to a large open acetabulum. In cross-section the iliac peduncle is ovoid with a constricted cranial margin, which extends into a sharp crest which borders the acetabular rim and joins the pubic peduncle. The pubic peduncle merges with the pubic articular surface and the distal articular surface into a single straight pubic articulation. In anterior view the pubic articulation is bowed medially and is of a similar thickness along its entire length. Ventral to the pubic articulation the ischial blade is broad and thin, bending laterally toward the distal margin. The distal margin of the ischial blade bends abruptly in a medio-ventral direction. The acetabulum is large and curved cranio-ventrally toward the pubic peduncle. The caudal margin of the ischial blade is long and curved cranially. A distinct triangular process projects medially from the mid-line of the caudal margin of the main ischial blade. The puboischial contact is estimated to be half of the pubis length, where the ischial blade is shorter than the pubic blade. The maximum length of the ischium is 760 mm and its midlength width is 270 mm, providing a ratio of 0.35 for mid-length width versus total length. The length of the pubic articular surface is slightly shorter than the length between the pubic peduncle and caudal margin of the iliac peduncle. Measurements in Table S15.

Theropoda Marsh, 1881
Tetanurae Gauthier, 1986
Allosauroidea Marsh, 1878
*Incertae sedis*

*Australovenator* gen. nov.

urn:lsid:zoobank.org:act:E2C57582-9CDC-4F6A-A3AE-77FA54F37E95

#### Etymology

*Austral, australis – Latin*, meaning southern in reference to the locality being in the Southern Hemisphere, Australia. *Venator – Latin for hunter*. In reference to its carnivorous diet.

#### Type species

*Australovenator wintonensis*

*Australovenator wintonensis* sp. nov.

urn:lsid:zoobank.org:act:37AA3C1B-B498-406B-A51C-C3CF8C88A59F

#### Etymology

From the township of Winton.

#### Holotype

AODF 604: Nine isolated teeth; left dentary; right and left dorsal ribs and rib fragments; right and left gastralial ribs and fragments; partial right ilium; both ulnae; right radius; manus metacarpals, phalanges and unguals; right femur; both tibiae; right fibula; right astragalus; metatarsals, phalanges and unguals (Figure 2D).

#### Type Locality

AODF 85, “Matilda Site”, Elderslie Station, approximately 60 km north-west of Winton, central Queensland, Australia.

#### Horizon & Age

Winton Formation, latest Albian (Cretaceous)

#### Diagnosis

A medium-sized allosauroid, with the following unique association of features. Dentary gracile with sub-parallel margins; rounded dentary symphysis; articular brace (or ‘chin’) absent. 18 tooth loci; alveolus 1 quadrangular; alveoli 2–6 and 12–15 circular; alveoli 7–11 and 16–18 labio-lingually compressed. Interdental plates fused together along the entire length of the dentary. Primary neurovascular foramina row parallel to dorsal margin of dentary, not deflected ventrally. Dorsal ribs with pneumatic cavities. Gastralia unfused with tapered distal ends. Ulna with straight caudal margin, inflated olecranon process; lateral groove along diaphysis (autapomorphic); round and discontinuous lateral tuberosity.

Femur with head directed dorsally in a cranio-medially direction; lesser trochanter developed proximally to the level of the greater trochanter (autapomorphic); distal extensor groove deep and narrow lacking cruciate ridge. Cranio-lateral process of tibia with latero-ventral process; lateral malleolus extends distal of medial malleolus. Proximal articular surface of fibula bevelled cranially (autapomorphic). Astragalus with tall ascending process; medially expanded medial condyle; superior and inferior grooves present on cranial face; cranio-lateral process projects from proximo-cranial margin of lateral condyle. Metatarsals elongate and gracile.

### Description

### Cranial Skeleton

#### Teeth (Figure 20 & 21)

Nine isolated teeth were recovered from the type locality and are all considered to be from the holotype specimen of *Australovenator wintonensis* gen. et sp. nov. All of the teeth have broken away from their bases so that only the tooth crown is preserved. All are recurved and bear fine serrations along mesial and distal carinae. Two teeth have circular to quadrangular shaped bases with two series of serrations; one is a disto-lingual carina and the other is a distal carina. These teeth are considered to be anterior-most dentary or premaxillary teeth. The remaining teeth are all labio-lingually compressed with serrations along the mesial apical edge and along the entire distal carina. On comparison to similar teeth from *Fukuiraptor* and *Neovenator* all of the remaining teeth are considered to be dentary teeth, with rounded mesial and tapered distal margins.

**Figure 20:**
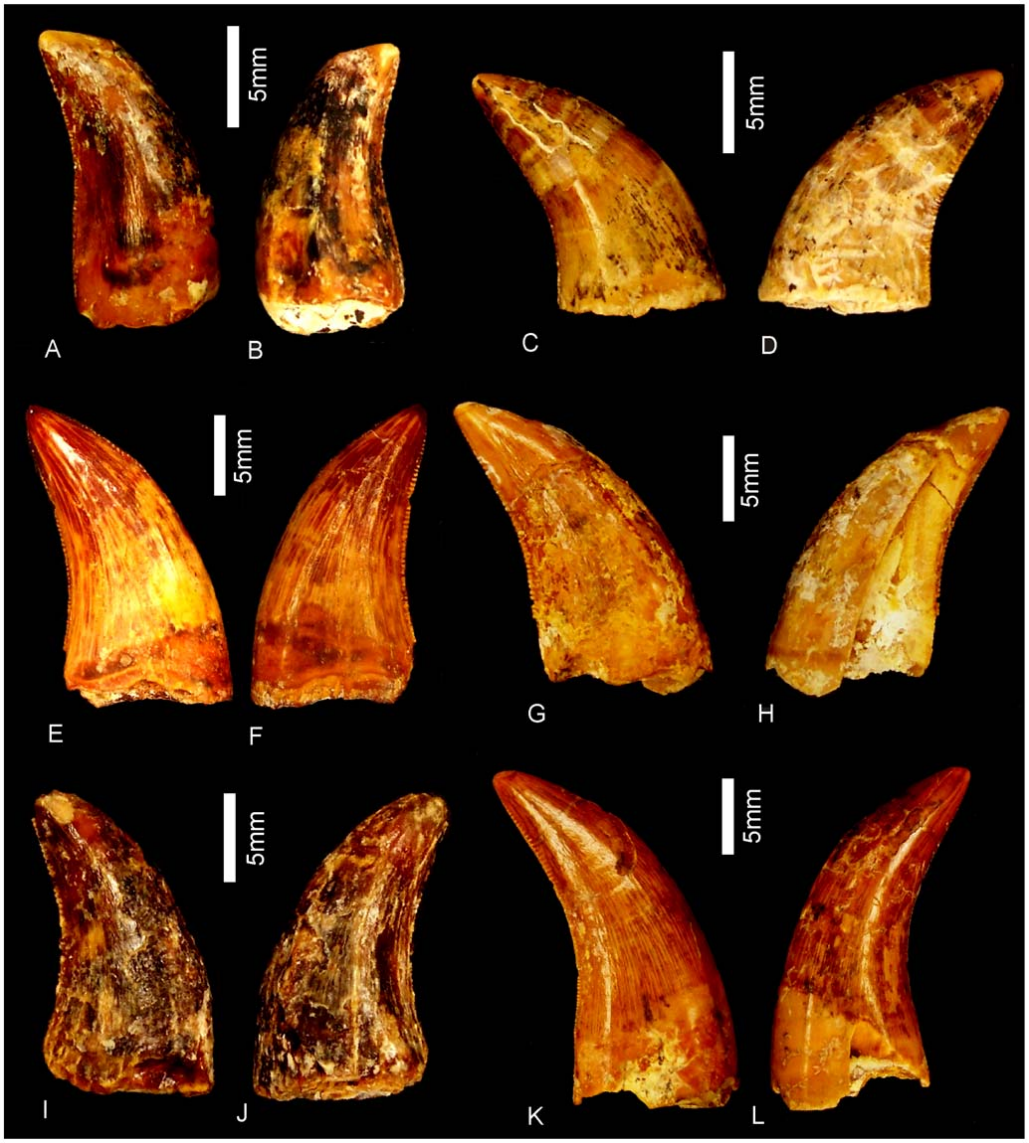
Teeth of *Australovenator wintonensis.* Isolated teeth in labial (A, C, E, F, G, I, J, L) and labial (B, D, F, H, J, K) views. A–B. Anterior dentary tooth or premaxillary tooth. C–L. Dentary teeth.

**Figure 21:**
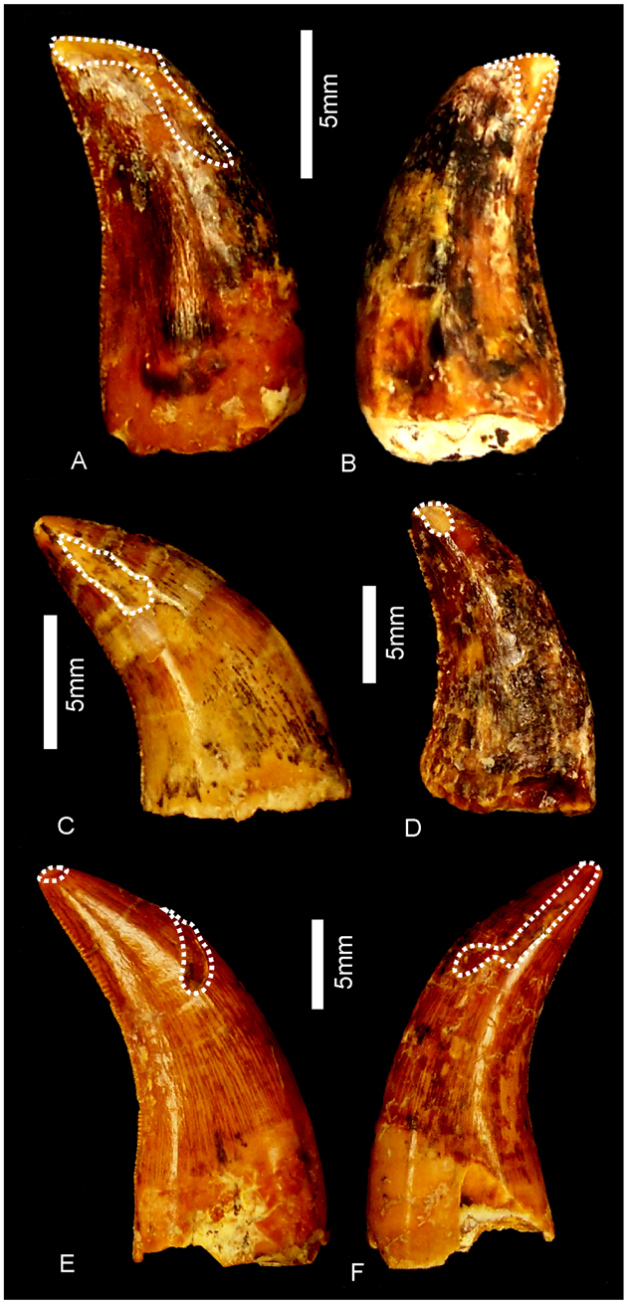
Tooth wear in *Australovenator wintonensis.* Tooth wear facets (dotted line) identified on teeth in labial (A, C, D, F) and lingual (B, E) views.

Tooth measurements of *Fukuiraptor* have been compared to carcharodontosaurids, tyrannosaurids and dromaeosaurids using the FABL (Fore-Aft Crown Base Length) vs BW (Tooth Crown Base Width) measurements as an indicator of labio-lingual compression [19]. It was found that *Fukuiraptor* had more labio-lingually compressed dentition than carcharodontosaurids [19]. Comparing our measurements, we found that the teeth of *Australovenator* were similarly as compressed as *Fukuiraptor*. The teeth do not possess the typical basal crown wrinkles found in carcharodontosaurids, or *Neovenator* [28], [29].

Wear features are noticeable on four of the teeth, including mesial wear across the tip of the tooth crown and mesio-labial wear across the leading edge of the crowns. All preserve weak proximo-distal striae. Measurements in Table S18.

#### Dentary (Figure 22)

A near complete left dentary is known from the holotype (AODF 604). In dorsal view the dentary is slightly curved medially along the posterior half. Eighteen tooth alveoli are present with four alveoli preserving fragmented bases of individual teeth (preserved in alveoli 3, 4, 7 & 11). Each alveolus is a rounded pit bordered lingually by an interdental plate, which is fused on both the anterior and posterior margin to the preceding and succeeding interdental plate, forming an interdental ridge which extends along the entire length of the tooth row. Lingual to this ridge is a shallow dental sulcus.

**Figure 22:**
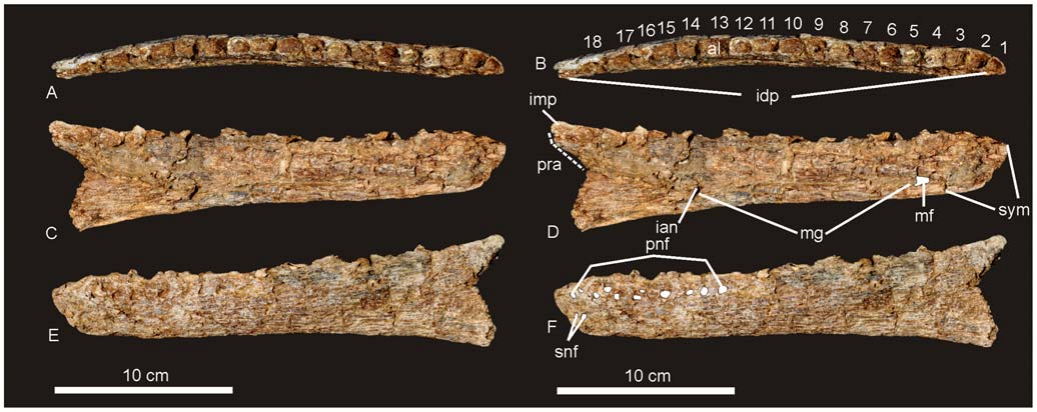
Dentary of *Australovenator wintonensis.* Left dentary in dorsal (A–B), lingual (C–D) and labial (E–F) views. Numbers represent tooth loci (alveoli) position. *Abbreviations: al*, alveolus, *ian*, foramina for branches of the inferior alveolar nerve; *idp*, interdental plates (fused); *imp*, intramandibular process of dentary; *mf*, Meckalian foramen; *mg*, Meckalian groove; *pnf*, primary neurovascular foramina row; *pra*, contact for prearticular; *snf*, secondary neurovascular foramina; *sym*, symphysis.

In dorsal view the first tooth alveolus is small and quadrangular in shape with the antero-labial face longest. Alveoli 2–6 are circular in shape with subequal labio-lingual and antero-posterior diameters. Alveoli 7–11 are labio-lingually compressed, with the long axis oriented antero-posteriorly. Alveoli 12–15 return to a subcircular shape as in alveoli 2–6. Alveoli 16–18 are small, narrowed labio-lingually with the long axis oriented antero-posteriorly.

In lingual view the dorsal and ventral margins of the dentary parallel each other until the position of alveolus 15 where the margins diverge; the ventral margin gently curves ventrally, whilst the dorsal margin remains relatively straight, gently curving dorsally posterior of the last tooth loci. The lingual face of the dentary preserves an anterior dentary symphysis which originates ventrally of the first alveolus; is narrow along its length; follows the anterior margin of the dentary, curving onto the antero-ventral margin and terminating just antero-ventrally of the Meckelian foramen. The Meckelian foramen is exposed and sits immediately anterior of the anterior margin of the Meckelian groove. The Meckelian groove is long, straight, open and dorsal-ventrally narrow, extending posteriorly to a position ventral of tooth loci 14, where it meets a large singular foramen for the inferior alveolar nerve. Posterior of the foramen, the dentary is thin-walled, forming a shallow fossa, which expands posteriorly to the broken posterior margin of the dentary. This expansion is the anterior remnants of the ventral dentary process which connects to the surangular.

The posterior margin of the dentary is made up of a dorsal intramandibular process which extends postero-dorsally. Ventral to this and following the posterior margin of the preserved element is the contact for the prearticular. Ventral to this contact the dentary is broken, missing the posterior process of the surangular contact. In labial view the anterior surface of the dentary is slightly rugose, whilst the posterior margin is relatively smooth. A primary and secondary series of neurovascular foramina are present on the antero-labial face of the dentary. The primary neurovascular foramina are present ventral to the dorsal margin of the dentary, paralleling this margin and not deflected ventrally. Two small secondary neurovascular foramina are located ventral to the anterior-most primary neurovascular foramina. Measurements in Table S17.

### Axial Skeleton

#### Dorsal Ribs (Figure 23A–F)

Several dorsal ribs are preserved including the first right dorsal rib, second or third right dorsal rib and seventh or eighth left dorsal rib. Several isolated rib shaft pieces are also known. All rib elements are missing the distal ends. Comparisons were made to rib sequences in *Allosaurus* [20], *Sinraptor* [21] and *Acrocanthosaurus* [22] to determine the position of the ribs preserving proximal ends. Measurements in Table S18.

**Figure 23:**
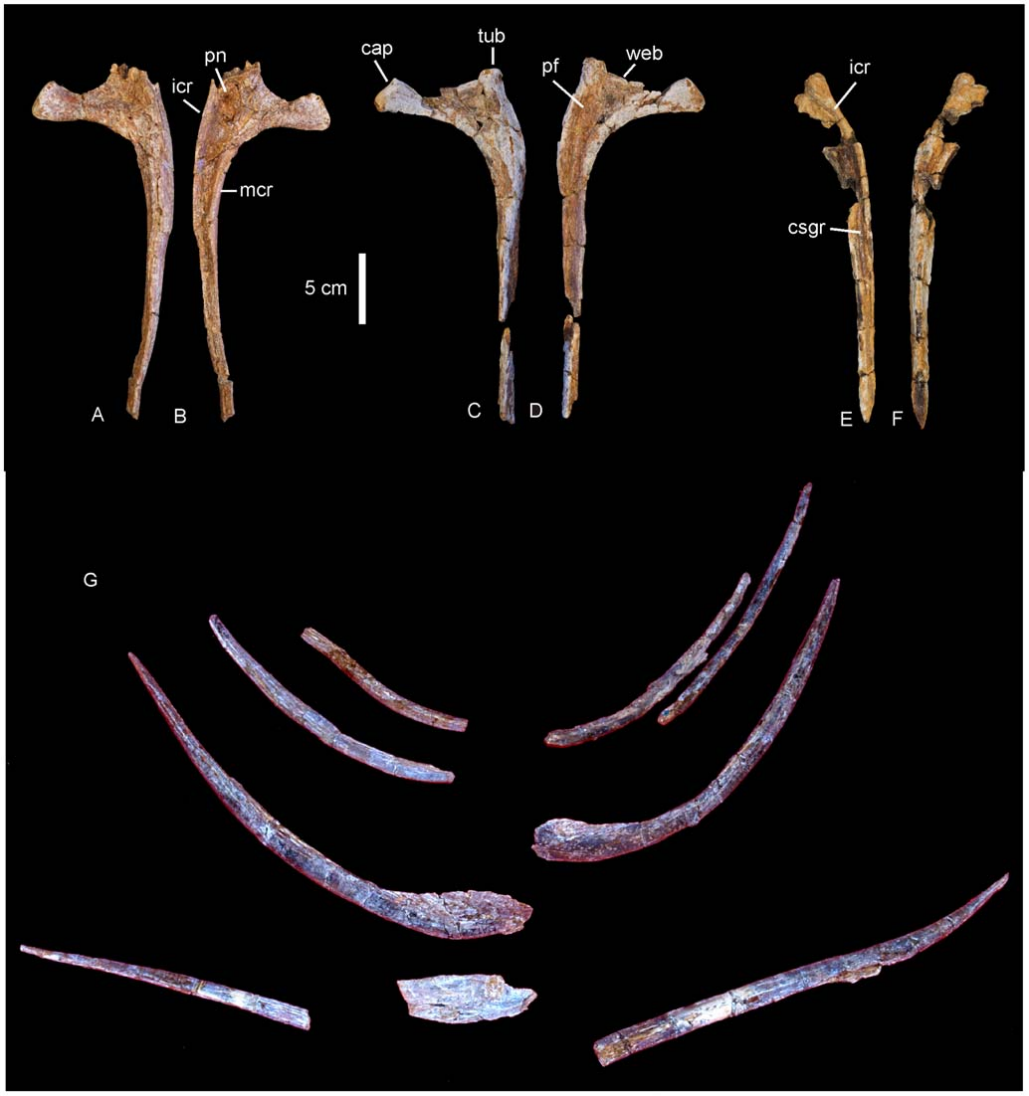
Dorsal ribs of *Australovenator wintonensis.* 1^st^ right dorsal rib in posterior (A) and anterior (B) views. 2^nd^ or 3^rd^ right dorsal rib in posterior (C) and anterior (D) views. 7^th^ or 8^th^ left dorsal rib in anterior (E) and posterior (F) views. Gastralia of *Australovenator wintonensis.* Gastralia arranged in right and left position in dorsal view (G). *Abbreviations: cap*, capitulum; *csgr*, costal groove; *icr*, intercostal ridge; *mcr*, medial costal ridge; *pn*, pneumatic pore and cavity; *tub*, tuberculum; *web*, capitulo-tubercular web.

#### First right dorsal rib

Gracile dorsal rib expanded proximally into a broad capitulo-tubercular web. Proximal-most tubercular margin extends above the proximal-most margin of the capitulum. Tuberculum projects dorsally, broadens medio-laterally and is narrow cranio-caudally. Anterior face of tuberculum developed into a shallow fossa, which connects distally to the costal groove. Fossa bordered by a cranio-caudally expanded intercostal ridge. Proximal articular end of the tuberculum is missing. Capitulum projects medially, almost perpendicular to the dorso-ventral axis of the tuberculum. Capitulum broadest dorso-ventrally and cranio-caudally narrow. Proximal articular end of capitulum flattened cranially and convex caudally with rounded articular facet. Capitulo-tubercular web thin with a concave proximal margin in anterior view. Neck of capitulum narrower than proximal-most end. Costal groove extends from the tubercular fossa distally along the entire length of the preserved rib shaft. Groove narrows along its length. Costal groove bordered laterally by a distally tapering intercostal ridge and medially by a similar proximally thick medial ridge. Both these ridges coalesce distally, only divided by a narrow costal groove. In posterior view there is a distinct costal groove which originates ventro-laterally below the caudal face of the capitulo-tubercular web and runs distally along the postero-medial edge of the rib shaft. It terminates proximal of the preserved distal end. Rib shaft ‘v’ shaped in proximal cross-sectional profile. Pneumatic pore absent. In lateral view the rib shaft bows caudally.

#### Second or third right dorsal rib

Similar in morphology to first right dorsal rib with the following differences; in cranial view, tuberculum smaller than capitulum where the proximal-most margin of the tuberculum is lower than the proximal-most margin of the capitulum; tuberculum a small rounded knob with the long axis projecting antero-laterally; capitulo-tubercular web broader with a shallow concave proximal margin; fossa on cranial face of tuberculum shallower; proximal section of rib shaft broader cranio-caudally.

#### Seventh or eighth left dorsal rib

Similar in morphology to the right second or third dorsal rib with the following differences; intercostal ridge extends proximally to connect to the tuberculum; costal groove deeper.

#### Gastralia (Figure 23G)

Nine elements representing gastralial ribs are preserved including two complete gastralia with proximal articulations, three near complete gastralia without proximal ends, one isolated proximal end and three shaft fragments. The longest gastralia are bowed posteriorly throughout the midline of the rib shaft, and then curve anteriorly to an equal degree, forming a shallow sigmoidal shape in dorsal view. Eight specimens are medial gastralia and a single shaft, which is tapered at both ends, is considered to be a lateral gastralium. Three medial gastralia are considered to be anterior gastralia. These specimens include two shafts and an isolated proximal articular expanded surface. The two shafts are thick and rounded in cross-section being shallowly sigmoidal in dorsal view. The distal ends taper to a fine point. A long cranio-lateral groove runs nearly the entire length of the shaft.

When viewed dorsally, the proximal ends of the gastralia are expanded cranio-caudally and dorso-ventrally compressed into a thin spatulate shape. The medio-dorsal facet is shallow and ovoid, bordered by a fine ridge running diagonally over the proximal expansion. The ventral face of the proximal end bears fine rugosities.

#### Ilium (Figure 24)

A portion of a right ilium is preserved with broken medial and ventral surfaces exposing the pneumatic internal structure of the ilium. The preacetabular expansion is broken and has collapsed ventrally. The pubic peduncle is missing from the cranial margin of the acetabulum. The ischial peduncle is partially preserved; however, it has been distorted dorsally above the line of the acetabular fossa. The acetabulum is narrow cranially and expanded caudally with a concave dorsal surface. The postacetabular process and iliac blade are missing. Measurements in Table S19.

**Figure 24:**
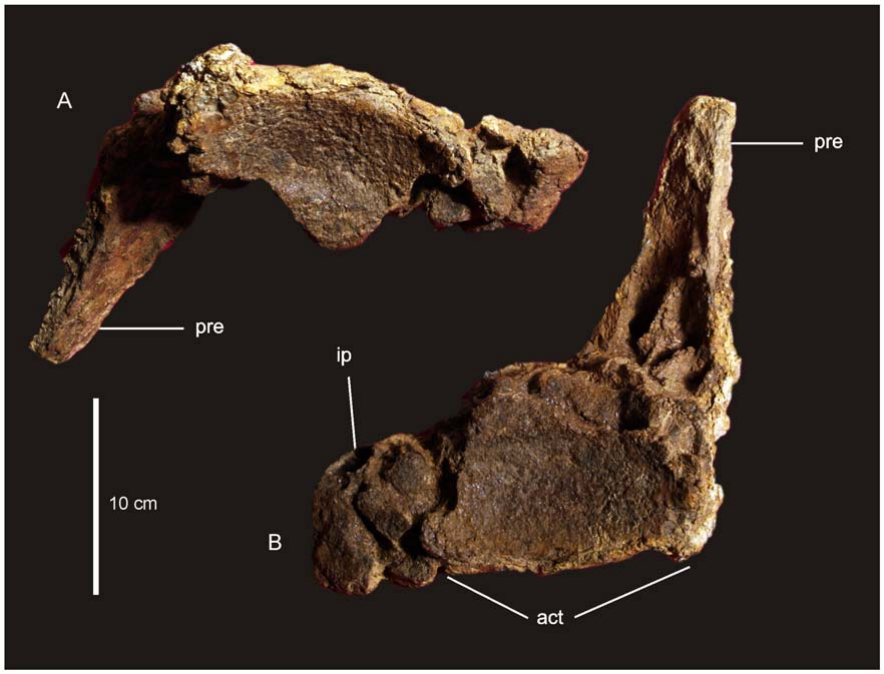
Ilium of *Australovenator wintonensis.* Partial right ilium in lateral (A) and ventral (B) views. *Abbreviations: act*, acetabulum; *ip*, ischial peduncle; *pre*, preacetabular process.

### Appendicular Skeleton

#### Ulnae (Figure 25)

Both ulnae are known from the holotype (AODF 604). Proximally the ulnae are expanded with well-developed and rounded olecranon processes. The proximal humeral articular surface is divided into two distinct fossae separated by a median ridge. The ulnar shaft is slightly bowed caudally and arched proximally. The ulnar shaft diameter is similar to that of the radius. The distal condyle is sub-triangular in distal profile with distinct dorso-medial and medial processes. The distal radial facet is transversely expanded and concave. The proximal and distal ends are both expanded relative to the mid-shaft width. In lateral view the caudal surface of the proximal end is straight, whilst the cranial surface is distinctly curved proximally toward the coronoid process. The olecranon process is well developed and rounded extending proximal of the humeral articular surface. In lateral aspect the olecranon is flat proximally with rounded cranial and caudal corners. In proximal view the olecranon process is moderately inflated medio-laterally and cranio-caudally. The medial surface is relatively flat as a continuation of the proximal face of the shaft. The long axis of the olecranon process is oriented cranio-caudally, along the same axis developed by the cranial expansion of the coronoid process. The olecranon process extends as a rounded buttress on the caudal surface of the shaft.

**Figure 25:**
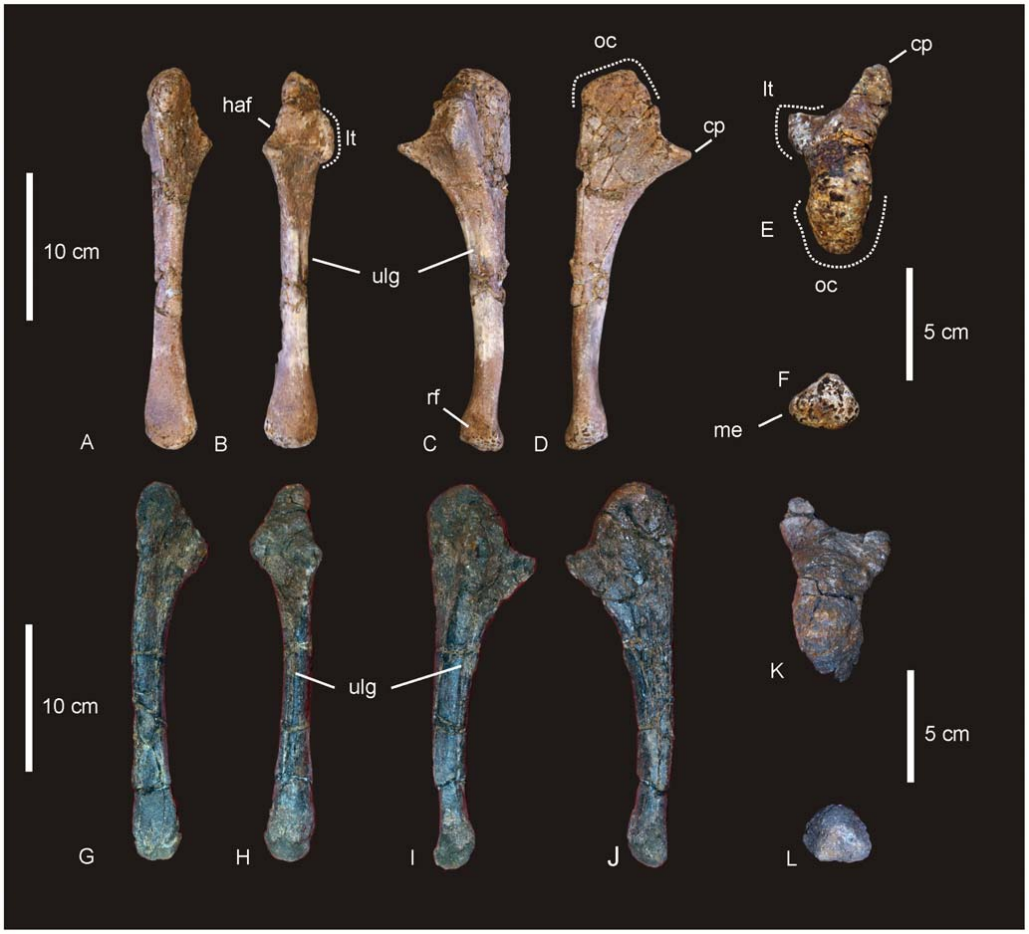
Ulnae of *Australovenator wintonensis.* Left and right ulnae in posterior (A, G), anterior (B, H), lateral (C, D), medial (D, J), proximal (E, K) and distal (F, L) views. *Abbreviations: cp*, coronoid process; *haf*, humeral articular facet; *lt*, lateral tuberosity; *me*, medial expansion of distal end; *oc*, olecranon process; *ulg*, lateral groove; *rf*, radial facet.

The lateral (radial) tuberosity is distinct and developed cranio-laterally into a stout rounded process; its proximo-cranial surface merges with the articular surface. The distal margin terminates proximal of the main shaft. Caudal to the lateral tuberosity the shaft is rounded. The coronoid process projects cranially as a triangular wedge. In proximal view, the coronoid process is ovoid with the articular surface extending to its cranial tip. The coronoid process is extended and rounded medially. A shallow groove on the cranio-lateral face of the shaft runs from the proximal 1/3 of the bone to the mid-shaft where it develops into a low ridge, which extends to a ridge proximal to the radial facet.

The distal extremity is medio-laterally expanded relative to the mid-shaft diameter and possessing a medial and cranial expansion of the epiphysis. In distal view the articular surface is subtriangular with an inflated and rounded caudal margin which forms the longest side. The cranio-lateral face is straight projecting cranially to a rounded apex. The cranio-medial face is the shortest side and is slightly concave extending toward the cranial apex.

In lateral and medial views the distal epiphysis is convex caudally producing a distinct condyle. The cranio-lateral surface of the distal epiphysis is a shallow concave facet extending proximally to a low ridge that encloses the distal radial articular facet. Measurements in Table S20.

#### Right Radius (Figure 26)

A single radius is present in the holotype (AODF 604). The bone is elongate with expanded proximal and distal articular ends; distal end expanded more than proximal end; proximal end flat and distal end rounded. The diaphysis is straight and sub-circular in cross-section. The caudal face of the shaft flattens toward the distal end. The proximal end is cranio-caudally expanded and ovoid in proximal view. The caudal margin is rounded and broader than the cranial margin. The distal end is expanded medio-laterally with two distinct condyles. The larger lateral condyle is sub-circular and is medially attached to a smaller medial condyle. The medial condyle is very small and caudally projected. The cranial margin of lateral condyle is developed into a small point which is the distal termination of a low ridge from the cranial face of the shaft. In cranial aspect the distal expansion borders the distal radial-ulnar facet, which is flat and bounded within a low ridge running toward the medial condyle and second ridge running to the margin of the lateral condyle. Measurements in Table S20.

**Figure 26:**
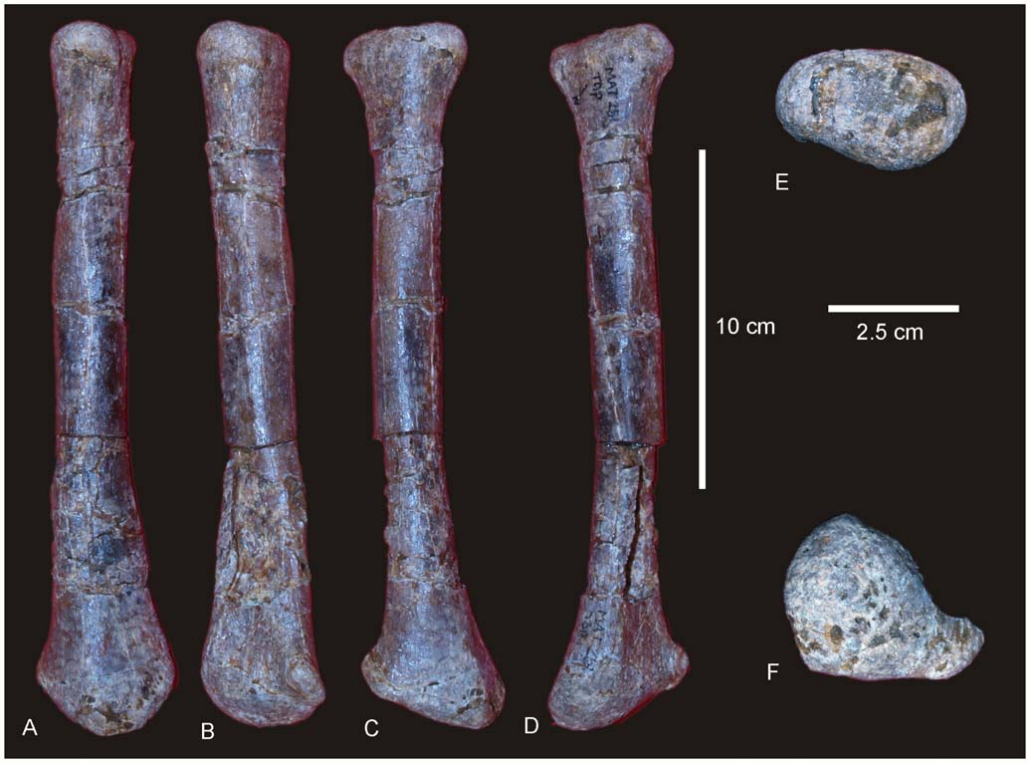
Radius of *Australovenator wintonensis.* Right radius in anterior (A), posterior (B), lateral (C), medial (D), proximal (E) and distal (F) views.

#### Manus

Elements from three digits are known from the manus of the holotype (AODF 604), each with a recurved and pointed terminal ungual. Measurements in Table S20.

#### Left Mc I (Figure 27A–F)

Metacarpal I is the shortest of the preserved metacarpals. It is badly preserved, however, it possesses the distal articular condyles and the proximal articular surface. Mc I is a robust element with a straight lateral margin and concave medial margin. The proximal end is produced medially into a medial condyle, which is rounded and flat proximally. The proximal articular face is concave in dorsal aspect. The distal end is divided into two distinct condyles with the medial condyle largest and offset from the main axis of the metacarpal by 45°. The entire condyle is bevelled medially with a ventrally expanded process. The lateral condyle is oriented along the long axis of the metacarpal. The lateral condyle is smaller than the medial condyle and not as expanded ventrally. A deep internal groove divides the two distal condyles. The proximo-lateral margin is badly preserved and is missing its dorsal face. The bone shape suggests that the dorsal margin was expanded into a proximo-dorsal crest which sat below the proximo-medial crest of Mc II.

**Figure 27:**
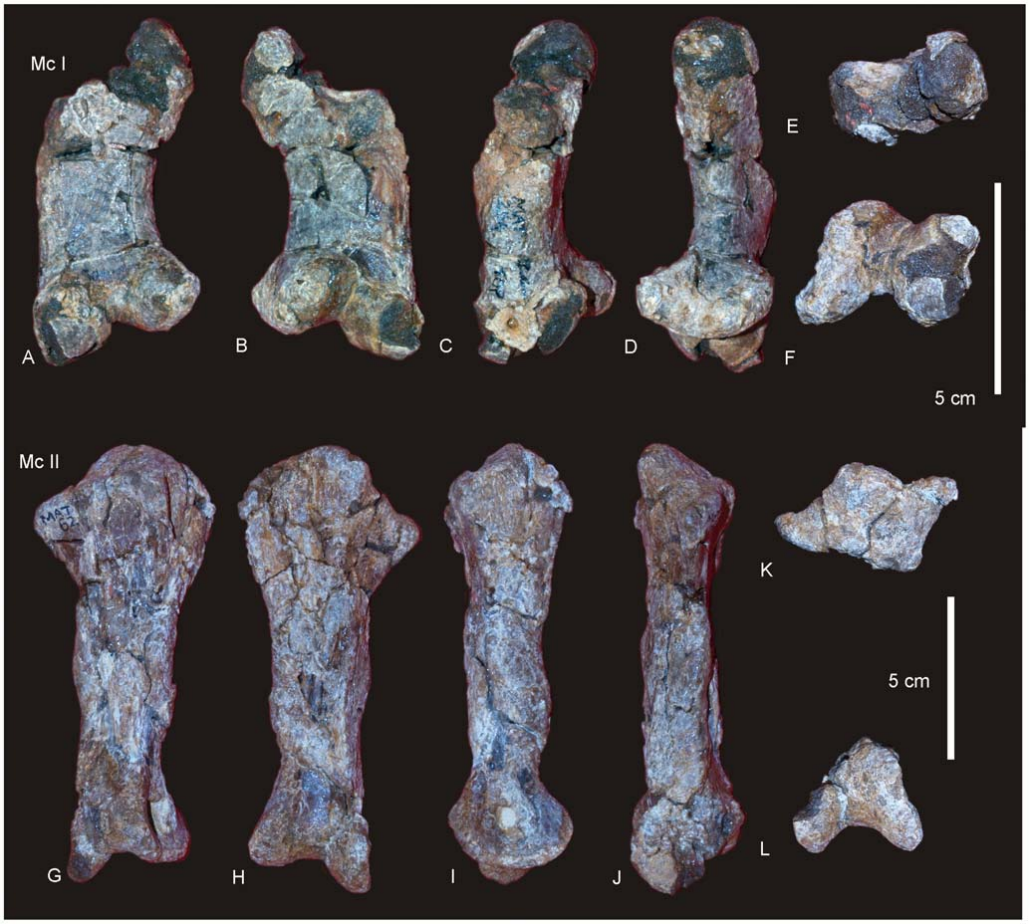
Metacarpals of *Australovenator wintonensis.* Mc I in ventral (A), dorsal (B), lateral (C), medial (D), proximal (E) and distal (F) views. Mc II in dorsal (G), ventral (H), lateral (I) medial (J), proximal (K), distal (L).

#### Left Mc II (Figure 27G–L)

Metacarpal II is approximately twice the length of Mc-I. The metacarpal shaft is straight and quadrangular in mid-shaft cross-section. The proximal end is transversely expanded, producing a medial crest which sits above Mc I. The proximal end is divided into four processes, a medial, dorsal, lateral and ventral process. The medial process is longest and the most developed of all four, extending medially and curving ventrally at its medial margin, forming a concave ventral face of the medial process. The dorsal face of the medial process is shallowly concave. The dorsal process is low and rounded, bordered by the dorso-medial and dorso-lateral faces of the proximal end. In dorsal view the dorsal process extends distally as a low process and is bowed laterally. The lateral process is less developed than the medial process and is narrow dorso-ventrally. It is bordered by the dorso-lateral and ventro-lateral faces. Both of these faces are concave in proximal view. Both faces form shallow fossae along the dorsal and ventral surfaces of the metacarpal shaft. The ventral process is directly opposite the dorsal process, and like the dorsal process it is low and rounded, bordered by the ventro-lateral and ventro-medial faces. The ventro-medial face possesses a shallow fossa which received the dorsal crest from Mc I. The distal end of the metacarpal is slightly expanded and divided into two distinct condyles. The medial condyle is missing the dorsal margin; however, it would be larger than the lateral condyle. The internal groove is deep and broad. A deep ligament insertion pit is present on the lateral margin of the lateral condyle.

#### Manus Phalanges and Unguals (Figures 28 & 29)

All of the preserved digital phalanges are long and narrow bones, expanded at both the proximal and distal ends with articular proximal facets and rounded distal condyles. The holotype (AODF 604) preserves two largest phalanges corresponding to Mc I-1 of the right and left manus. A third phalange is approximately 2/3 the size of Mc I-1 and corresponds to Mc II-2. A fourth smaller phalange is 1/3 the size of Mc I-1 and corresponds to Mc III-3. This phalange articulates with the smallest preserved ungual, Mc III-4.

**Figure 28:**
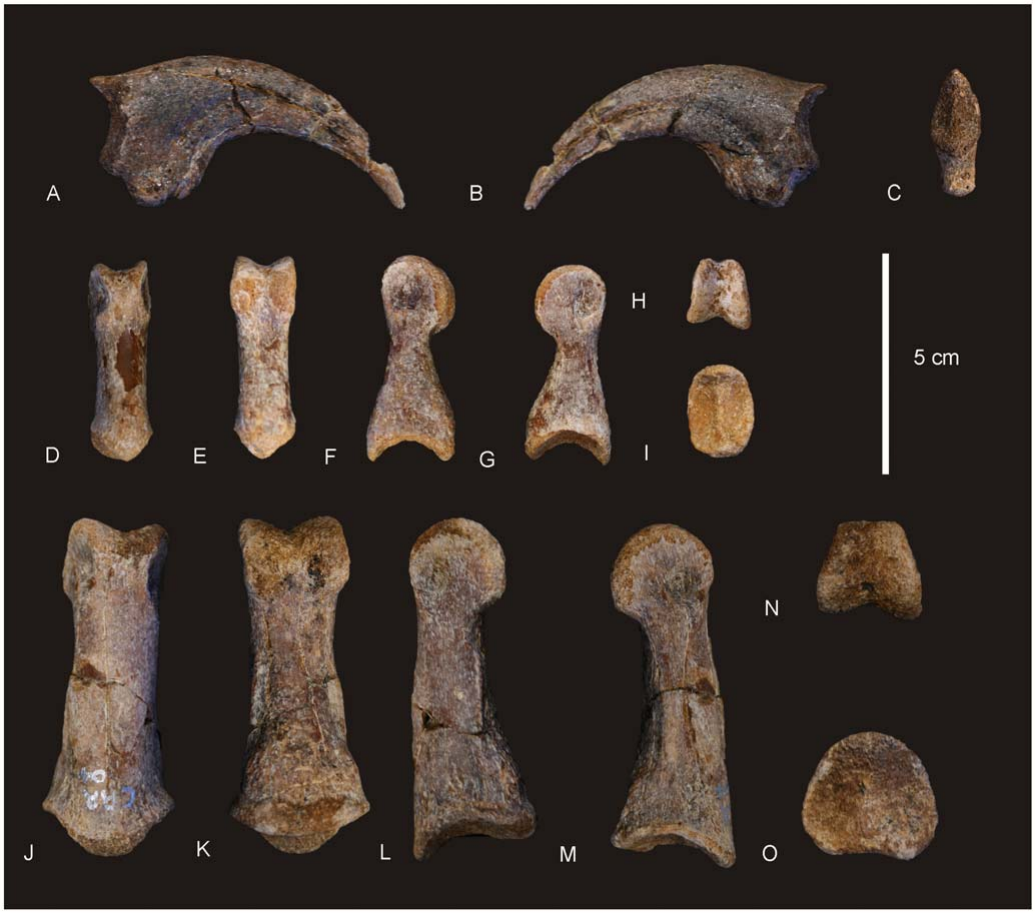
Manus of *Australovenator wintonensis.* Mc III.4 in lateral (A), media (B) and proximal (C) views. Mc III.3 in dorsal (D), ventral (E), lateral (F), medial (G), distal (H), proximal (I). Mc II.2 in dorsal (J), ventral (K), lateral (L), medial (M), distal (N), proximal (O).

**Figure 29:**
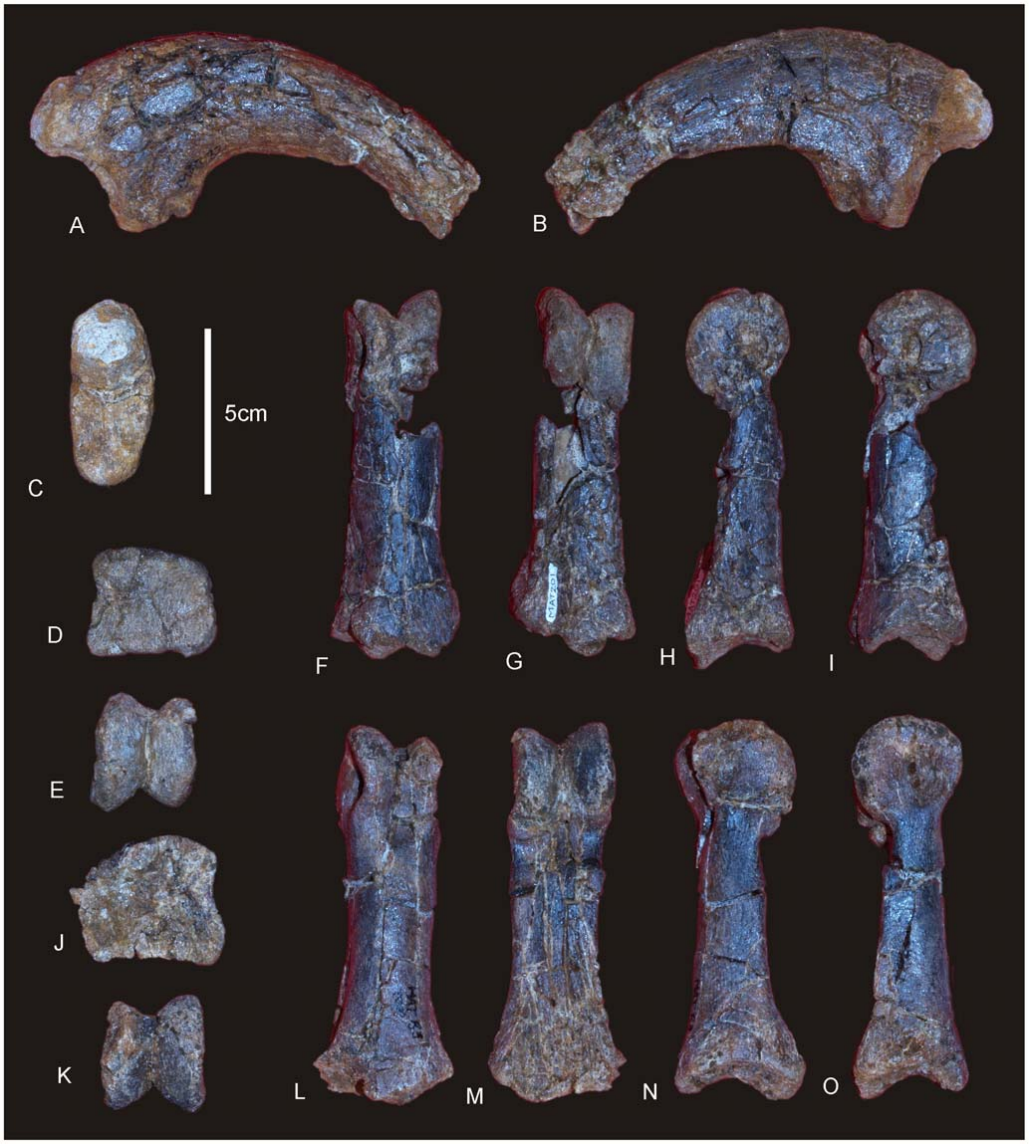
Manus of *Australovenator wintonensis.* Mc I.2 in lateral (A), medial (B) and proximal (C) views. Left Mc I.1 in proximal (D), distal (E), dorsal (F), ventral (G), lateral (H) and medial (I) views. Right Mc I.1 in proximal (J), distal (K), dorsal (L), ventral (M), lateral (N) and medial (O) views.

#### Unguals (Figures 28 & 29)

Three unguals are preserved. Two are right unguals and a third is too badly preserved to determine its placement, other than that is most-likely the tip off of Mc II-3.

Mc I-2 is a very large ungual, laterally compressed and recurved ventrally from a tall and broad articular facet to a tapered sharp point. The proximal articular facet is deeply concave and divided into two by a median ridge. The articular surface for the medial condyle has a greater dorso-ventral expression than the lateral condyle. The medial face of the ungual is relatively flat in comparison to the lateral face which is rounded and possesses a deeper vascular groove. A sharp crest extends from the medial base of the flexor tuberosity and follows the concave ventral margin to the distal tip. Flexor tubercles are well developed and prominent. Lateral and medial vascular grooves are similarly well developed.

The smallest preserved manus ungual is laterally compressed, recurved and tapers to a sharp fine point. Overall it has a similar shape to Mc I-2 being approximately half of its length.

#### Femur (Figure 30)

The right femur of the holotype (AODF 604) is complete. In lateral view the femur is straight from the proximal end to the distal margin of the fourth trochanter; the remaining shaft then bows cranially. The shaft is sub-circular in mid-shaft cross-section with a flattened caudal face. In anterior view the shaft is straight and the femoral head projects dorso-medially from the main shaft. In proximal view the femoral head is oriented cranio-medially. The caudal flange of the caput borders a deep groove. The groove runs horizontally in a proximo-medial to disto-lateral direction onto the caudal face of the proximal articular end. The caput is rounded, and in medial view is circular. The distal margin of the caput overhangs a ventral groove that extends from the caudal flange around the base of the femoral head onto the cranial face of the proximal end. The accompanying groove is deep and undercuts the rounded caput. The femoral head is subequal along most of its length; being broader medially via an inflated caput. The lateral face of the greater trochanter is cranio-caudally as wide as the lateral face of the lesser trochanter. The lesser trochanter is a flat sub-rectangular ‘tongue-like’ flange oriented cranio-caudally from the lateral margin of the proximo-cranial face of the shaft. The lesser trochanter is cranio-caudally longer than medio-laterally wide, extending proximally to just below the distal extremity of the proximal-most lateral margin. This is achieved by the development of a proximal tuberosity which extends above the lesser trochanter. The cranial accessory trochanter is a simple bulbous ridge that extends in a proximo-distal direction along the cranio-distal margin of the lesser trochanter. It is greatly reduced and not developed into a fully triangular process. The trochanteric shelf is a broad, rounded protuberance which extends distally as a low ridge, merging into the shaft below the level of the fourth trochanter. There is a deep sulcus between the trochanteric shelf and the fourth trochanter. The fourth trochanter originates from the caudal surface of the shaft as an ovoid projection. The lateral face of the trochanter is heavily rugose. Medially, the fourth trochanter is divided by a median groove running proximo-distally.

**Figure 30:**
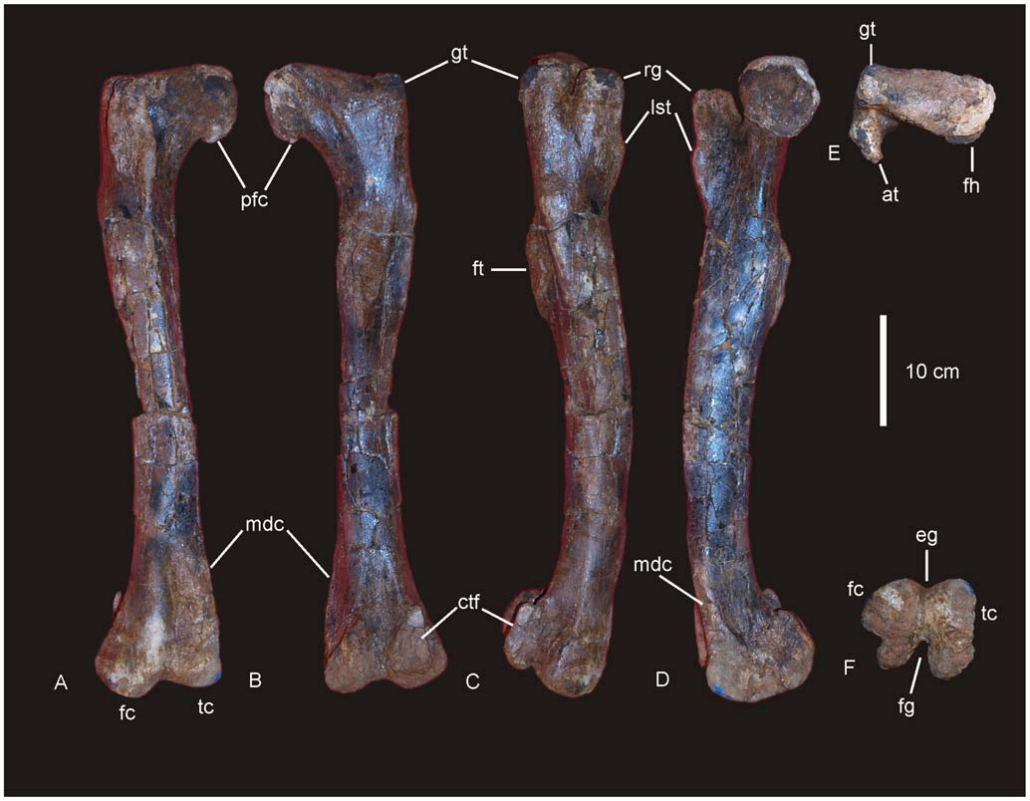
Femur of *Australovenator wintonensis.* Right femur in anterior (A), posterior (B), lateral (C), medial (D), proximal (E) and distal (F) views. *Abbreviations: at*, accessory trochanter; *ctf*, crista tibiofibularis; *eg*, extensor groove*; fc*, fibular condyle, *fg*, flexor groove; *fh*, femoral head (caput); *ft*, forth trochanter; *gt*, greater trochanter; *lst*; lessor trochanter; *mdc*, medial-distal (epicondyle) crest; *pfc*, posterior flange of caput; *rg*, ridge superior to lesser trochanter; *tc*, tibial condyle.

The distal end of the femoral shaft is expanded transversely and cranio-caudally rounded. The medial distal crest (epicondyle) is distinct and broad, running from the cranial face of the shaft across the medial edge to the medial (tibial) condyle. The extensor groove is deep and narrow. The ridge for the cruciate ligaments is absent. The distal lateral condyle is bulbous. Measurements in Table S21.

#### Tibiae (Figure 31)

Both tibiae are known from AODF 604. The tibia is long and gracile, bowed laterally and slightly caudally. The proximal end is expanded cranio-caudally whilst the distal end is expanded medio-laterally. The shaft is flat along its cranial face and rounded on its caudal, medial and lateral faces so that the mid-shaft cross-section is sub-circular with a flattened cranial side. A fibular groove runs from the distal end of the fibular flange to the distal expansion where it meets the astragalar facet. The cross-section of the shaft is increasingly narrowed in the cranio-caudal plane as it extends distally to the distal expansion for the astragalus and calcaneum. The cnemial crest is proximal-most, angled dorso-laterally, with an accessory postero-ventral ridge running diagonally toward the centre of the cnemial crest and bordering the proximal opening of the *incisura tibialis*. The lateral condyle is large and circular in anterior view, bordered to its caudal margin by the proximal articular surface which is indented and grooved. Cranial to the lateral condyle is a small process, the cranio-lateral process, which is directed cranio-distally, over hanging the cranio-lateral face of the shaft and bordering the medial side of the *incisura tibialis*. Directly distal to the cranio-lateral process is a flat section of the shaft which extends into a crest which meets with the fibular flange. The fibular flange projects laterally to the same level as the lateral condyle and is distally extended into a long crest which follows the curvature of the shaft. Caudal of the fibular groove a distinct ridge buttresses the position of the fibula along its length. Medial to the lateral condyle is the medial condyle which is not as rounded or as large as the lateral condyle and merges with the main proximal articular surface. The medial condyle is rounded in posterior view with an accessory process which projects laterally. This process borders the medio-lateral ‘notch’ which divides the medial condyle from the lateral condyle. This notch in *Australovenator wintonensis* is more of a slight indentation, rather than a distinct notch as in *Neovenator salerii*. The caudal face of the proximal end is blade-like with a flat face.

**Figure 31:**
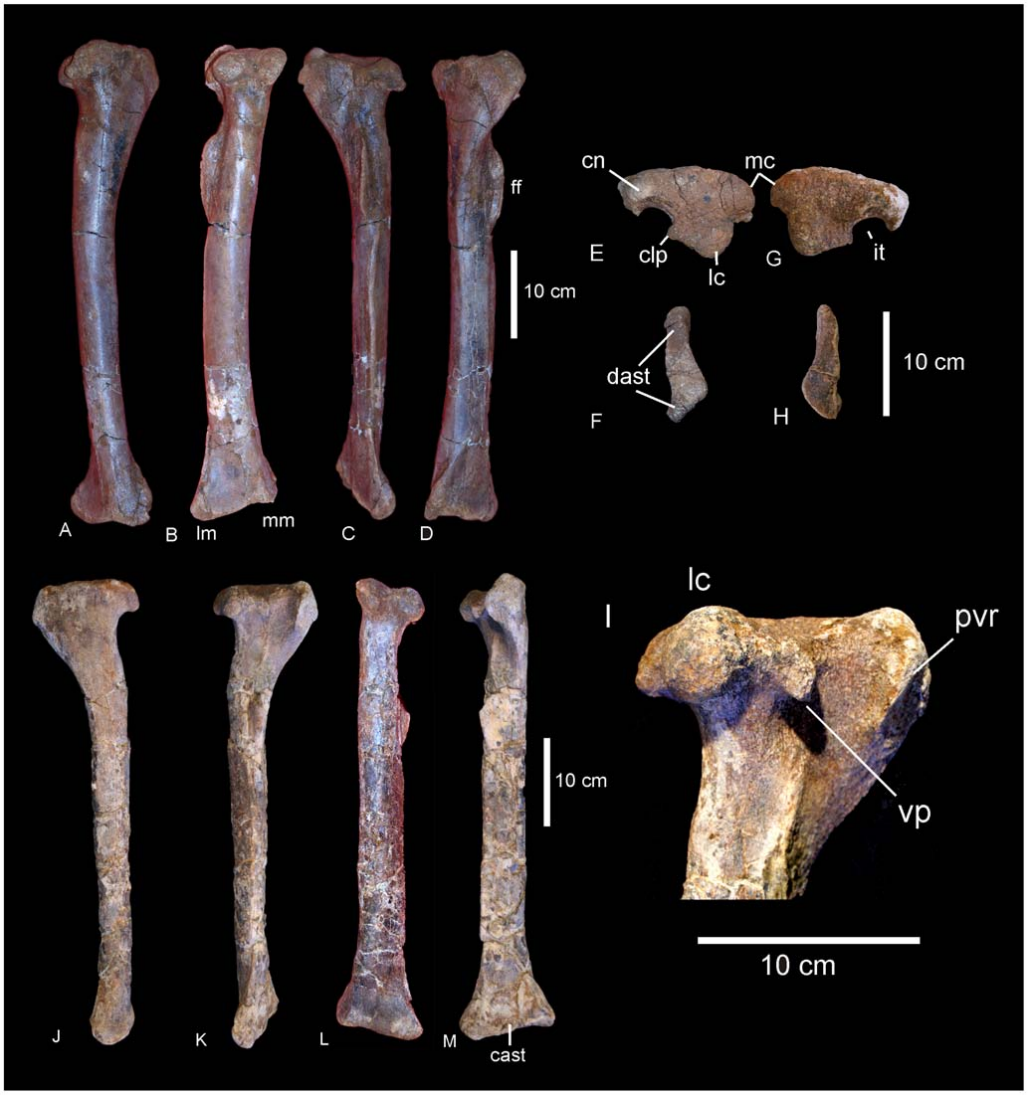
Tibiae of *Australovenator wintonensis.* Left tibia in antero-medial (A), postero-lateral (B), lateral (C), postero-medial (D), proximal (E) and distal (F) views. Right tibia in proximal (G), distal (H), medial (J), medial (K), posterior (L), anterior (M) views and close-up of proximal end in postero-lateral view (I). *Abbreviations: cn*, cnemial crest; *clp*, cranio-lateral process; *cast*, cranial astragalar facet; *dast*, astragalar facet (distal); *ff*, fibular flange; *it*, incisura tibialis; *lc*, lateral condyle; *lm*, lateral malleolus; *mc*, medial condyle; *mm*, medial malleolus; *pvr*, postero-ventral ridge; *vp*, ventral process of lateral condyle.

The distal end is expanded medio-laterally and possesses a distinct astragalar facet. The astragalar facet is tall and triangular with a rounded proximal margin. The lateral malleolus extends distally of the fibular facet and is present on the distal-most expansion. The lateral malleolus is compressed cranio-caudally to a greater degree than the medial malleolus. The medial malleolus is expanded caudally and rounded medially, with a proximal extension that develops into a small thin crest extending diagonally onto the cranial face of the shaft. This crest borders the disto-medial margin of the facet for the ascending process of the astragalus. A sharp straight crest extends from the disto-lateral side of the fibular facet, running distally along the lateral flank of the tibia toward the lateral malleolus, fading out just proximal of it. Measurements in Table S22.

#### Fibula (Figure 32)

The right fibula is complete and well preserved. In proximal view the proximal end is expanded cranio-caudally into a broad triangular shape which is rounded cranio-laterally and pointed medio-caudally. The cranial margin is rounded and thick, tapering posteriorly to a thin compressed caudal process. The articular surface is flat and extends across the entire length of the proximal end. The proximal articular surface is bevelled slightly cranially so that the caudal margin of the proximal articular surface lies above the cranial margin. Distal to the proximo-cranial margin a triangular process projects cranially, expanding the proximal end and developing a distinct concave profile of the proximo-cranial margin of the shaft. The shaft is long and straight with a slight lateral bow. The shaft tapers distally then expands to an inflated distal articular end. The lateral face of the proximal expansion is rounded the medial face concave, forming a proximo-distally elongate fossa, which is ovoid in medial view. Distal to this is a groove which extends to the distal end of the shaft. As the groove reaches the distal expansion it flattens into an articular facet which inserts into the fibular facet of the tibia and astragalus. The groove is bordered cranially and caudally by thick ridges. The cranial ridge continues distally onto the distal expansion. The distal expansion is kinked and expanded laterally so that the distal articular face is sub-triangular in shape. A rounded tuberosity, between the proximal expansion and the middle of the shaft, is expanded cranio-laterally, forming the attachment point for the *interosseum tibiofibulare*. Measurements in Table S23.

**Figure 32:**
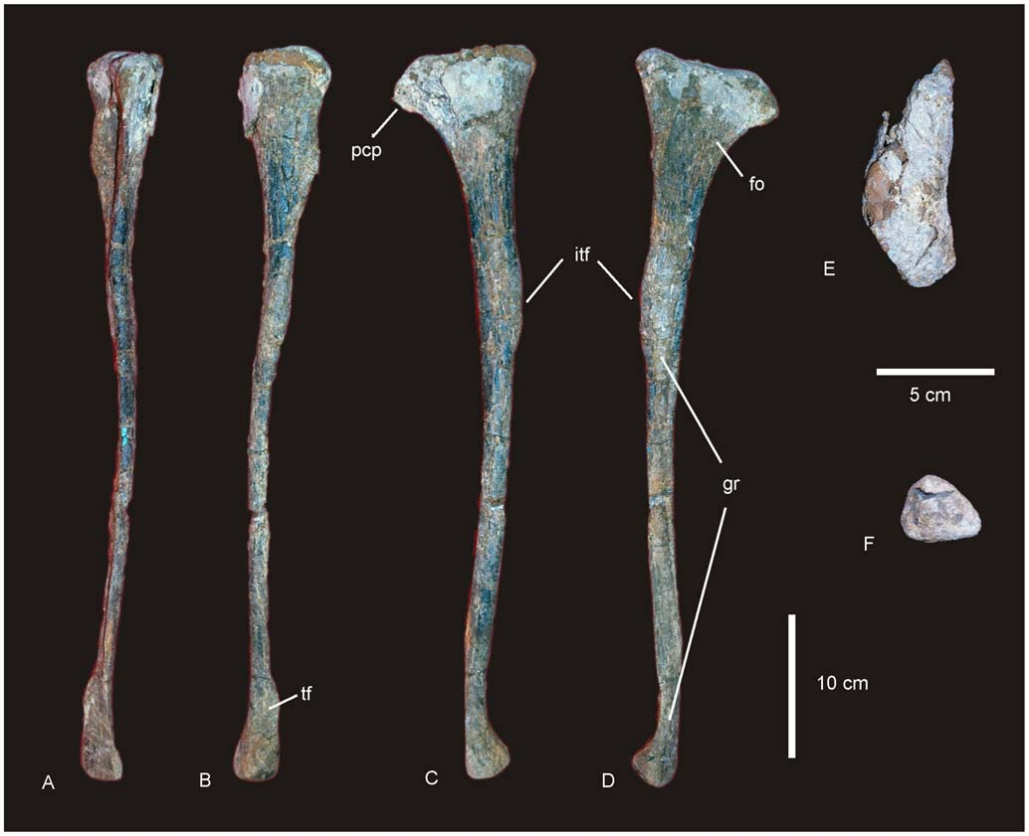
Fibula of *Australovenator wintonensis.* Right fibula in anterior (A), posterior (B), medial (C), lateral (D), proximal (E), distal (F). *Abbreviations: fo*, fossa; *gr*, groove, *itf*, attachment for *interosseum tibiofibulare*; *pcp*, proximo-cranial process; *tf*, tibial facet.

#### Astragalus (Figure 33)

In anterior view the astragalus is a broad trapezoidal bone with a tall ascending process and asymmetrical distal condyles. The medial condyle is larger than the lateral condyle and is expanded medio-laterally. The medial condyle is broad cranio-caudally and proximally possesses the medial expansion for the tibial facet, which is rounded to the same degree as the distal margin of the condyle. The lateral condyle is medio-laterally and cranio-caudally constricted so that it is only half the condylar surface of the medial condyle. This constriction reflects the lateral constriction preserved on the distal end of the tibia and tibial facet. A cranio-proximal extension of the lateral condyle projects from the cranial articular face as a rounded triangular process. This process forms the cranial boarder of the fibular facet of the astragalus. The fibular facet is shallow and bordered caudally by the lateral margin of the ascending process. In anterior view two shallow cranial grooves are present on the condylar surface. The superior cranial groove runs along the proximal margin of the condyles. The inferior cranial groove is situated in the midline of the cranial surface of the condyles. This groove is crescentic in shape and runs from the middle of the lateral condyle to middle of the medial condyle. In distal view the tibial facet is smooth along its length, being broadest medially and tapering to the lateral side. On the caudal face of the ascending process is a crescentic shallow groove which runs superior to the base of the tibial facet. Measurements in Table S24.

**Figure 33:**
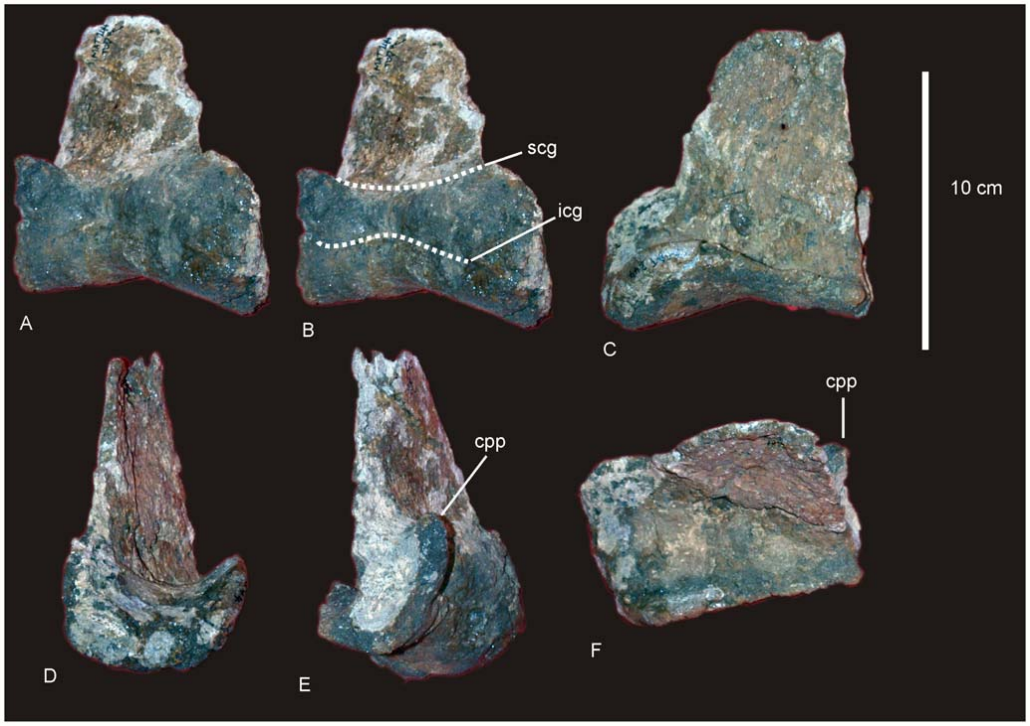
Astragalus of *Australovenator wintonensis.* Right astragalus in doral (A–B), ventral (C), medial (D), lateral (E) and proximal (F) views. Dotted lines demarcated superior and inferior cranial grooves. *Abbreviations: cpp*, cranio-proximal process; *icg*, inferior cranial groove; *spg*, superior cranial groove.

### Pes

#### Metatarsals (Figures 34 & 35)

**Figure 34:**
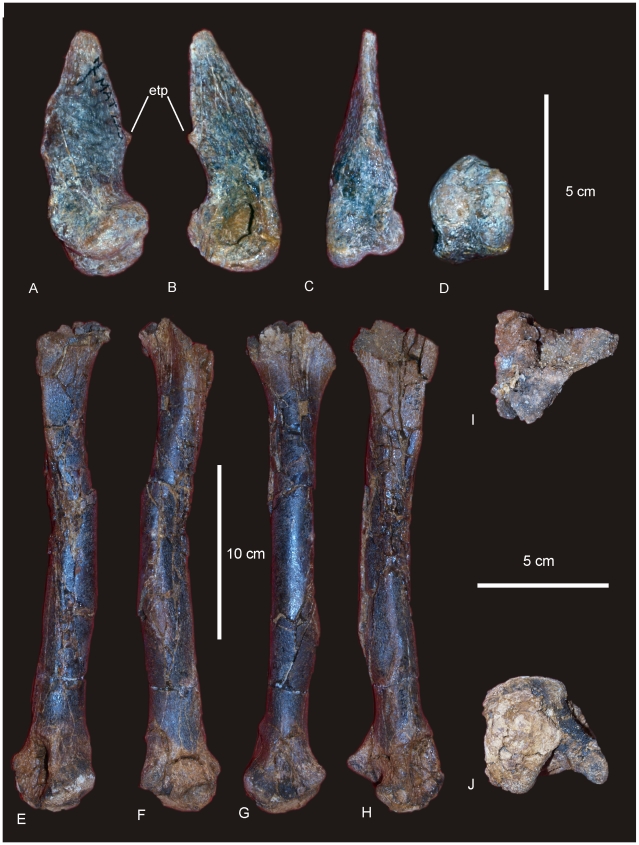
Metatarsals of *Australovenator wintonensis.* Right Mt I in dorsal (A), ventral (B), flexor (C) and distal (D) views. Right Mt III in anterior (E), posterior (F), lateral (G), medial (H), proximal (I) and distal (J) views. *Abbreviations: etp*, extensor triangular process.

Three metatarsals are preserved from the right pes, Mt I – III. The right Mt I (Figure 34A–D) is the smallest of the metatarsals and is well preserved. In flexor aspect it is tear-drop shaped. The proximal end tapers to a proximal-most point. The shaft is cranio-caudally compressed so that the medial and lateral margins form two opposing crests. Half way along the flexor surface a raised muscle scar extends to the distally expanded end. The distal condyles are asymmetrical with the cranial condyle offset ventrally to the caudal condyle which is bulbous and possesses a deep ligament pit. No deep pit occurs on the cranial side. A sharp triangular process juts out along the distal portion of the extensor crest. Measurements in Table S25.

Mt II (Figure 34E–J) is a long and gracile element preserving the distal articular condyles, and a damaged proximal epiphyseal expansion. It is missing the proximal articular surface. Proximally, the bone is subtriangular in cross-section. The cranial surface is expanded dorsally and rounded medially. The medial surface is concave and expanded proximally. The shaft is crushed and distorted so that the distal end is bent slightly medially. The distal end is expanded to accommodate a large disto-lateral condyle and a small postero-medially projecting condyle. The lateral condyle is rounded in lateral view with a deep collateral ligament pit, bordered by a distinct and thick rim. In distal view the condyle is constricted to the lateral side and expands medially as it meets the distal edge of the medial condyle. The medial and lateral condyles connect at the distal face via a bridge, which forms part of the distal articular surface. In posterior view, the lateral and medial condyles are divided by a deep fossa. In medial view the medial condyle is restricted to the lower edge of the articular surface. A deep collateral ligament pit is bordered by a thick rim. A medial muscle scar extends from the distal quarter of the caudal margin of the shaft to the lateral condyle. The proximo-lateral side of the shaft is flat and articulates with the medial face of Mt III. Measurements in Table S25.

**Figure 35:**
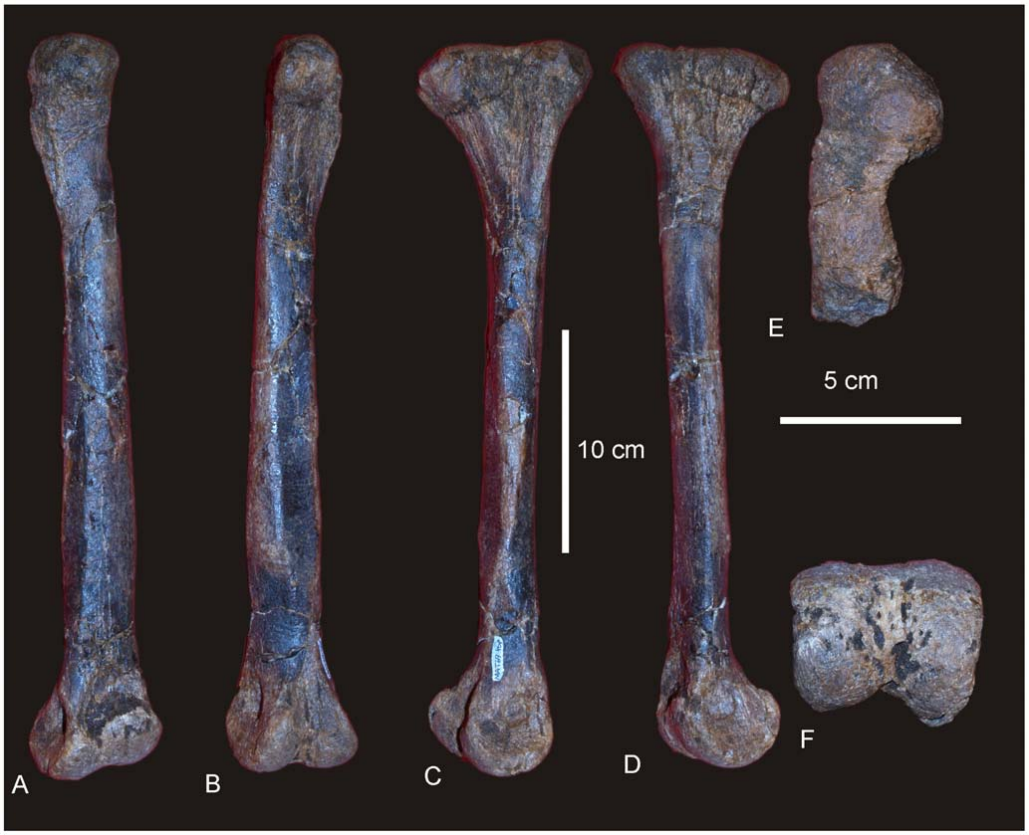
Metatarsal III of *Australovenator wintonensis.* Right Mt III in dorsal (A), ventral (B), lateral (C), medial (D), proximal (E) and distal (F) views.

Mt III (Figure 35) is the longest metatarsal, being straight and expanded cranio-caudally at the proximal end and transversely at the distal end. The metatarsal is complete, preserving the proximal and distal articular surfaces. The proximal end is broadest cranially, constricted medio-laterally and expanded slightly caudally, less so than the cranial margin. In proximal view it is subrectangular in shape with rounded proximal and distal margins. The medial face of the proximal end is broad cranio-caudally and flat. This flat face articulates with the lateral face of the Mt II. The lateral face of the proximal end possesses a cranial process which is broad proximally and tapers distally where it forms a distinct crest, which merges with the shaft a quarter of the way down the shaft. Caudal of this process, the lateral face is abruptly concave forming a shallow fossa. The caudal face of the proximal end is thick and rounded with rugosities on the bone surface. The shaft is sub-circular in cross-section with a flattened caudal face. The shaft proximally is wider cranio-caudally than transversely. Distally the mid shaft expands transversely being wider medio-laterally than cranio-caudally. The distal articular end is trapezoidal in shape with two broad and tall condyles. The medial condyle is cranio-caudally taller than the lateral condyle. The medial condyle is transversely narrower than the lateral condyle. In lateral view the lateral condyle is rounded with a deep co-lateral ligament pit which is bordered by a thick rim. In medial view, the medial condyle is similarly rounded with a deep co-lateral ligament pit. In anterior view the articular surface extends further proximally on the medial side than on the lateral side. Proximal to the articular surface is a deep transversely broad ovoid fossa. In posterior view the condyles are divided by a shallow fossa, which is bordered by low ridges that extend distally to the condyles. Measurements in Table S25.

#### Phalanges (Figure 36)

Five phalanges and two unguals are preserved. Measurements in Table S26. Right Mt III-2 is elongate with a D-shaped cross-sectional outline, with a flat caudal face. The proximal articular facet is shallowly concave with a distinctly expanded rim. The distal articular end is trapezoidal in shape with merged condyles. Both lateral and medial co-lateral ligament pits are deep and eye-shaped. In lateral and medial view the distal condyles are markedly expanded cranio-caudally. In anterior view there is a deep fossa caudal to the distal condyles.

**Figure 36:**
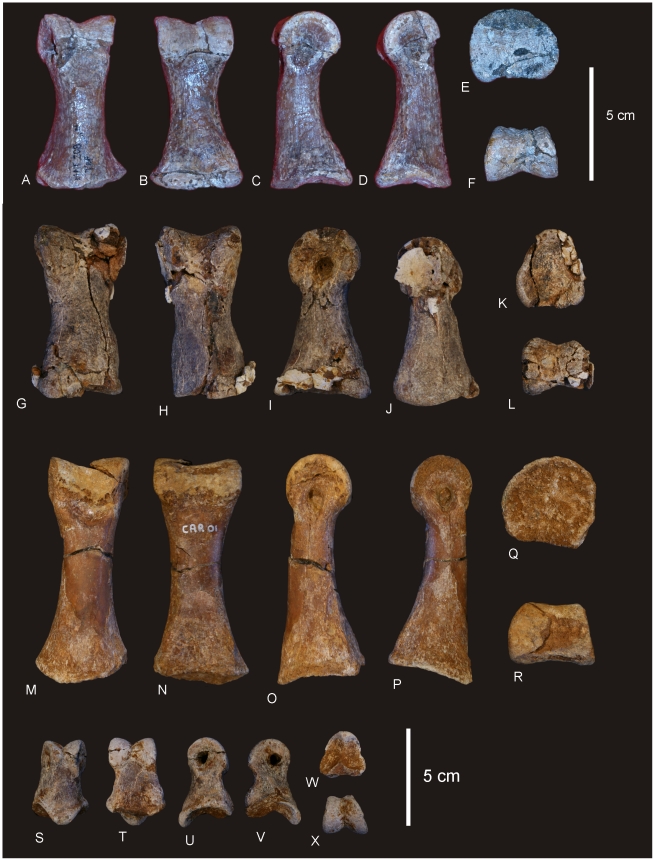
Pedal phalanges of *Australovenator wintonensis.* Phalanges in dorsal (A, G, M, S), ventral (B, H, N, T), lateral (C, I, O, U), medial (D, J, P, V), proximal (E, K, Q, W) and distal (F, L, R, X) views.

Left Mt II 1–3 were all recovered within close proximity to one another and articulate together. The first phalange is stout with a tall cranio-caudal proximal articular facet, bordered by a thick rim. The distal condyle preserves a medial and lateral condyle divided by a wide and shallow groove. The distal articular face is wider than the proximal articular face. In medial view the distal condyle possesses a deep co-lateral ligament pit. The second and third phalanges are similar to the first; however, differ by being smaller and less elongate.

#### Unguals (Figure 37)

Two pedal unguals are known from the holotype (AODF 604). Both are recurved and taper to a sharp distal point. Each is broad and tall, being oval in proximal view. Deep and distinct collateral extensor ligament grooves extend across the lateral and medial flanks of each claw.

**Figure 37:**
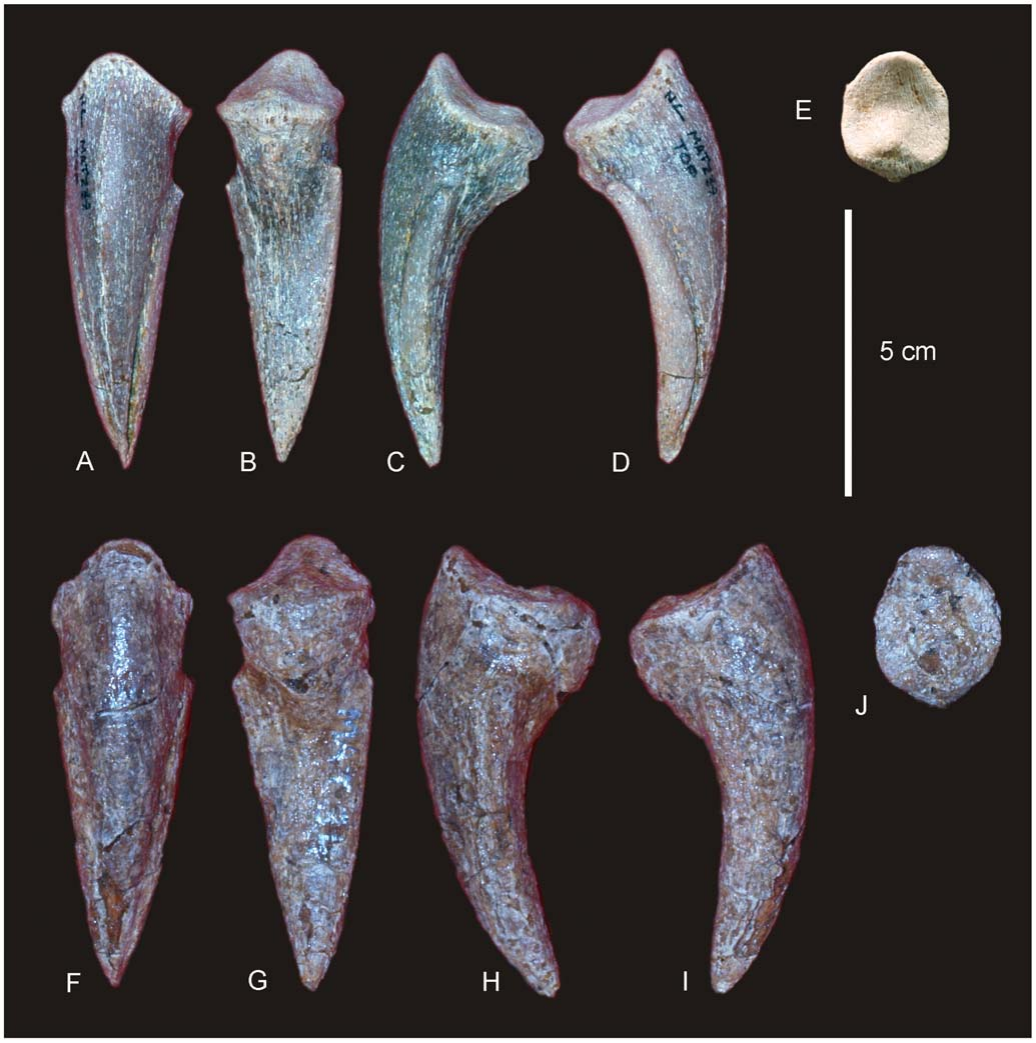
Pedal unguals of *Australovenator wintonensis.* Unguals in dorsal (A, F), ventral (B, G), lateral (C, H), medial (D, I) and proximal (E, J) views.

## Discussion

### Comparison with other Australian Cretaceous taxa

### Sauropoda

*Austrosaurus mckillopi*, from the Albian-aged Allaru Mudstone is the only Cretaceous sauropod taxon described from Australia [23]. Isolated sauropod remains from the younger late Albian-Cenomanian Winton Formation were tentatively assigned to *Austrosaurus* sp., including QMF 7292 [20]. This assignment was made tentatively because of the fragmentary preservation of the *A. mckillopi* holotype (QMF 2316). The holotype constitutes a series of approximately eight badly preserved somphospondylus dorsal vertebrae. Three specimens preserve indications of laminae, the remainder are heavily weathered central bodies. Tentative identifications of the vertebral laminae in this specimen were used to refer *Austrosaurus* to the Titanosauria [24]. No other defining features characterise this taxon within Titanosauria, other than a small number of features that can differentiate it from QMF 7292. We, therefore, consider *A. mckillopi* to be a *nomen dubium* until such time as a replacement neotype is found.

Subsequently the type locality (QML 313) of *Wintonotitan wattsi* (QMF 7292, holotype) has been thoroughly excavated and significant numbers of new bones from the specimen have been recovered, including a partial dorsal vertebra, additional caudal vertebrae, pelvic and limb elements. Furthermore, a review of the numerous fragments of the isolated bone shards collected from the surface in the 1980s and in 2006 has enabled the reconstruction of several elements, particularly portions of limb, manus and scapula. This has altered the identifications and completeness of some of the specimens identified from QMF 7292 [20] (Table 3)

**Table 3:** Changes to the identification of bone elements from QMF 7292 and additional elements recovered from the type locality in recent years (2006–2009).

Figure in [20]	Identification in [20]	New identification
PLATE I D&E	Middle caudal	Middle caudal (additional pieces added).
PLATE I P	Posterior caudal (partial)	Posterior caudal (additional pieces).
PLATE II, B&C	?Coracoid	Proximal scapula.
PLATE III, A&B	Scapular blade	Scapula blade attaches to above element.
PLATE IV A&B	Partial ulna	Additional pieces that connect proximal with distal end.
PLATE IV C	Partial ulna	Additional pieces construct the medial process.
PLATE VI A	Mc III	Mc II.
PLATE VI F&G	Mc II	Mc V (proximal end).
PLATE VI H	Mc II	Mc IV (distal end).
PLATE VI J & K	Mc II	Mc I (proximal end).
PLATE VI R & S	?Carpal	Mc V (distal end).
Additional elements recovered in recent years.		
		Partial humerus
		Partial sacral vertebra.
		Partial ilium.
		Ischium.
		Partial dorsal vertebra
		Partial neural spines
		Additional caudal vertebrae
		Chevrons

The only specimen from QMF 7292 directly comparable to the *A. mckillopi* holotype is a partial dorsal centrum. The vertebra clearly differs from QMF 2316 dorsal vertebra specimen ‘B’, by being much more elongate cranio-caudally and by possessing a much larger, caudally acuminate (‘eye-shaped’) pleurocoel. It differs from QMF 2316 specimen ‘A’ and the remaining dorsal vertebral series by having a more acuminate caudal margin of the pleurocoel; a rounded latero-ventral profile to the centrum; a more elliptical caudal articular face (dorso-ventrally compressed) and a more cranially oriented and rounded ventral origin of the caudal centrodiapophyseal lamina.

The only other sauropod taxa represented from the Australian Cretaceous are the two new taxa described here, *Diamantinasaurus matildae* and *W. wattsi*. *D. matildae* differs from *W. wattsi* in most features of the skeleton where similar elements are preserved.

#### Scapula

The scapular blade of *D. matildae* is flat and rectangular in cross-section, possessing a medio-laterally thick glenoid fossa; compared to a distinct scapular ridge with a constricted glenoid fossa and pronounced acromial ridge in *W. wattsi*.

#### Humerus

The humerus of *D. matildae* is proximally broad, stout and possesses undivided and flat condyles; compared to the proximally narrow, elongate humerus with divided condyles seen in *W. wattsi*.

Ulna. The ulna of *D. matildae* is massively broad proximally and stout; compared with a more elongate element in *W. wattsi*.

#### Metacarpals

Metacarpal formula 2-1-1-1-0 with Mc III longest in *D. matildae*; compared to metacarpal formula 0-0-0-0-0 with Mc I longest in *W. wattsi*.

Dorsal ribs. The dorsal ribs of *D. matildae* are only pneumatic in the proximal expansion of the rib, whereas the pneumatic cavities in *W. wattsi* extend a third of the way distally.

#### Pelvis

The pelvic girdle of *D. matildae* is massive and rotund with a relatively small ischium, which is fused along its symphysis with its counterpart; compared to *W. wattsi*, which has a relatively small ilium and large blade-like ischium which is broad along its entire length.

### Theropoda

The Australian theropod fossil record is exceptionally poor with all previously described taxa based on one or two fragmentary individual skeletal elements. Four theropod taxa have been formally described from Australia with two additional taxa referred to taxa from elsewhere; *Walgettosuchus woodwardi* Huene; *Rapator ornitholestoides* Huene; *Kakuru kujani* Molnar & Pledge; *Timimus hermani* Rich & Vickers Rich; *Allosaurus* sp. Molnar et al.; and cf. *Megaraptor* Smith et al.. Both *W. woodwardi* [25] and *T. hermani* [26] are considered to be *nomen dubia*.

The Mc I of *Australovenator wintonensis* is larger than to that of the *Rapator ornitholestoides* holotype; is missing the prominent caudo-medial process projecting proximo-dorsally; possesses more subequal distal condyles; a flat proximal articular surface compared to the concave surface in *R. ornitholestoides*; and a straight distal lateral condyle compared to a disto-laterally projecting distal lateral condyle in *R. ornitholestoides*. *Rapator ornitholestoides* has been considered to represent an alvarezsaurid coelurosaur [25], however it's identification remains equivocal.

An ulna from the Early Cretaceous of southern Australia has been compared to *Megaraptor* [27]. The ulnae of *A. wintonensis* differ from NMVP186076 (cf. *Megaraptor*) in several features: 1. It lacks a proximo-caudally expanded blade-like olecranon process that extends distally as a caudal olecranon crest. 2. It lacks a pronounced lateral tuberosity that is continuous distally with a distinct lateral crest. 3. The caudal margin of the ulna is relatively straight, not as bowed caudally as in NMVP186076. 4. The ulnae possess a lateral groove along the dorso-lateral surface of the diaphysis, which is lacking in NMVP 186076 and *Megaraptor*. 5. The distal end is transversely wider, possessing a greater medial expansion.

An isolated astragalus from the Early Cretaceous of southern Australia has been assigned to *Allosaurus* sp. [28]. Several authors have debated the taxonomic affinities of this specimen in the intervening decades, with its referral to the Ornithomimosauria [29]; reiterated as *Allosaurus* in response [30]; and in more recent years considered to be related to the basal allosauroid *Fukuiraptor* [31]; or to have abelisauroid affinities [32], [33].

On comparison of the astragalus of the holotype specimen of *A. wintonensis* (AODF 604) with that of *Allosaurus* sp. (NMVP 150070) and that of *Fukuiraptor kitadaniensis* (FPMN 9712221), some striking similarities are observed. All three specimens are close in size and overall proportions. In anterior view, the three specimens share an enlarged medial condyle, which is expanded medially and cranio-caudally (medial margin not preserved in *Fukuiraptor*); along with a very tall and quadrangular-shaped ascending process (broken in MNVP 150070). All specimens possess a superior cranial groove that runs along the base of the ascending process and an inferior cranial groove which runs from the lateral-most edge of the middle of the cranial face to the middle of the cranial face of the medial condyle (‘Upper horizontal groove’ and ‘lower horizontal groove’ in [28]). Each also possess a cranio-proximal extension of the lateral condyle which projects from the cranial articular face as a rounded triangular process; damaged in NMVP 150070 and small in *Fukuiraptor*. In posterior view, each possesses a crescentic groove that runs from the middle of the ascending process toward the tibial facet, badly preserved in AODF 604. The similarities shared amongst these three specimens, are also those that differ them from other allosauroids, such as *Sinraptor* and *Allosaurus*. We suggest that all three astragali represent very similar allosauroids and that NMVP 150070 is closest in morphology to *Australovenator*.

### Phylogenetic Discussion

**Figure 38:**
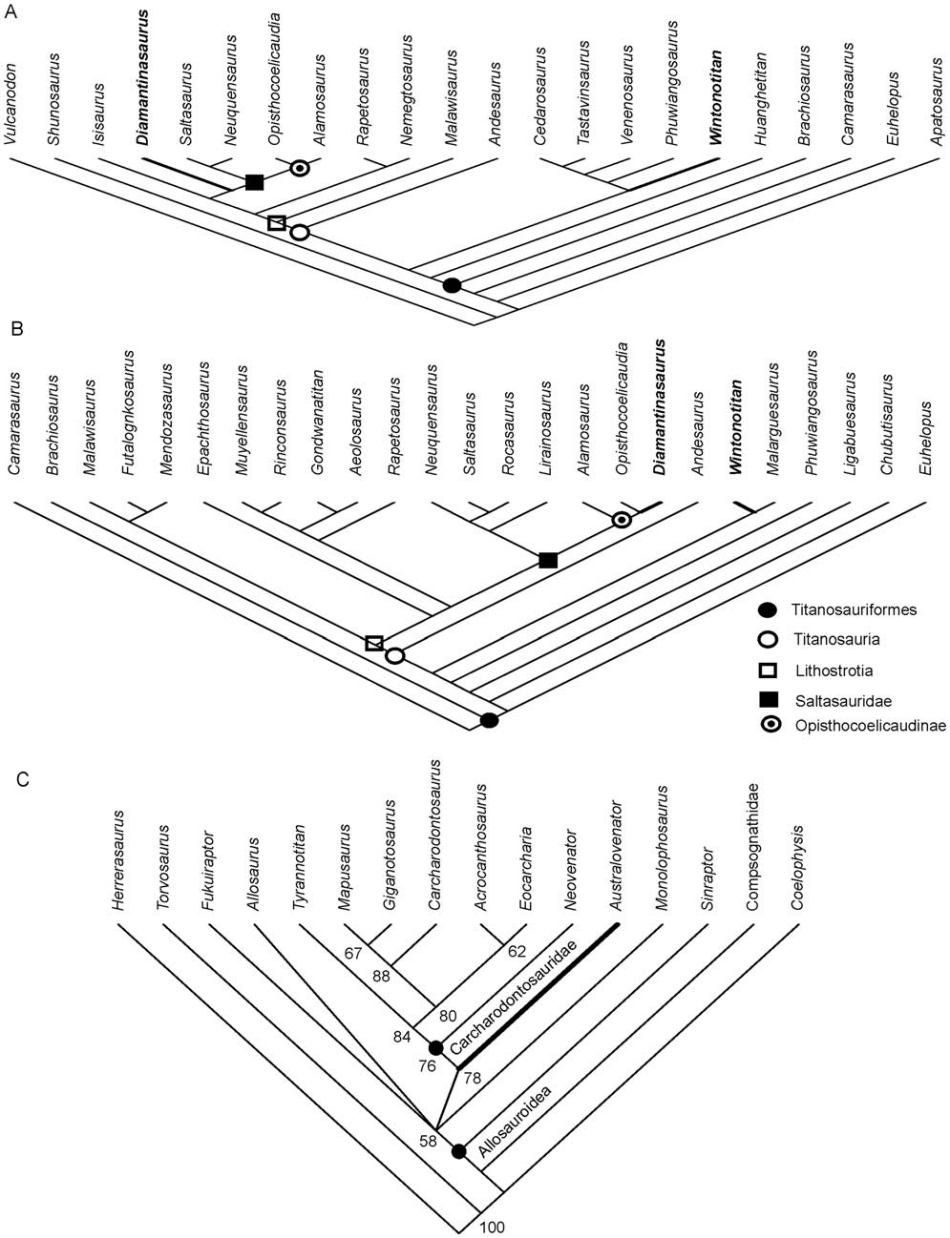
Phylogenetic position of the three new dinosaurs. A–B. *Diamantinasaurus matildae* and *Wintonotitan wattsi*. Most-parsimonious tree (MPT) from first analysis (tree length (TL) = 325 steps; consistency index (CI) = 0.64; retention index (RI) = 0.99) (A) and MPT from the second analysis (TL = 220; CI = 0.58; RI = 0.93) (B). C. Phylogenetic position of *Australovenator wintonensis*. Strict consensus tree of 5 MPTs (TL = 181; CI = 0.59; RI = 0.91). Bootstrap values provided at the nodes (>50%).

Phylogenetic position of *Diamantinasaurus* and *Wintonotitan* (Figure 38A–C).

Previous studies of titanosaur relationships have proposed lists of synapomorphies for defined clades [15]. *Diamantinasaurus matildae* is considered a titanosauriform by possessing the following synapomorphies from respective authorities: broadly expanded preacetabular lobe of the ilium; pubic peduncle perpendicular to sacral axis; prominent lateral bulge in femur distal to greater trochanter [34]. Scapula glenoid deflected medially; dorsal ribs with pneumatic cavities; metacarpal I with distal condyle undivided with a reduced articular surface [35]. Anterior dorsal ribs plank-like; Mc I distal condyle oriented perpendicular to axis of shaft; iliac preacetabular process semi-circular [36]. Proximal one third of femur shaft deflected medially [15].

It is considered a member of the Titanosauria by possessing the following synapomorphies: pubis considerably longer than ischium [34]. Crescentic sternal plates; olecranon process of ulna prominent and extended distally; ischial blade plate-like; distal tibia expanded transversely to twice mid-shaft width [36].

Finally, it can be considered a derived lithostrotian titanosaur by possessing the following synapomorphies found in ‘Titanosauridae’ [34] and Saltasauridae [36]: preacetabular blade projecting outwards (perpendicular) from main axis of sacrum; relatively short posterior process of ischium [34]. Scapular blade forming a 45° angle with coracoid articulation; humeral distal condyles exposed on anterior of shaft; stout ulna; humeral deltopectoral crest markedly expanded distally; elliptical femoral mid-shaft cross-section; distal condyles of femur bevelled dorso-medially; astragalar posterior fossa undivided [36].

*Diamantinasaurus matildae* can be differentiated from other derived lithostrotian titanosaurs, such as *Opisthocoelicaudia, Alamosaurus, Saltasaurus* and *Nequenosaurus* by retaining metacarpal phalangeal facets and phalanges, with Mc III.1 heavily reduced; the presence of a manus ungual claw; flat distal humeral condyles and subequal distal femoral condyles.

*Wintonotitan wattsi* is considered to be a somphospondylus titanosauriform by possessing the following synapomorphies from respective authorities: presence of prespinal laminae on dorsal vertebra neural spines; neural arches positioned anteriorly in mid and posterior caudal centra; broadly expanded and upwardly directed preacetabular lobe of ilium; pubic peduncle perpendicular to sacral axis [34]. Spongy somphospondylus presacral vertebrae; dorsal ribs plank-like with pneumatic cavities; iliac preacetabular process semicircular; scapular glenoid fossa bevelled medially [36]. *W. wattsi* may be a member of the Titanosauria by possessing the following synapomorphies: eye-shaped pleurocoels in dorsal vertebrae [34]. Prominent olecranon process; ischial blade plate-like [36].

*Wintonotitan wattsi* can be differentiated from other basal titanosauriformes, such as *Phuwiangosaurus*, *Venenosaurus*, *Tastivanosaurus*, *Cedarosaurus*, *Malarguesaurus* and *Andesaurus* by possessing incipiently biconvex distal caudal vertebrae; anterior caudal neural arches with prespinal laminae and anterior and middle caudal vertebrae with ventral longitudinal hollows.

Recent titanosaur phylogenetic analyses were used to determine the phylogenetic position of *D. matildae* and *W. wattsi* [47], [48]. These two analyses were chosen because they independently analyse a large number of basal and derived titanosaurs with two differing character sets and terminal taxa. We added *Huanghetitan*, *Diamantinasaurus* and *Wintonotitan* to the first analysis [37] and retained *Alamosaurus* and *Nemegtosaurus* in the final ingroup list (Cladistic Matrix S1). A single most-parsimonious tree was returned (TL = 325 steps; CI = 0.64; RI = 0.99). The second analysis [38] added *Diamantinasaurus* and *Wintonotitan* (Cladistic Matrix S2). A single most-parsimonious tree was returned (TL = 220 steps; CI = 0.58; RI = 0.93) for this analysis. Both analyses were bootstrapped, returning low values (<50%) for all derived nodes.

In both most-parsimonious trees *Wintonotitan* was resolved as a basal titanosauriform and *Diamantinasaurus* as a derived lithostrotian titanosaur. The position of *Wintonotitan* in both most-parsimonious trees is relatively similar within the basal titanosauriforms; being basal to *Andersaurus* and close to *Phuwiangosaurus*. In the first analysis *Diamantinsaurus* was returned as the sister taxon to the Saltasauridae, however, the second analysis placed it as the sister taxon to *Opisthocoelicaudia* as an opisthocoelicaudine.

Based on these two separate analyses we are confident in the higher level placement of *Wintonotitan* as a basal titanosauriform and *Diamantinasaurus* as a lithostrotian titanosaur. Based on the synapomorphies provided by others, *Wintonotitan* may also be considered in the Titanosauria and *Diamantinasaurus* within the Saltasauridae.

Due to the low bootstrap support for these analyses, further speculation on their relationships will await a more detailed analysis of titanosaur generic-level relationships. By increasing the number of tree steps by 2 in both analyses, each clade containing *Wintonotitan* and *Diamantinasaurus* collapses into an unresolved polytomy. The results of these analyses simply reflect the poor resolution available in titanosaur phylogenetics at the present time, primarily based on large amounts of missing data [15].

### Phylogenetic position of *Australovenator wintonensis* (Figure 38C, Cladistic Matrix S3)

Using previous studies of allosauroid relationships, *Australovenator wintonensis* possesses the following unambiguous synapomorphies for the *Allosaurus* + Carcharodontosauridae clade [18]; slightly forked posterior end of the dentary; pronounced medial epicondyle (mediodistal crest), extending 30% length of the femur; conspicuous narrowing between the lateral condyle and the main body of the tibia. *Australovenator* possesses an intermediate trait between *Allosaurus* and carcharodontosaurids; tibia lateral malleolus distal extension relative to medial malleolus: extends 5% of the length of the tibia, versus 7% or more in Carcharodontosauridae. Finally, *Australovenator* is considered to be a derived allosauroid possessing a single unambiguous synapomorphy of the Carcharodontosauridae clade [18]; femur head angled dorsally, resulting in an obtuse angle between the head and the shaft.

*Australovenator* differs from *Neovenator* and derived carcharodontosaurids by the following characteristics; 1. Dentary with a posterior neurovascular foramina row that does not deflect ventrally. 2. A femur with a reduced cranial process on the lesser trochanter; less developed medial distal crest (epicondyle) and a lesser trochanter extended proximally to be level with the greater trochanter. 3. A fibula with a proximal end which is cranially bevelled and a triangular cranio-distal process that projects from the proximal end. 4. A tibia with a medio-laterally wider proximal end and a lateral malleolus that does not extend as far distally. 5. Elongate and gracile metatarsals with Mt III bearing a medio-laterally compressed proximal articular end.

*Australovenator* differs from *Fukuiraptor* by the following characteristics: 1. Straight caudal margin of ulna; slightly bowed cranial margin; mid-shaft narrower. 2. Femur head projecting medio-dorsally; circular caput; narrow distal condyles and a reduced epicondyle. 3. Tibia with a ventral spine-like process extending from the cranio-lateral process and a squared-off cnemial crest. 4. Mt I broader with a longitudinal muscle scar; Mt II distal condyles divergent; Mt III sub-rectangular proximal end.

In order to determine the phylogenetic position of *Australovenator wintonensis* we decided to add *Australovenator* to the data matrix used in the most recent cladistic analysis of allosauroid relationships [18]. Characters and character state scores were all maintained within our analysis with the only addition being the character state scores for *Australovenator* (Supporting Information).

Our analysis returned five most-parsimonious trees with a tree length of 181 steps; a consistency index of 0.59 and a retention index of 0.91 (Figure 38C). All five trees returned a similar position for *Australovenator*, as the sister taxon to the Carcharodontosauridae [18] with a well-supported bootstrap value of 78%. Resolution within the carcharodontosaurids remained the same as the previous analysis [18] with *Neovenator* remaining the basal-most taxon and high bootstrap values supporting the carcharodontosaurid clades.

We retained *Fukuiraptor* and *Monolophosaurus* in our analysis in order to see whether there were close relationships of these basal allosauroid taxa with *Australovenator* [18]. Our analysis returned a similar topology to that previous analyses when *Fukuiraptor* and *Monolophosaurus* were retained [18]. Previous analysis has returned a polytomy with *Allosaurus*, *Fukuiraptor, Monolophosaurus* and carcharodontosaurids more derived than *Sinraptor* [18]. Our analysis returned a similar polytomy, with the clade containing *Australovenator* and carcharodontosaurids forming a polytomy with *Allosaurus*, *Fukuiraptor* and *Monolophosaurus*. This polytomy returned bootstrap support greater than 50%, however, still low at 58%. By increasing the number of tree steps by 1 in the analysis, *Australovenator* collapses into a basal polytomy with *Neovenator*, *Allosaurus*, *Monolophosaurus* and *Fukuiraptor*. This result reflects the poor resolution available in allosauroid phylogenetic studies and large amounts of missing data [18], [25], [31]–[33].

### Palaeobiogeographic Implications

Reconstructing the palaeobiogeographic and phylogeographic history of organisms is only as good as the phylogenetic robustness of the group under study and the resolution that the fossil record provides on past distributions of these taxa. This is especially true for the understanding of titanosauriform sauropods and their descendants [39], [40]. Although the phylogenetic analyses of the Winton sauropods returned poor statistical support, the consistent placement of *Wintonotitan* as a basal titanosauriform and *Diamantinasaurus* as a derived lithostrotian titanosaur illustrates the diversity of Cretaceous sauropods in Australia as similar to that found on any other continent.

**Figure 39:**
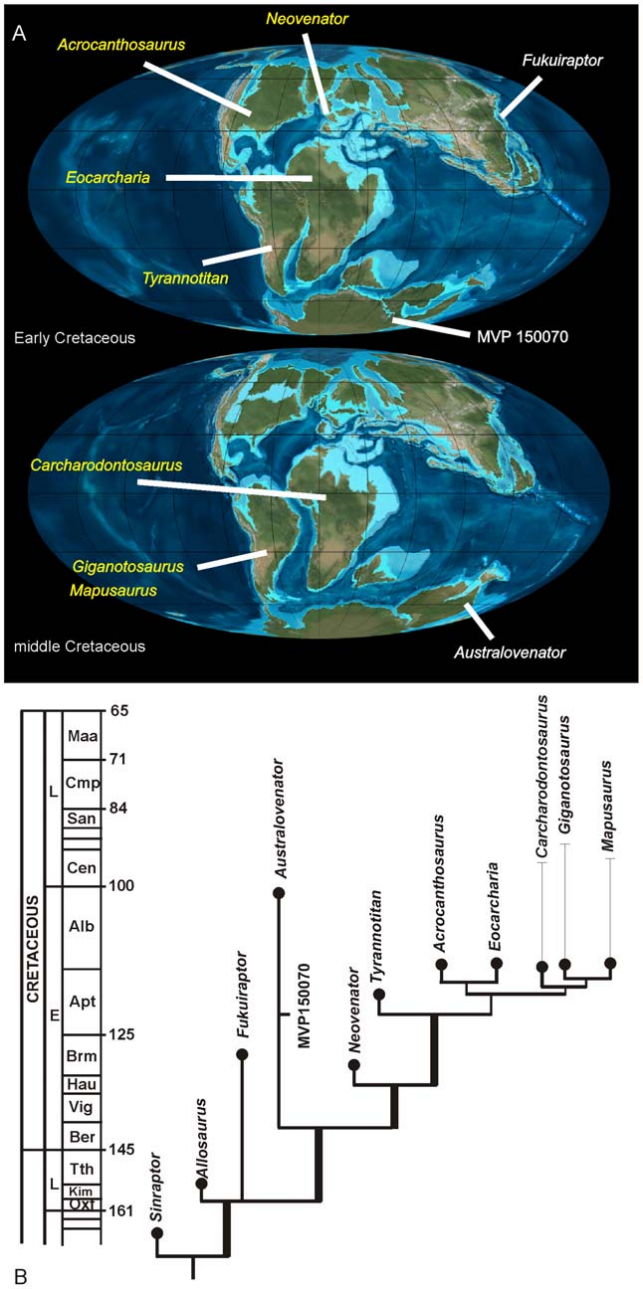
Palaeobiogeographic map and strato-phylogeny of *Australovenator wintonensis.* A. Palaeogeographic globe for the Early and middle Cretaceous [51]. White-lettered taxa represent allosauroids basal to the Carcharodontosauridae. Yellow-lettered taxa represent members of the Carcharodontosauridae. B. Stratigraphically calibrated phylogeny based on the phylogeny of the current analysis and adapted from [18].

**Figure 40:**
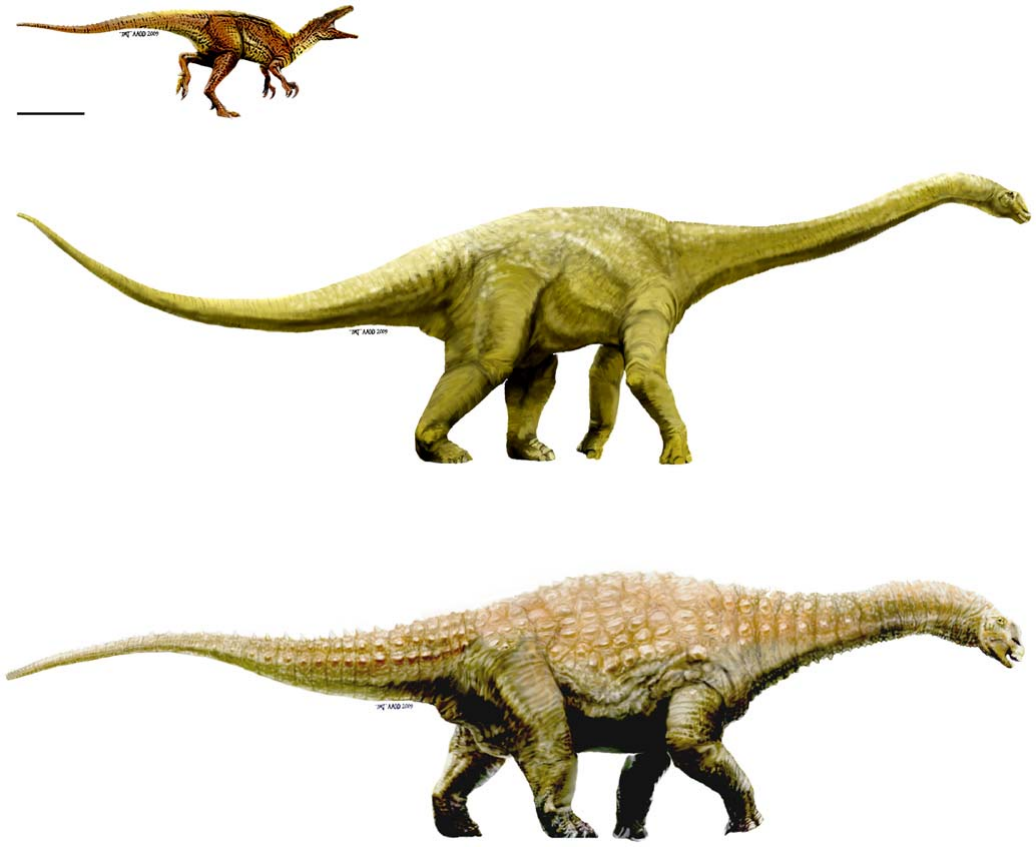
Reconstructions of *Diamantinasaurus matildae, Wintonotitan wattsi, and Australovenator wintonensis.* Artistic representations of the three dinosaur taxa described here. *Australovenator* (top); *Wintonotitan* (middle); *Diamantinasaurus* (bottom). Artwork by: T. Tischler, Australian Age of Dinosaurs Museum of Natural History.

The analysis of *Australovenator* provides a more robust result. The basal position of *Australovenator* to the Carcharodontosauridae; the close morphological similarities to the more plesiomorphic allosauroid *Fukuiraptor* from Japan; and the close similarities to the derived *Neovenator* (a basal carcharodontosaurid) from Europe, implies a near global distribution for allosauroids basal to the Carcharodontosauridae (Figure 39A). The Aptian-late Albian age for *Australovenator* is younger than *Neovenator* and *Fukuiraptor* and similar in age to the more derived carcharodontosaurines on other continents; however, there is yet to be a confirmed presence of carcharodontosaurids in Australia (Figure 39B). These new taxa, along with the fragmentary remains from other taxa (e.g. *Rapator* and cf. *Megaraptor*), indicate a diverse Early Cretaceous sauropod and theropod fauna in Australia, including plesiomorphic forms (e.g. *Wintonotitan* and *Australovenator*) and more derived forms (e.g. *Diamantinasaurus*) (Figure 40).

## Supporting Information

Cladistic Matrix S1 (0.11 MB XLS)

Cladistic Matrix S2 (0.05 MB XLS)

Cladistic Matrix S3 (0.03 MB XLS)

Table S1 *Diamantinasaurus matildae* - Dorsal rib measurements (mm)
(0.03 MB DOC)

Table S2 *Diamantinasaurus matildae* - Scapula measurements (mm)
(0.02 MB DOC)

Table S3 *Diamantinasaurus matildae* - Sternal plate measurements (mm)
(0.03 MB DOC)

Table S4 *Diamantinasaurus matildae* - Humerus measurements (mm)
(0.03 MB DOC)

Table S5 *Diamantinasaurus matildae* - Ulna measurements (mm)
(0.04 MB DOC)

Table S6 *Diamantinasaurus matildae* - Manus measurements (mm)
(0.03 MB DOC)

Table S7 *Diamantinasaurus matildae* - Pelvic girdle measurements (mm)
(0.03 MB DOC)

Table S8 *Diamantinasaurus matildae* - Femur measurements (mm)
(0.03 MB DOC)

Table S9 *Diamantinasaurus matildae* - Tibia measurements (mm)
(0.03 MB DOC)

Table S10 *Diamantinasaurus matildae* - Fibula measurements (mm)
(0.03 MB DOC)

Table S11 *Wintonotitan wattsi* - Vertebral measurements (mm)
(0.05 MB DOC)

Table S12 *Wintonotitan wattsi* - Chevron measurements (mm)
(0.03 MB DOC)

Table S13 *Wintonotitan wattsi* - Forearm measurements (mm)
(0.03 MB DOC)

Table S14 *Wintonotitan wattsi* - Metacarpal measurements (mm)
(0.03 MB DOC)

Table S15 *Wintonotitan wattsi* - Ilium and Ischium measurements (mm)
(0.03 MB DOC)

Table S16 *Australovenator wintonensis* - Tooth measurements (mm)
(0.31 MB DOC)

Table S17 *Australovenator wintonensis* - Dentary measurements (mm)
(0.03 MB DOC)

Table S18 *Australovenator wintonensis* - Dorsal rib measurements (mm)
(0.03 MB DOC)

Table S19 *Australovenator wintonensis* - Ilium measurements (mm)
(0.03 MB DOC)

Table S20 *Australovenator wintonensis* - Forearm measurements (mm)
(0.04 MB DOC)

Table S21 *Australovenator wintonensis* - Femur measurements
(0.03 MB DOC)

Table S22 *Australovenator wintonensis* - Tibia measurements
(0.03 MB DOC)

Table S23 *Australovenator wintonensis* - Fibula measurements (mm)
(0.03 MB DOC)

Table S24 *Australovenator wintonensis* - Astragalus measurements (mm)
(0.03 MB DOC)

Table S25 *Australovenator wintonensis* - Metatarsal measurements (mm)
(0.03 MB DOC)

Table S26 *Australovenator wintonensis* - Pes phalange and ungual measurements (mm)
(0.03 MB DOC)
