# Supplementary material for: New Mid-Cretaceous (Latest Albian) Dinosaurs from Winton, Queensland, Australia
Source: PLoS One. 2009 Jul 3;4(7):e6190. doi: 10.1371/journal.pone.0006190 (PMC2703565; doi:10.1371/journal.pone.0006190)
Supplement: Table S1 — Diamantinasaurus matildae - Dorsal rib measurements (mm) (0.03 MB DOC) [file pone.0006190.s004.doc]

***Diamantinasaurus matildae***

Table S 1. Dorsal rib measurements (mm)

|  | Length |
| --- | --- |
| Anterior Dorsal Rib | 1650 |
| Posterior Dorsal Rib | 1350 |
