# Supplementary material for: New Mid-Cretaceous (Latest Albian) Dinosaurs from Winton, Queensland, Australia
Source: PLoS One. 2009 Jul 3;4(7):e6190. doi: 10.1371/journal.pone.0006190 (PMC2703565; doi:10.1371/journal.pone.0006190)
Supplement: Table S2 — Diamantinasaurus matildae - Scapula measurements (mm) (0.02 MB DOC) [file pone.0006190.s005.doc]

***Diamantinasaurus matildae***

Table S 2. Scapula measurements (mm)

| Scapula Length | 1400 |
| --- | --- |
| Scapula blade width |  |
