# Supplementary material for: New Mid-Cretaceous (Latest Albian) Dinosaurs from Winton, Queensland, Australia
Source: PLoS One. 2009 Jul 3;4(7):e6190. doi: 10.1371/journal.pone.0006190 (PMC2703565; doi:10.1371/journal.pone.0006190)
Supplement: Table S3 — Diamantinasaurus matildae - Sternal plate measurements (mm) (0.03 MB DOC) [file pone.0006190.s006.doc]

***Diamantinasaurus matildae***

Table S 3. Sternal plate measurements (mm)

| Sternal plate |  |
| --- | --- |
| Length | 470 |
| Maximum width | 230 |
