# Supplementary material for: New Mid-Cretaceous (Latest Albian) Dinosaurs from Winton, Queensland, Australia
Source: PLoS One. 2009 Jul 3;4(7):e6190. doi: 10.1371/journal.pone.0006190 (PMC2703565; doi:10.1371/journal.pone.0006190)
Supplement: Table S4 — Diamantinasaurus matildae - Humerus measurements (mm) (0.03 MB DOC) [file pone.0006190.s007.doc]

***Diamantinasaurus matildae***

Table S 4. Humerus measurements (mm)

|  | Length | Proximal Width | Distal Width | Mid-shaft Width | Mid-shaft Depth |
| --- | --- | --- | --- | --- | --- |
| Left Humerus | 1100 | 512.50 | 300+ | 180 | 230 |
| Right Humerus | 1120 | 450+ | 420 | 230 | 150 |
