# Supplementary material for: New Mid-Cretaceous (Latest Albian) Dinosaurs from Winton, Queensland, Australia
Source: PLoS One. 2009 Jul 3;4(7):e6190. doi: 10.1371/journal.pone.0006190 (PMC2703565; doi:10.1371/journal.pone.0006190)
Supplement: Table S5 — Diamantinasaurus matildae - Ulna measurements (mm) (0.04 MB DOC) [file pone.0006190.s008.doc]

***Diamantinasaurus matildae***

Table S 5. Ulna measurements (mm)

| Ulna |  |
| --- | --- |
| Length along craniomedial crest | 630 |
| Length along caudal edge to olecranon | 710 |
| Length from olecranon to craniodistal margin | 730 |
| Proximal width from craniomedial crest to olecranon | 390 |
| Proximal width just distal to epiphysis | 350 |
| Mid-shaft width | 130 |
| Craniomedial – olecranon process | 340 |
| Olecranon – lateral process | 350 |
| Lateral – craniomedial process | 300 |
| Width of craniomedial process | 150 |
| Width of olecranon process | 100 |
| Width of lateral process | 90 |
| Distal width 1 | 190 |
| Distal width 2 | 150 |
