# Supplementary material for: New Mid-Cretaceous (Latest Albian) Dinosaurs from Winton, Queensland, Australia
Source: PLoS One. 2009 Jul 3;4(7):e6190. doi: 10.1371/journal.pone.0006190 (PMC2703565; doi:10.1371/journal.pone.0006190)
Supplement: Table S6 — Diamantinasaurus matildae - Manus measurements (mm) (0.03 MB DOC) [file pone.0006190.s009.doc]

***Diamantinasaurus matildae***

Table S 6. Manus measurements (mm)

| Metacarpal | Length | Proximal Width | Distal Width | Mid-shaft width |
| --- | --- | --- | --- | --- |
| Mc I | 355 | 172  129 | 153  124 | 105 |
| Mc II | 400 | 150  120 | 150  120 | 86 |
| Mc III | 410 | 170  140 | 173 | 92 |
| Mc IV | 370 | 160  110 | 162 | 85 |
| Mc V | 330 | 130  100 | 140 | 72 |

|  | Length | Posterior height |
| --- | --- | --- |
| Manus Claw | 170.99 | 67.19 |
