# Supplementary material for: New Mid-Cretaceous (Latest Albian) Dinosaurs from Winton, Queensland, Australia
Source: PLoS One. 2009 Jul 3;4(7):e6190. doi: 10.1371/journal.pone.0006190 (PMC2703565; doi:10.1371/journal.pone.0006190)
Supplement: Table S7 — Diamantinasaurus matildae - Pelvic girdle measurements (mm) (0.03 MB DOC) [file pone.0006190.s010.doc]

***Diamantinasaurus matildae***

Table S 7. Pelvic girdle measurements (mm)

|  | Length | Preacetabular process height | Acetabular length |
| --- | --- | --- | --- |
| Ilium | 87 | 47 | 37 |

|  | Length | Proximal Width | Distal Width |
| --- | --- | --- | --- |
| Right Pubis | 1070 | 370 | 390 |
| Left Pubis | 1100 | 480 | 290 |

| Right ischium maximum length | 540 |
| --- | --- |
| Pubio-ischial contact | 420 |
| Acetabular length | 380 |
| Mid-shaft width | 210 |
| Left Ischium proximal portion Length | 400+ |
| Proximal portion width | 300+ |
