# Supplementary material for: New Mid-Cretaceous (Latest Albian) Dinosaurs from Winton, Queensland, Australia
Source: PLoS One. 2009 Jul 3;4(7):e6190. doi: 10.1371/journal.pone.0006190 (PMC2703565; doi:10.1371/journal.pone.0006190)
Supplement: Table S8 — Diamantinasaurus matildae - Femur measurements (mm) (0.03 MB DOC) [file pone.0006190.s011.doc]

***Diamantinasaurus matildae***

Table S 8. Femur measurements (mm)

| Femur |  |
| --- | --- |
| Length | 1330 |
| Mid-shaft width | 262 |
| Mid-shaft depth | 120 |
| Proximal width | 43 |
| Proximal depth | 20 |
| Distal width | 52 |
| Medial condyle height | 23 |
| Medial condyle width | 26 |
| Lateral condyle height | 20 |
| Lateral condyle width | 26 |
