# Supplementary material for: New Mid-Cretaceous (Latest Albian) Dinosaurs from Winton, Queensland, Australia
Source: PLoS One. 2009 Jul 3;4(7):e6190. doi: 10.1371/journal.pone.0006190 (PMC2703565; doi:10.1371/journal.pone.0006190)
Supplement: Table S9 — Diamantinasaurus matildae - Tibia measurements (mm) (0.03 MB DOC) [file pone.0006190.s012.doc]

***Diamantinasaurus matildae***

Table S 9. Tibia measurements (mm)

| Tibia |  |
| --- | --- |
| Length | 80 |
| Proximal width 1 | 30 |
| Proximal width 2 | 29 |
| Distal width 1 | 32 |
| Distal width 2 | 17 |
