# Supplementary material for: New Mid-Cretaceous (Latest Albian) Dinosaurs from Winton, Queensland, Australia
Source: PLoS One. 2009 Jul 3;4(7):e6190. doi: 10.1371/journal.pone.0006190 (PMC2703565; doi:10.1371/journal.pone.0006190)
Supplement: Table S10 — Diamantinasaurus matildae - Fibula measurements (mm) (0.03 MB DOC) [file pone.0006190.s013.doc]

***Diamantinasaurus matildae***

Table S 10. Fibula measurements (mm)

| Fibula |  |
| --- | --- |
| Length | 17+32+22 = 71+ |
| Mid-shaft width | 15 |
| Proximal width 1 | 23.4 |
| Proximal width 2 | 13.5 |
| Proximal width 3 | 9 |
| Distal width 1 | 18.5 |
| Distal width 2 | 18.2 |
