# Supplementary material for: New Mid-Cretaceous (Latest Albian) Dinosaurs from Winton, Queensland, Australia
Source: PLoS One. 2009 Jul 3;4(7):e6190. doi: 10.1371/journal.pone.0006190 (PMC2703565; doi:10.1371/journal.pone.0006190)
Supplement: Table S11 — Wintonotitan wattsi - Vertebral measurements (mm) (0.05 MB DOC) [file pone.0006190.s014.doc]

***Wintonotitan wattsi***

Table S 11. Vertebral measurements (mm)

| Vertebra | Region | Centrum Length | Centrum Width | Centrum Height |
| --- | --- | --- | --- | --- |
| Dorsal | Posterior | 140+ | 280 | 180+ |
| Sacral | Posterior | 170 | 190 | 165+ |
| Caudal | Anterior | 145+ | 230 | 170+ |
| Caudal | Anterior | 140 | 210+ | 175 |
| Caudal | Anterior | 120+ | 215 | 150 |
| Caudal | Anterior | 120 | 200 | 170 |
| Caudal | Anterior | 134 | 200 | 150 |
| Caudal | Anterior | 138 | 170 | 130+ |
| Caudal | Anterior | 135 | 170 | 125 |
| Caudal | Anterior | 132 | 180 | 115+ |
| Caudal | Anterior | 130 | 160 | 135 |
| Caudal | Middle | 150 | 130 | 110+ |
| Caudal | Middle | 160 | 125 | 100+ |
| Caudal | Middle | 160 | 115 | 100 |
| Caudal | Middle | 140+ | 85 | 95+ |
| Caudal | Middle | 145 | 100+ | 90+ |
| Caudal | Middle | 130+ | 80 | 90 |
| Caudal | Middle | 140+ | - | - |
| Caudal | Distal | 125 | 85 | 80 |
| Caudal | Distal | 130 | - | - |
| Caudal | Distal | - | 75 | 75 |
| Caudal | Distal | 120 | 70 | 70 |
| Caudal | Distal | 120 | 75 | 70 |
| Caudal | Distal | 115 | 60 | 63 |
| Caudal | Distal | 110 | 65 | 62 |
| Caudal | Distal | 110 | 60 | 55 |
| Caudal | Distal | 100 | 45 | 50 |
| Caudal | Distal | 50+ | 45 | 45 |
