# Supplementary material for: New Mid-Cretaceous (Latest Albian) Dinosaurs from Winton, Queensland, Australia
Source: PLoS One. 2009 Jul 3;4(7):e6190. doi: 10.1371/journal.pone.0006190 (PMC2703565; doi:10.1371/journal.pone.0006190)
Supplement: Table S12 — Wintonotitan wattsi - Chevron measurements (mm) (0.03 MB DOC) [file pone.0006190.s015.doc]

***Wintonotitan wattsi***

Table S 12. Chevron measurements (mm)

| Chevron | Dorso-ventral Length | Antero-posterior length. | Proximal width. |
| --- | --- | --- | --- |
| 1 | 275 | 50 | 92 |
| 2 | 245+ | 35 | 73+ |
| 3 | 210+ | 26 | 68+ |
| 4 | - | 35 | 93 |
| 5 | 240+ | 40 | 90 |
