# Supplementary material for: New Mid-Cretaceous (Latest Albian) Dinosaurs from Winton, Queensland, Australia
Source: PLoS One. 2009 Jul 3;4(7):e6190. doi: 10.1371/journal.pone.0006190 (PMC2703565; doi:10.1371/journal.pone.0006190)
Supplement: Table S13 — Wintonotitan wattsi - Forearm measurements (mm) (0.03 MB DOC) [file pone.0006190.s016.doc]

***Wintonotitan wattsi***

Table S 13. Forearm measurements (mm).

|  | Maximum Length | Proximal Width | Distal Width | Mid-shaft Width |
| --- | --- | --- | --- | --- |
| Scapular Blade | 620mm + | 305mm+ | 300mm+ | 210mm+ |
| Acromion-Glenoid region | 510mm + | - | 600mm (across acromion) | 200mm+ |
| Left Humerus | 880mm + | 380mm + | - | 170mm + |
| Right Humerus | 920mm + | - | 390mm+ | 195mm |
| Left Ulna | 650mm | - | - | 145mm |
| Right Ulna | 890mm | 330mm |  | 150mm |
| Left Radius | 800mm + | ~210mm | 260mm | 130mm |
| Right Radius | 680mm + | - | 280 | 125 |
