# Supplementary material for: New Mid-Cretaceous (Latest Albian) Dinosaurs from Winton, Queensland, Australia
Source: PLoS One. 2009 Jul 3;4(7):e6190. doi: 10.1371/journal.pone.0006190 (PMC2703565; doi:10.1371/journal.pone.0006190)
Supplement: Table S14 — Wintonotitan wattsi - Metacarpal measurements (mm) (0.03 MB DOC) [file pone.0006190.s017.doc]

***Wintonotitan wattsi***

Table S 14. Metacarpal measurements (mm)

| Metacarpal | Length | Max. Proximal Width | Max. Distal Width | Mid-shaft Width |
| --- | --- | --- | --- | --- |
| Mc-I | 240mm + | 179mm + | - | 100mm + |
| Mc-II | 410mm | 110mm | 165mm | 105mm |
| Mc-III | 421mm | 140mm | 182mm | 96mm |
| Mc-IV | 375mm + | 120mm + | 150mm + | 98mm |
| Mc-V | 370mm | 140mm | 160mm | 100mm |
