# Supplementary material for: New Mid-Cretaceous (Latest Albian) Dinosaurs from Winton, Queensland, Australia
Source: PLoS One. 2009 Jul 3;4(7):e6190. doi: 10.1371/journal.pone.0006190 (PMC2703565; doi:10.1371/journal.pone.0006190)
Supplement: Table S15 — Wintonotitan wattsi - Ilium and Ischium measurements (mm) (0.03 MB DOC) [file pone.0006190.s018.doc]

***Wintonotitan wattsi***

Table S 15. Ilium and Ischium measurements (mm)

|  | Preacetabular Process Length | Preacetabular Process Width |  |  |
| --- | --- | --- | --- | --- |
| Left Ilium | 430mm | 210mm |  |  |
|  | Pubic blade – posterior margin Length | Dorso-ventral length of the pubic articular surface. | Pubic blade to iliac peduncle | Pubic blade – posterior process (distal end) |
| Left ischium | 240mm | 350mm+ | 430mm | 600mm+ |
