# Supplementary figures and images for: New Mid-Cretaceous (Latest Albian) Dinosaurs from Winton, Queensland, Australia
Source: PLoS One. 2009 Jul 3;4(7):e6190. doi: 10.1371/journal.pone.0006190 (PMC2703565; doi:10.1371/journal.pone.0006190)

***Australovenator wintonensis***

Table S 16. Tooth measurements (mm).


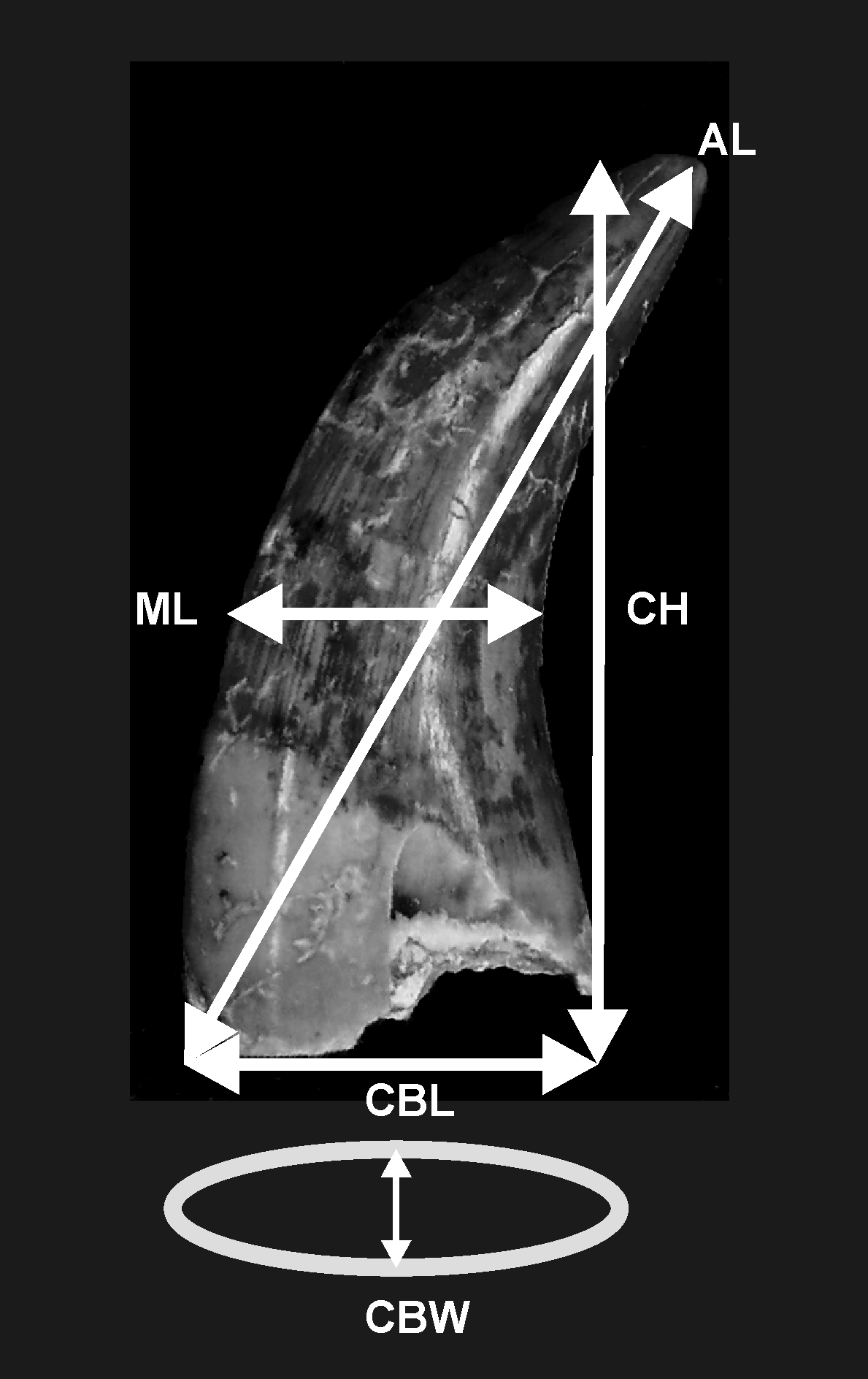


| AODF 604 | AL | CH | CBL | ML | CBW |
| --- | --- | --- | --- | --- | --- |
| -343a | 16.26 | 12.53 | 8.32 | 6.29 | 3.99 |
| -343b | 23.66 | 20.85 | 11.11 | 10.03 | 5.94 |
| -342 | 20.10 | 17.21 | 9.3 | 8.24 | 4.97 |
| -344 | 25.60 | 21.97 | 10.81 | 9.3 | 6.37 |
| -341 | 14.45 | 13.83 | 7.16 | 5.46 | 5.12 |
| -346 | - | 24.91 | - | 9.13 | - |
| -219 | 29.61+ | 23.36 | 12.62+ | 9.98 | 7.52 |
| -42 | 16.65 | 14.62 | 6.84 | 6.00 | 5.25 |
| -33 | - | 23.63+ | 10.55 | - | - |

Supplement: Table S16 — Australovenator wintonensis - Tooth measurements (mm) (0.31 MB DOC) [file pone.0006190.s019.doc]
