# Supplementary material for: New Mid-Cretaceous (Latest Albian) Dinosaurs from Winton, Queensland, Australia
Source: PLoS One. 2009 Jul 3;4(7):e6190. doi: 10.1371/journal.pone.0006190 (PMC2703565; doi:10.1371/journal.pone.0006190)
Supplement: Table S17 — Australovenator wintonensis - Dentary measurements (mm) (0.03 MB DOC) [file pone.0006190.s020.doc]

***Australovenator wintonensis***

Table S 17. Dentary measurements (mm).

| Dentary |  |
| --- | --- |
| Dorsal maximum length | 259 |
| Ventral maximum length | 243 |
| Length of Meckalian Groove (mg) | 135 |
| Dentary depth at anterior margin of mg | 34.09 |
| Posterior dentary depth | 69.95 |
| Anterior dentary width | 18.19 |
| Mid-dentary width | 15.03 |
| Posterior dentary width | 13.14 |
