# Supplementary material for: New Mid-Cretaceous (Latest Albian) Dinosaurs from Winton, Queensland, Australia
Source: PLoS One. 2009 Jul 3;4(7):e6190. doi: 10.1371/journal.pone.0006190 (PMC2703565; doi:10.1371/journal.pone.0006190)
Supplement: Table S18 — Australovenator wintonensis - Dorsal rib measurements (mm) (0.03 MB DOC) [file pone.0006190.s021.doc]

***Australovenator wintonensis***

Table S 18. Dorsal rib measurements (mm)

|  | Length | Capitulo-tubercular width | Capitulum length |
| --- | --- | --- | --- |
| Right dorsal rib 1 | 254+ | 95 | 29 |
| Right dorsal rib 2 or 3 | 182+ | 94 | 25 |
| Left dorsal rib 7 or 8 | 250+ | - | - |
