# Supplementary material for: New Mid-Cretaceous (Latest Albian) Dinosaurs from Winton, Queensland, Australia
Source: PLoS One. 2009 Jul 3;4(7):e6190. doi: 10.1371/journal.pone.0006190 (PMC2703565; doi:10.1371/journal.pone.0006190)
Supplement: Table S19 — Australovenator wintonensis - Ilium measurements (mm) (0.03 MB DOC) [file pone.0006190.s022.doc]

***Australovenator wintonensis***

Table S 19. Ilium measurements (mm)

| Ilium |  |
| --- | --- |
| Preacetabular process length | 163+ |
| Acetabulum length | 122 |
| Cranial width of acetabulum | 50 |
| Caudal width of acetabulum | 98 |
