# Supplementary material for: New Mid-Cretaceous (Latest Albian) Dinosaurs from Winton, Queensland, Australia
Source: PLoS One. 2009 Jul 3;4(7):e6190. doi: 10.1371/journal.pone.0006190 (PMC2703565; doi:10.1371/journal.pone.0006190)
Supplement: Table S20 — Australovenator wintonensis - Forearm measurements (mm) (0.04 MB DOC) [file pone.0006190.s023.doc]

***Australovenator wintonensis***

Table S 20. Forearm measurements (mm)

|  | Length | Prox. Width | Dist. Width | Mid-shaft Wid |
| --- | --- | --- | --- | --- |
| Right Ulna | 268 | 85.1  52.29 | 33.3  27.5 | 26.4  18.8 |
| Left Ulna | 269 | 89.19  48.02 | 37.02  30.5 | 29.5  19.01 |
| Left Radius | 213 | 40.09  25.79 | 44.03  29.83 | 21.32  20.19 |
| Left Metacarpal I | 78.38 | 39.47  21.46 | 38.14  Lat Med  35.15 28.17  14.4 18.2 | 29.87  19.04 |
| Left Mc I-1 | 117.82 | 34.66  30.06 | 42.81 36.66 | 26.46 20.84 |
| Right Mc I-1 | 117.54 | 33.6  28.98 | 38.04  34.74 | 28.15  20.92 |
| Right Mc II | 138.42 | 57.57  35.27 | 38.16  Lat Med  32.7 34.2  15.84 14.65 | 27.93  23.90 |
| Right Mc II-2 | 74.51 | 31.36  28.66 | 24.99  22.32 |  |
| Right Mc III-3 | 43.8 | 15.63 13.96 | 19.21  13.86 |  |
