# Supplementary material for: New Mid-Cretaceous (Latest Albian) Dinosaurs from Winton, Queensland, Australia
Source: PLoS One. 2009 Jul 3;4(7):e6190. doi: 10.1371/journal.pone.0006190 (PMC2703565; doi:10.1371/journal.pone.0006190)
Supplement: Table S21 — Australovenator wintonensis - Femur measurements (0.03 MB DOC) [file pone.0006190.s024.doc]

***Australovenator wintonensis***

Table S 21. Femur measurements

| Length | 578 |
| --- | --- |
| Proximal width from greater trochanter to head | 132 |
| Mid-shaft transverse width | 75 |
| Mid-shaft cranio-caudal length | 80 |
| Distal transverse width | 120 |
| Distal cranio-caudal length | 100 |
