# Supplementary material for: New Mid-Cretaceous (Latest Albian) Dinosaurs from Winton, Queensland, Australia
Source: PLoS One. 2009 Jul 3;4(7):e6190. doi: 10.1371/journal.pone.0006190 (PMC2703565; doi:10.1371/journal.pone.0006190)
Supplement: Table S22 — Australovenator wintonensis - Tibia measurements (0.03 MB DOC) [file pone.0006190.s025.doc]

***Australovenator wintonensis***

Table S 22. Tibia measurements

|  | Length | Proximal Width | Distal Width | Mid-shaft Circumference |
| --- | --- | --- | --- | --- |
| Right Tibia | 564 | 140 | 123 | 153 |
| Left Tibia | 569 | 135 | 138 | 165 |
