# Supplementary material for: New Mid-Cretaceous (Latest Albian) Dinosaurs from Winton, Queensland, Australia
Source: PLoS One. 2009 Jul 3;4(7):e6190. doi: 10.1371/journal.pone.0006190 (PMC2703565; doi:10.1371/journal.pone.0006190)
Supplement: Table S23 — Australovenator wintonensis - Fibula measurements (mm) (0.03 MB DOC) [file pone.0006190.s026.doc]

***Australovenator wintonensis***

Table S 23. Fibula measurements (mm).

| Fibula |  |
| --- | --- |
| Length | 538 |
| Proximal width | 110 |
| Distal width | 350 |
| Mid-shaft diameter | 59 |
