# Supplementary material for: New Mid-Cretaceous (Latest Albian) Dinosaurs from Winton, Queensland, Australia
Source: PLoS One. 2009 Jul 3;4(7):e6190. doi: 10.1371/journal.pone.0006190 (PMC2703565; doi:10.1371/journal.pone.0006190)
Supplement: Table S24 — Australovenator wintonensis - Astragalus measurements (mm) (0.03 MB DOC) [file pone.0006190.s027.doc]

***Australovenator wintonensis***

Table S 24. Astragalus measurements (mm).

| Astragalus |  |
| --- | --- |
| Ascending process height | 74 |
| Condylar width | 105 |
| Total Height | 102 |
| Cranio-caudal length of medial condyle | 64 |
