# Supplementary material for: New Mid-Cretaceous (Latest Albian) Dinosaurs from Winton, Queensland, Australia
Source: PLoS One. 2009 Jul 3;4(7):e6190. doi: 10.1371/journal.pone.0006190 (PMC2703565; doi:10.1371/journal.pone.0006190)
Supplement: Table S25 — Australovenator wintonensis - Metatarsal measurements (mm) (0.03 MB DOC) [file pone.0006190.s028.doc]

***Australovenator wintonensis***

Table S 25. Metatarsal measurements (mm).

|  | Length | Prox End | Distal End | Mid-shaft |
| --- | --- | --- | --- | --- |
| Left Mt I | 66 | n/a | 26.2  19.4 |  |
| Right Mt III | 322 | 79.43  36.94 | 53.54  47.45 | 29.98  22.68 |
| Right Mt II | 284 | 54.53 42.86 | 47.34  45.76 | 31.70  27.22 |
| Right Mt II-2 | 113.02 | 46.18  42.68 | 42.06  30.39 | 25.22  23.29 |
| Left Mt II-1 | 81.06 | 40.45  45.72 | 43.02  35.10 | 28.37  25.24 |
| Left Mt II-2 | 76.94 | 40.82  35.02 | 34.49  26.87 | 22.36  20.02 |
| Right Mt IV-3 | 40.69 | 28.99  27.8 | 23.49  18.95 |  |
| Right Mt IV-2 | 60.95+ | 35.00+  29.55 | 28.81+  24.99+ |  |
