# Supplementary material for: New Mid-Cretaceous (Latest Albian) Dinosaurs from Winton, Queensland, Australia
Source: PLoS One. 2009 Jul 3;4(7):e6190. doi: 10.1371/journal.pone.0006190 (PMC2703565; doi:10.1371/journal.pone.0006190)
Supplement: Table S26 — Australovenator wintonensis - Pes phalange and ungual measurements (mm) (0.03 MB DOC) [file pone.0006190.s029.doc]

***Australovenator wintonensis***

Table S 26. Pes phalange and ungual measurements (mm).

|  | Dorsal length | Ventral length | Proximal height | Proximal width |
| --- | --- | --- | --- | --- |
| Rt Mt IV-5 | 66.29 | 66.16 | 27.1 | 22.88 |
| Lt Mt II-3 | 69.48 | 71.07 | 30.81 | 25.45 |

| Ungual | Dorsal margin | Dorsal length | Ventral length | Proximal Height | Proximal Width |
| --- | --- | --- | --- | --- | --- |
| Right Mc I-2 | 190 | 150.95 | 112.09 | 59.84 | 26.08 |
| Right Mc III-4 |  | 75.12 | 62.14 | 31.10 | 13.15 |
